# Bis(silylene)‐Mediated N═N Bond Scission of Diazo Compounds

**DOI:** 10.1002/anie.3873649

**Published:** 2026-03-19

**Authors:** Yun Xiong, Shenglai Yao, Matthias Driess

**Affiliations:** ^1^ Department of Chemistry: Metalorganics and Inorganic Materials Technische Universität Berlin Berlin Germany

**Keywords:** cooperativity, diazodiphenylmethane, dinitrogen activation, N═N double bond, silylenes

## Abstract

Diazo compounds, >C═N═N, typically undergo C═N bond cleavage to form carbenes and N_2_, whereas direct N═N bond breaking remains exceedingly rare in main‐group chemistry. We report the N═N bond scission of diazodiarylmethanes, Ar_2_C═N═N (Ar = phenyl, Ar_2_C = 9,9‐fluorendiyl), mediated by a chelating bis‐NHSi (NHSi = N‐heterocyclic silylene). Three bis‐NHSi scaffolds with tunable Si···Si distances were examined: While **XT(LSi:)_2_** (XT = 9,9‐dimethyl‐9H‐xanthene‐4,5‐diyl, L = PhC(tBuN)_2_) and **PhN(LSi:)_2_** lead to end‐on addition products with partial N═N bond activation, the carborane‐based **CB(LSi:)_2_** (CB = 1,2‐C_2_B_10_H_10_) engenders complete N═N bond cleavage to afford isolable disilicon nitrido imino species containing a Si═N─Si─N═CAr_2_ unit that hydrolyzes to Ar_2_C═NH, NH_3_, SiO_2_, C_2_B_10_H_12_, and LH. According to density functional theory (DFT) calculations, the N═N cleavage stems from a unique cooperative interaction of the two divalent silicon centers.

## Graphical abstract

The carborane‐based bis(silylene) enables unprecedented N═N bond scission in diazodiarylmethanes. While xanthene‐ and aniline‐based scaffolds promote end‐on addition with partial N═N activation, the carborane‐based variant uniquely achieves complete N═N bond scission of Ar_2_C═N═N compounds, resulting in isolable disilicon nitride imino species. DFT calculations reveal that cooperative reorganization between the two divalent silicon centers governs this divergent reactivity, underscoring that bis(silylenes) act as versatile main‐group platforms for challenging N═N bond transformations.

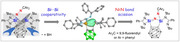

## 1 Introduction

**SCHEME 1:**
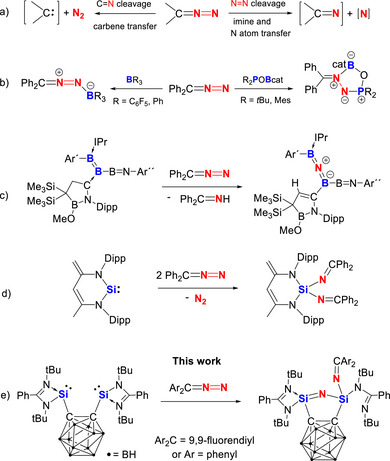
(a) C═N and N═N cleavage pathways of diazo compounds for carbene, imine and nitrogen atom transfer; (b) Partial N═N activation of Ph_2_C═N═N with boranes. (c) N═N bond cleavage of Ph_2_C═N═N mediated by a *N*‐heterocyclic carbene‐stabilized diborene (Ar´ = 4‐*tert*‐butylphenyl, Ar´´ = 4‐bromo‐2,6‐diisopropylphenyl, IPr = 1,3‐diisopropyl‐4,5‐dimethyl‐imidazol‐ylidene, Dipp = 2,6‐diisopropylphenyl); (d) N═N bond cleavage of Ph_2_C═N═N using a zwitterionic silylene; (e) bis(silylene)‐mediated N═N bond scission of Ar_2_C═N═N from this work.

Diazo compounds, >C═N═N, are key species in both organic synthesis and biochemical processes, well known for their ability to liberate highly reactive carbenes upon cleavage of the C═N bond with concurrent release of dinitrogen (Scheme 1a, left) [1, 2, 3, 4]. This reactivity has made diazo compounds to be indispensable reagents for a wide range of synthetic transformations. Conceptually, they can be viewed as carbenes coordinated to N_2_, thus providing a useful platform for modelling dinitrogen activation chemistry. To date, extensive studies have focused on C═N bond activation in diazo compounds for carbene transfer, the direct cleavage of the N═N bond to transfer imines and a nitrogen atom (Scheme 1a, right) remains a formidable challenge. Although several transition‐metal systems have demonstrated the ability to mediate such transformations [5, 6, 7, 8, 9, 10, 11, 12], examples involving main‐group elements are exceedingly rare. Within the Frustrated Lewis Pair (FLP) strategy for N─N bond activation [13, 14, 15, 16, 17], Stephan and co‐workers reported that diazodiphenylmethane reacts with boranes and intramolecular FLPs to form adducts in which the N═N double bond is activated but not cleaved (Scheme 1b) [13, 14, 15]. Recently, Kinjo et al. obtained a three‐membered AlN_2_ ring species from a cycloaddition reaction of a cyclic alkylamino aluminyl anion and diazodiarylmethane, which is thermally unstable and spontaneously releases N_2_ gas [18]. Shortly thereafter, the same group achieved the N═N bond cleavage of diazodiphenylmethane using an N‐heterocyclic carbene (NHC)‐supported boron–boron double bond system, yielding a C─H activation product containing a B─N─B unit along with Ph_2_C═NH (Scheme 1c) [19].

Silylenes, divalent silicon species, bearing a lone pair of electrons and an empty 3p orbital, are among the most versatile heavy main‐group compounds for mimicking transition‐metals for chemical transformations [20, 21, 22]. Utilizing the zwitterionic silylene developed by us previously [23], we could activate diazodiphenylmethane in a 1:2 molar ratio under release of N_2_ and isolated a product bearing a Si(N═CPh_2_)_2_ unit (Scheme 1d) [24]. The cooperative interaction between two silylene scaffolds within a single framework can significantly enhance their capacity for activating inert chemical bonds when the two Si^II^ atoms are in suitable proximity [25]. Over the past few years, our group has developed a series of chelating bis‐NHSi scaffolds (NHSi = *N*‐heterocyclic silylene), each incorporating two Si^II^ centers separated by various spacers with tunable Si···Si distances [26]. These bis(silylene) frameworks have proven highly effective in both the activation of small molecules [26, 27, 28, 29] and the stabilization of low‐valent main‐group species [25, 30, 31].

Using the aniline‐derived bis‐NHSi **PhN(LSi:)_2_** [32] [L = PhC(*t*BuN)_2_] with a Si···Si distance of 2.9 Å and the xanthene‐based analogue **XT(LSi:)_2_** [33] (XT = 9,9‐dimethyl‐9*H*‐xanthene‐4,5‐diyl) with a 4.3 Å separation, we previously demonstrated the activation of *cis*‐azobenzene Ph─N═N─Ph highlighting the cooperative role of the two silylene centers in N═N bond activation [34]. These results suggest that bis(silylenes) can act as efficient main‐group mediators for N = N bond cleavage, offering a new strategy for selective transformations of azobenzene. Inspired by these findings, we have now investigated the reactivity of bis‐NHSis toward two diazodiarylmethanes, as particularly challenging substrates for N═N bond activation. Remarkably, while the aniline‐based bis(silylene) **PhN(LSi:)_2_** [32] and the xanthene‐based **XT(LSi:)_2_** [33] react with Ar_2_C═N═N to give end‐on N═N‐addition products, the carborane‐based bis(silylene) **CB(LSi:)_2_** [35] (CB = 1,2‐C_2_B_10_H_10_) successfully effects complete N═N bond scission (Scheme 1e), leading to the formation of a complex containing a Si═N─Si─N═CPh_2_ unit that hydrolyzes to Ar_2_C═NH, NH_3_, SiO_2_, C_2_B_10_H_12_, and LH. Herein, we describe these unprecedented transformations, which demonstrate the usefulness of bis(silylenes) in N═N bond activation.

## 2 Results and Discussion

### 2.1 Activation of Diazodiarylmethanes With the Bis(silylene) XT(LSi:)_2_

**SCHEME 2:**
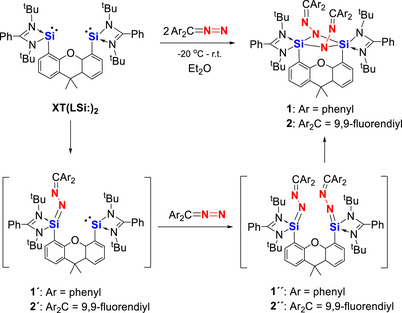
Reactions of xanthene‐based bis(silylene) **XT(LSi:)_2_** with diazodiarylmethanes to yield **1** and **2** via the proposed intermediates **1´**, **2´**, **1´´**, and **2´´**, respectively.

Initial experiments using an equimolar amount of **XT(LSi:)_2_** and Ar_2_C═N═N (Ar = phenyl or Ar_2_C = 9,9‐fluorendiyl) in diethyl ether showed that only half equivalent of bis(silylene) **XT(LSi:)_2_** was consumed. Complete conversion of the bis‐NHSi was achieved employing two equivalents of Ar_2_C═N═N, affording the yellow product **1** and orange **2** in 61% and 78% yield, respectively (Scheme 2). Crystals suitable for single‐crystal X‐ray diffraction (scXRD) analyses were obtained from toluene and n‐pentane solutions, respectively.

**FIGURE 1:**
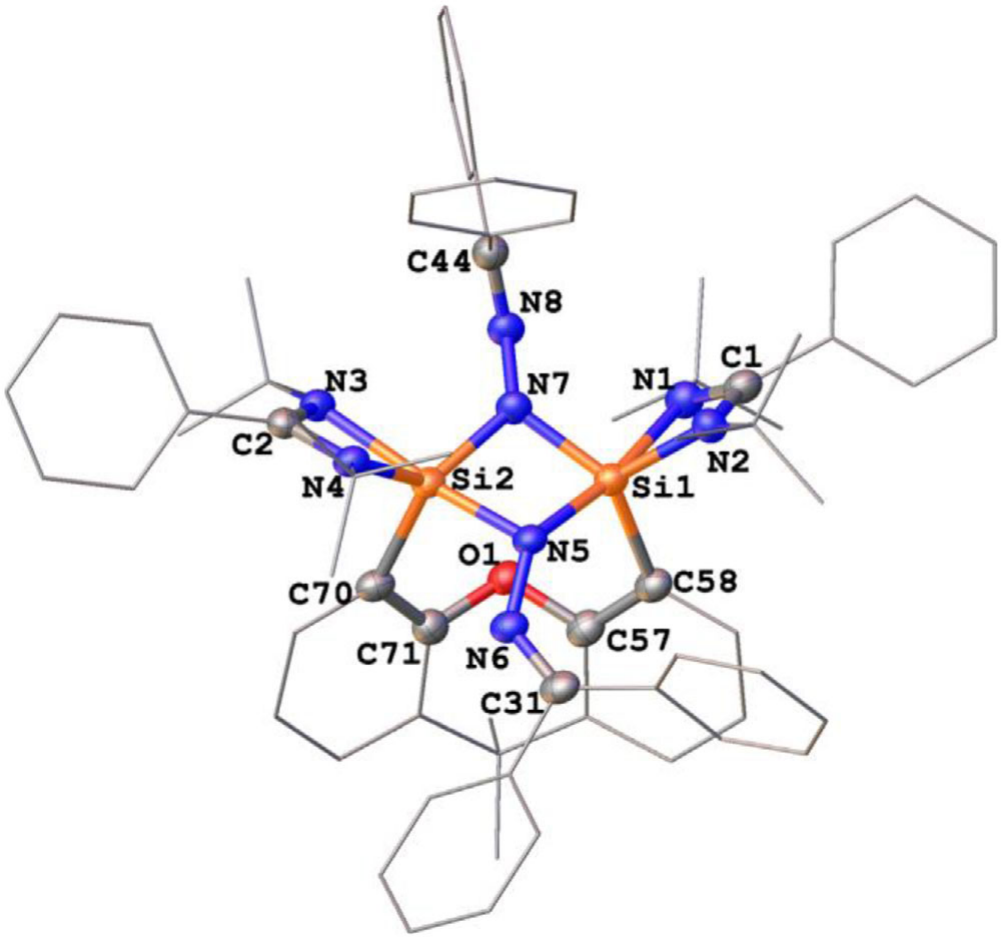
Molecular structure of **1** (Two independent molecules in the asymmetric unit are observed, only one is shown) [38]. Thermal ellipsoids are drawn at the 50% probability level. H atoms are omitted for clarity. Selected distances (Å) and angles (^o^) (The data in brackets are for the other independent molecule): Si1─N1 2.053(2) [2.100(2)], Si1─N2 1.830(2) [1.829(2)], Si1─N5 1.839(2) [1.829(2)], Si1─N7 1.776(2) [1.768(2)], Si1─C58 1.921(3) [1.909(3)], Si2─N4 2.054(2) [2.053(2)], Si2─N4 1.822(2) [1.830(2)], Si2─N5 1.823(2) [1.834(2)], Si2─N7 1.758(2) [1.758(2)], Si2─C70 1.941(3) [1.925(3)], N5─N6 1.371(3) [1.376(2)], N6─C31 1.295(3) [1.290(4)], N7─N8 1.414(3) [1.415(3)], N8─C44 1.282(4) [1.275(3)], N5─Si1‐N1 171.0(1) [173.50(9)], N2─Si1‐C58 107.8(1) [106.2(1)], N7─Si1─N2 114.1(1) [118.5(1)], N7─Si1─C58 134.9(1) [131.6(1)], N5─Si2─N3 172.8(1) [172.8(1)], N4─Si2‐C70 104.8(1) [106.1(1)], N7─Si2‐N4 125.6(1) [124.6(1)].

**FIGURE 2:**
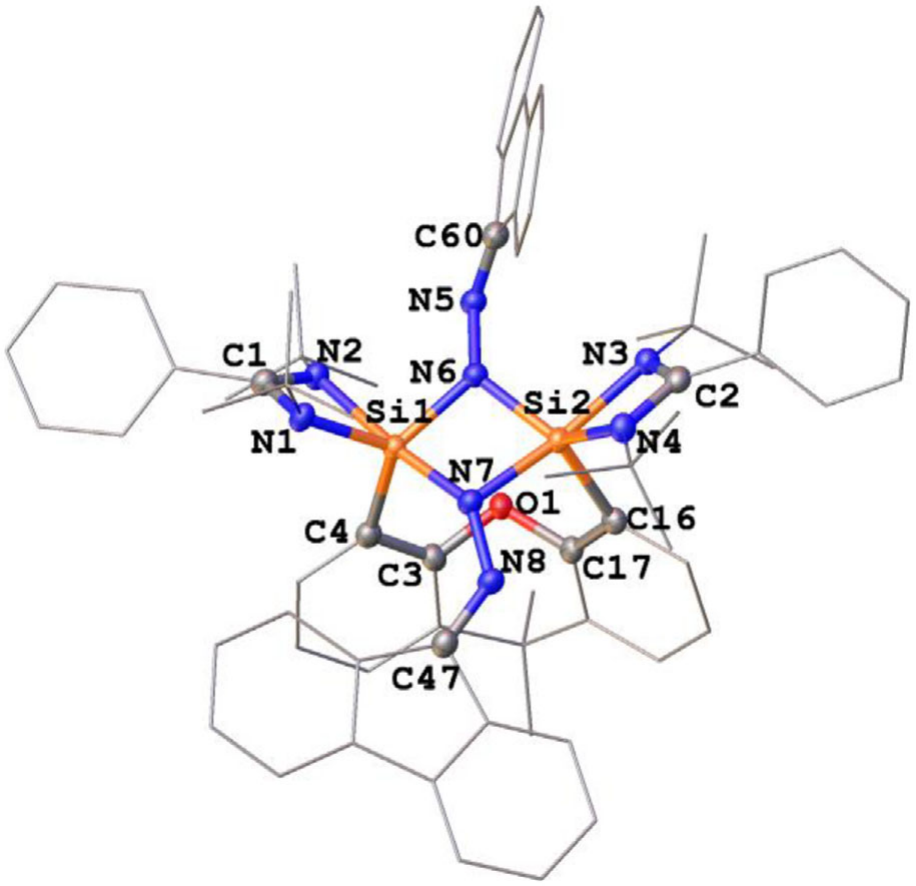
Molecular structure of **2** [38]. Thermal ellipsoids are drawn at the 50% probability level. H atoms are omitted for clarity. Selected bond lengths (Å) and angles (^o^): Si1─N1 1.813(1), Si1─N2 2.100(1), Si1─N6 1.773(1), Si1─N7 1.822(1), Si1─C4 1.919(1), Si2─N3 1.967(1), Si2─N4 1.846(1), Si2─N6 1.790(1), Si2─N7 1.830(1), Si2─C16 1.930(1), N5─N6 1.400(1), N7─N8 1.357(1), N5─C60 1.288(1), N8─C47 1.300(2). N(1)‐Si1─C4 108.79(5), N6─Si1‐N1 115.07(5), N6─Si1─C4 133.03(5), N7─Si1─N2 174.29(4), N6─Si2─N4 125.09(5), N7─Si2─N3 171.60(4), Si1─N6─Si2 100.49(5), Si1─N7─Si2 97.18(5).

The molecular structures of **1** and **2** exhibit similar structural features, characterized by a Si(*μ*‐N)_2_Si four‐membered ring in which each nitrogen atom is singly bonded to an N═CAr_2_ moiety via a N─N bond (Figures 1 and 2). The two silicon centers in both molecules are five‐coordinated and adopt a distorted trigonal‐bipyramidal coordination geometry. The Si─N distances of the four‐membered Si(*μ*‐N)_2_Si ring, spanning 1.758(2)‐1.839(2) Å, fall in the typical range for Si─N single bonds [34, 36, 37].

Due to the strongly puckered xanthene backbones, the four *tert*‐butyl groups in **1** and **2** each give rise to two singlets in their ^1^H NMR spectra (see Supporting Information). A similar splitting pattern is observed for the methyl groups attached at the xanthene body. Compound **1** exhibits a single ^2^ ^9^Si NMR resonance at *δ* = −94.2 ppm, consistent with chemically equivalent silicon centers in the molecule. In contrast, no ^2^ ^9^Si signal for **2** could be detected in the solution NMR spectrum owing to its poor solubility in polar and nonpolar nonprotic solvents. Solid state ^2^ ^9^Si NMR analysis of **2** revealed two resonances at *δ* = −91.8 and −105.3 ppm with values characteristic for five‐coordinate silicon(IV) centers [27, 34, 39]. The latter finding is in agreement with a scXRD analysis of **2**, which displays the absence of mirror symmetry between the two silicon moieties.

It should be noted that mono‐silylenes typically react with diazoalkanes to give end‐on addition products containing a Si═N─N═C< fragment as intermediates or final products [22, 40, 41]. The only known exception is the zwitterionic silylene mentioned above [23, 24]. It is thus reasonable to propose that **1′** and **2′**, as well as **1″** and **2″** (Scheme 2), are formed as corresponding intermediates. The latter intermediates **2′** and **2″** subsequently undergo isomerization to yield the final products **1** and **2**, respectively. This transformation is reminiscent of the reaction of the chlorosilylene **L(Cl)Si**: with diazodiphenylmethane, previously reported by Roesky and Stalke, which affords a dimeric product featuring the same Si(*μ*‐N─N═CPh_2_)_2_Si structural motif as observed in **1** and **2** [42]. Owing to the relatively large Si···Si separation (4.3 Å), the bis(silylene) **XT(LSi:)_2_** can be thus regarded as two nearly independent mono‐silylene centers exhibiting only limited cooperative interaction in their reaction with diazodiarylmethanes.

### 2.2 Activation of Diazodiarylmethanes With the Bis(silylenes) PhN(LSi:)_2_ and CB(LSi:)_2_

**SCHEME 3:**
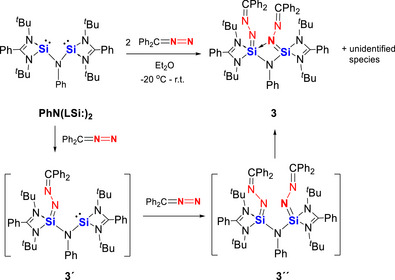
Reaction of the aniline‐based bis(silylene) **PhN(LSi:)_2_** with Ph_2_CN_2_ to afford **3** via the proposed intermediates **3´** and **3´´**.

**FIGURE 3:**
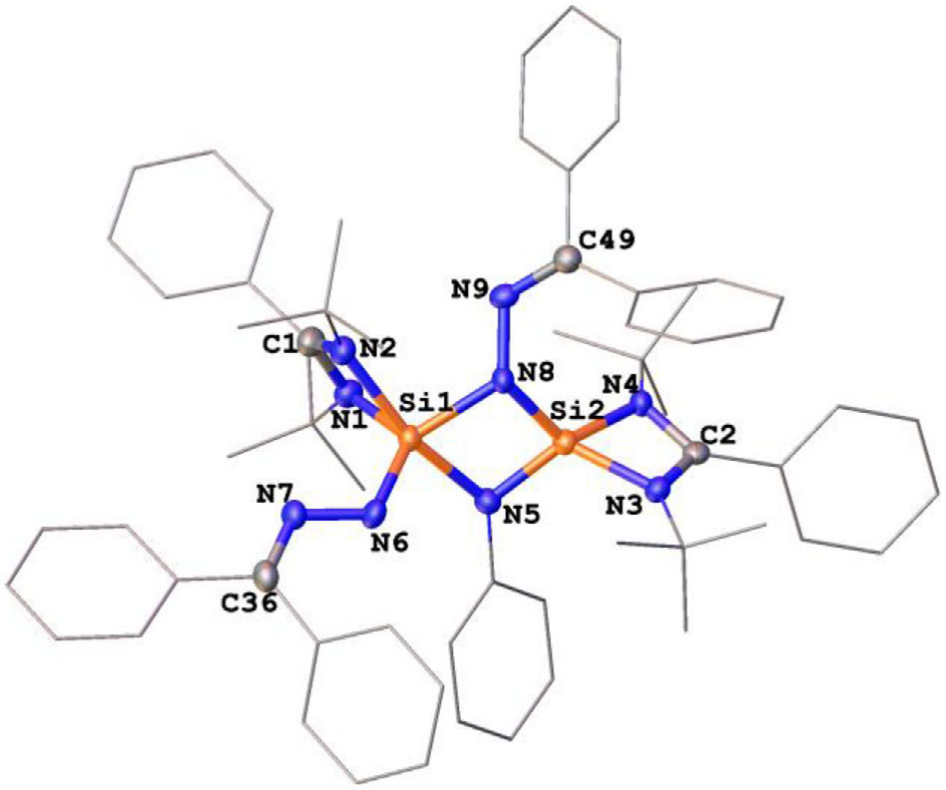
Molecular structure of **3** [38]. Thermal ellipsoids are drawn at the 50% probability level. H atoms and one toluene molecule as solvent are omitted for clarity. Selected bond lengths (Å) and angles (^o^) Si1─N1 1.965(2), Si1─N2 1.851(2), Si1─N5 1.887(2), Si1─N6 1.700(3), Si1─N8 1.856(2), Si2─N3 1.795(2), Si2─N4 1.795(2), Si2─N5 1.667(3), Si2─N8 1.685(2), N1─C1 1.288(4), N2─C1 1.376(4), N3─C2 1.335(4), N4─C2 1.359(3), N6─N7 1.353(3), N7─C36 1.311(4), N8─N9 1.405(3), N9─C49 1.291(4). N2─Si1─N1 68.9(1), N2─Si1─N5 104.9(1), N2─Si1─N8 112.4(1), N5─Si1─N1 165.5(1), N6─Si1─N1 99.4(1), N6─Si1─N2 121.5(1), N6─Si1─N595.0(1), N6─Si1─N8 125.2(1), N8─Si1─N1 90.7(1), N8─Si1─N5 79.5(1), N3─Si2─N4 73.7(1), N5─Si2─N3 117.2(1), N5─Si2─N4 127.6(1), N5─Si2─N8 91.1(1), N8─Si2─N3131.6(1), N8─Si2─N4120.5(1), Si2─N5─Si1 94.5(1), Si2─N8─Si1 95.0(1).

We further investigated the reactivity of **PhN(LSi:)_2_** toward diazodiphenylmethane. Preliminary results showed that **PhN(LSi:)_2_** reacts with Ph_2_CN_2_ in a 1:2 molar ratio to furnish **3** (Scheme 3). The molecular structure of **3** was determined by a scXRD analysis (Figure 3) and its composition confirmed by electrospray‐mass spectrometry (ESI‐MS). In contrast to compound **1** and **2**, in **3** only one of the Ph_2_CN_2_ entities is coordinated in an end‐on fashion to one silicon center while simultaneously interacting with the second silicon atom to form a N→Si bridge between the two silicon centers. Remarkably, the second Ph_2_CN_2_ is bound end‐on to a single silicon atom. Thus, one silicon atom is four‐coordinated and adopts a tetrahedral coordination geometry, whereas the other is five‐coordinated with a trigonal‐bipyramidal coordination environment. Notably, the two Si–N distances in the bridging Ph_2_CN_2_ unit differ drastically with Si1─N8 = 1.856(2) Å, and Si2─N8 = 1.685(2) Å, respectively. The former is slightly longer than a typical Si─N single bond, while the latter is even shorter than the Si1─N6 single bond distance (1.700(3) Å). These structural features indicate that the Si1─N8 interaction is best described as a dative N→Si bond, whereas the Si2─N8 interaction has Si═N double bond character.

Despite repeated efforts, analytically pure samples of compound **3** could not be obtained. The ^1^H NMR spectrum of the cleanest accessible material recorded at room temperature exhibits six *tert*‐butyl resonances (Figure S9a). Variable‐temperature ^1^H NMR measurements (253–333 K, Figure S9b) show no change in signal number below ambient temperature aside from minor chemical‐shift variations, whereas only four signals appear above 303 K, consistent with the sc‐XRD structure. The ^29^Si NMR spectrum shows two resonances (Figure S9c, *δ* = −108.6 and −109.3 ppm) that are unusually close in chemical shift for tetra‐ and pentacoordinate silicon centers, respectively. By contrast, DFT calculations predict well‐separated GIAO chemical shifts of −66.1 ppm for the tetracoordinate silicon atom and −103.1 ppm for the pentacoordinate silicon atom. These findings suggest rapid exchange between the coordination environments of the two silicon atoms, potentially involving fast amidinato N‐donor exchange and/or positional interchange of the Ph_2_CN_2_​ ligands. Such dynamic behavior likely also contributes to the difficulty in isolating analytically pure compound **3**. Nevertheless, analogous to the conversion of **XT(LSi:)_2_** with Ph_2_CN_2_, **3′** and **3′′** may appear as key intermediates in accordance with results by DFT calculations (Figure S32). In this case, only one of the Si = N moieties coordinates to the second silicon atom, resulting in a distinct structural motif similar to that observed previously for the reaction product with PhN═NPh [34].

**SCHEME 4:**
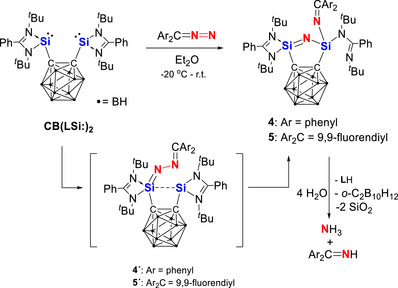
Reactions of bis(silylene) **CB(LSi:)_2_** with Ar_2_CN_2_ to afford **4** and **5** via intermediates **4´** and **5´**, respectively.

Interestingly, the reaction of **CB(LSi:)_2_** with Ar_2_CN_2_ showed a markedly different reactivity pattern. No product corresponding to a 1:2 stoichiometric ratio was detected; instead, a clean equimolar reaction took place, affording **4** which features a fully cleaved N═N bond (Scheme 4). Compound **4** was isolated as yellow crystals in 60% yield. Likewise, the reaction of **CB(LSi:)_2_** with 9‐diazofluorene proceeded in a 1:1 stoichiometric manner to afford the orange product **5** in 55% isolated yield. In the ^1^H NMR spectra of both **4** and **5**, four *tert*‐butyl groups appear as four singlets with comparable chemical shifts for the two compounds. Consistent with these observations, the ^2^ ^9^Si{^1^H} NMR spectra of each compound exhibit two distinct silicon resonances (*δ* = −29.3 and −41.4 ppm for **4**, and *δ* = −29.0 and −41.0 ppm for **5**), confirming the presence of two silicon centers in different coordination environments.

**FIGURE 4:**
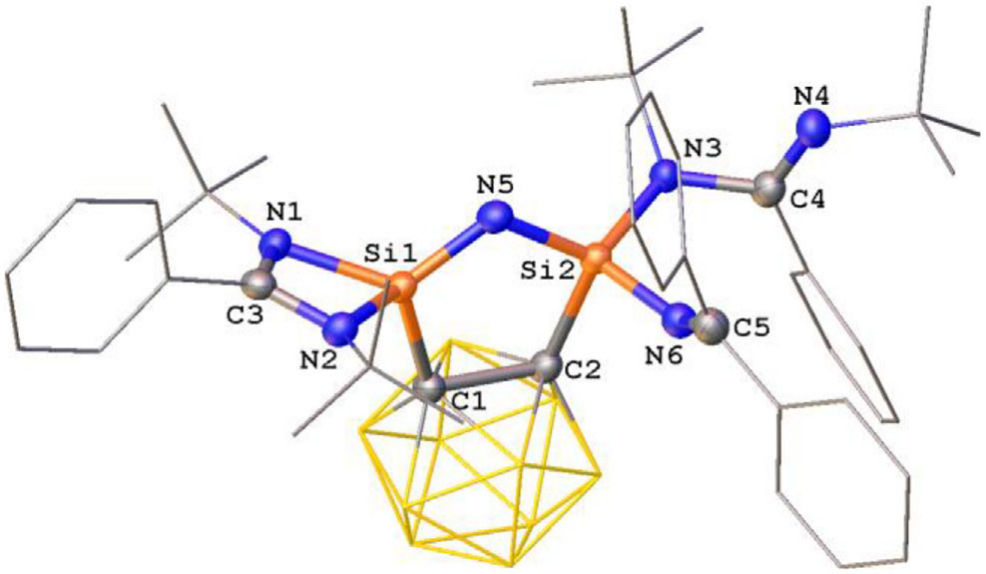
Molecular structure of **4** [38]. Thermal ellipsoids are drawn at the 50% probability level. H atoms and solvent molecules are omitted for clarity. Selected bond lengths (Å) and angles (^o^): Si1─N1 1.833(2), Si1─C1 1.910(2), Si1─N2 1.819(2), Si1─N5 1.622(2), C1─C2 1.717(3), Si2─C2 1.973(2), Si2─N3 1.749(2), Si2─N5 1.681(2), Si2─N6 1.718(2), N4─C4 1.271(3), C5─N6 1.270(3). N1─Si1─C1 113.61(9), N2─Si1‐N1 72.35(8), N2─Si1─C1 113.74(8), N5─Si1─N1 124.74(9), N5─Si1─C1 107.00(9), N5─Si1─N2 122.29(9), N5─Si2─C2 101.94(9), N5─Si2─N3 117.47(9), N5─Si2─N6 119.29(9), N6─Si2─C2 99.31(9), N6─Si2─N3 104.02(9).

**FIGURE 5:**
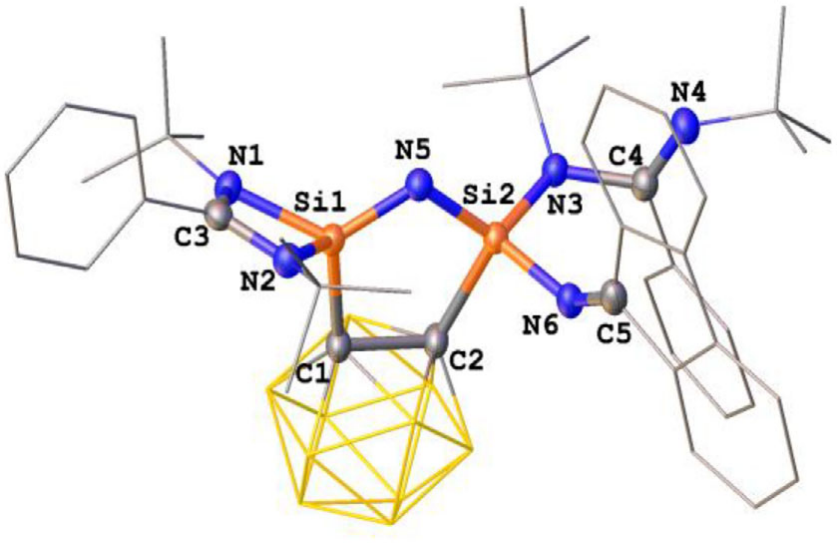
Molecular structure of **5** [38]. Thermal ellipsoids are drawn at the 50% probability level. H atoms and solvent molecules are omitted for clarity. Selected bond lengths (Å) and angles (^o^): Si1─N5 1.617(1), Si1─N2 1.808(1), Si1─N1 1.831(1), Si1─C1 1.906(1), Si2─N3 1.737(1), Si2─N5 1.674(1), Si2─N6 1.728(1), Si2─C2 1.968(1), N3─C4 1.447(2), N4─C4 1.264(2), N6─C5 1.262(2), C2─C1 1.711(2). N1─Si1─C1 113.02(5), N5─Si1─N1 125.27(5), N5─Si1─N2 121.25(5), N5─Si1─C1 107.17(5), N2─Si1─N1 72.62(5), N2─Si1─C1 114.41(6), N3─Si2─C2 116.78(5), N5─Si2─N3 115.94(5), N5─Si2─N6 120.44(6), N5─Si2─C2 101.83(5), N6─Si2─N3 103.31(5), N6─Si2─C2 97.80(5), C4─N3─Si2 115.41(8), Si1─N5─Si2 116.84(6).

The molecular structures of **4** and **5** were established by scXRD analyses (Figures 4 and 5). In both compounds, the two silicon atoms are interconnected by a single nitrogen atom, giving rise to a novel five‐membered C_2_Si_2_N ring with a Si═N−Si sequence. Both silicon centers are four‐coordinate and adopt a distorted tetrahedral coordination geometry; however, their ligand environments differ. Apart from the carborane spacer, one silicon atom is chelated by a bidentate amidinato ligand, whereas the other is coordinated by a monodentate amidinato ligand and a N═CAr_2_ scaffold. A striking feature of these molecular structures is the difference in the Si─N distances involving the bridging nitrogen atom derived from the diazomethanes. In compound **4**, the Si1─N5 bond [1.622(2) Å] is shorter than Si2─N5 [1.681(2) Å] and also shorter than those of the terminal Si─N bonds Si2─N3 [1.749(2) Å] and Si2─N6 [1.718(2) Å], respectively. A similar trend is observed in **5**, where Si1─N5 [1.617(1) Å] is notably shorter than Si2─N5 [1.674(1) Å], and again shorter than Si2─N3 [1.737(1) Å] and Si2─N6 [1.728(1) Å], respectively. These differences suggest partial Si═N double bond character of the Si1–N5 bond for both structures, whereas the Si2─N5 bond length represent a single bond. This structural motif and the observed N═N bond scission is reminiscent of our previous study on the reaction of **CB(LSi:)_2_** with adamantyl azide, leading to N═N bond cleavage of the azide group to afford a bis(silylene)‐supported N^I^ complex under redox conditions [43].

The reactivity of **4** and **5** toward water was investigated at room temperature (Scheme 4). The color of the solutions faded immediately. The imino and nitrido units hydrolyze cleanly to form Ar_2_C═NH and NH_3_, respectively, accompanied by the generation of **L**H, 1,2‐C_2_B_10_H_12_, and SiO_2_ as evidenced by multinuclear NMR spectroscopy (Figures S20–S25). When D_2_O was used, the corresponding Ar_2_C═ND and ND_3_ products were unambiguously identified by ^1^H and ^2^H NMR spectroscopy (Figures S23 and S24).

### 2.3 Density Functional Theory Calculations

**FIGURE 6:**
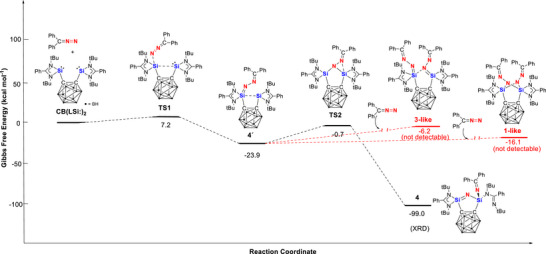
DFT‐derived mechanism for the reaction of **CB(LSi:)_2_** with diazodiphenylmethane to give the N═N cleavage product **4** via the intermediate **4´**, and the hypothetic 1:2 products **1‐like** and **3‐like**.

Based on the molecular structures of the isolated products and the aforementioned reactivity patterns of silylenes toward diazomethanes, **4′** and **5′** are proposed as plausible intermediates which subsequently transform to the final products **4** and **5** through insertion of the second silylene moiety into the N–N bonds in **4′** and **5′**, respectively (Scheme 4). The DFT‐derived mechanism for the reaction of **CB(LSi:)_2_** with diazodiphenylmethane, calculated at the SMD‐PW6B95‐D4/def2‐TZVP//r^2^SCAN‐3c level of theory (see *SI* for details), is depicted in Figure 6.

The reaction begins with the end‐on coordination of diazodiphenylmethane to one silylene unit of **CB(LSi:)_2_**, yielding the intermediate **4´**, which features a Si═N–N═CPh_2_ fragment (ΔG = −23.9 kcal mol^−^^1^). This reaction proceeds via **TS1**, characterized by electron donation from a silicon lone pair of electrons into the N═N π* orbital in the diazomethane as indicated by the intrinsic bond orbital analysis (IBO) [44, 45] (Figure S31). The latter donor–acceptor interaction is also supported by NBO analysis (Figures S36 and S37) [46]: Second‐order perturbation reveals a stabilization energy of 22.86 kcal mol^−^^1^ for the silicon lone pair to the N═N π* orbital and 33.26 kcal mol^−^^1^ to the vacant 3p orbital of the second silicon atom (Figure S37).

In addition, pronounced donation from the terminal nitrogen lone pair into vacant orbitals at the silicon atom (26.13 and 11.13 kcal mol^−^^1^) is observed. The computed small barrier (7.2 kcal mol^−^^1^) is consistent with the experimentally observed high reactivity of the bis(silylene) species toward diazodiphenylmethane. Subsequently, **4´** undergoes an isomerization (Δ*G* ^‡^ = 23.2 kcal mol^−^^1^) to afford **4** in a highly exothermic step (Δ*G* = −99.0 kcal mol^−^^1^), during which the N═N bond is fully cleaved and both nitrogen atoms bond to the second silicon center.

This finding raises a mechanistic question: Although the aniline‐based bis‐NHSi **PhN(LSi:)_2_** possesses an even shorter Si···Si separation than **CB(LSi:)_2_**, why does **PhN(LSi:)_2_** not achieve the similar N═N bond‐cleavage product? To address this, an analogous two‐step reaction pathway leading to the hypothetical N = N scission product **III** (Figure S32) was studied computationally. The initial 1:1 addition to form intermediate **3′** parallels the formation of **4´**, again proceeding via Si:→ π*(N═N) donation and exhibiting a low activation barrier (4.7 kcal mol^−^^1^) (Figure S33). However, the electronic structures of the resulting intermediates diverge significantly. In **3′**, only one silylene unit coordinates to diazodiphenylmethane, while the second silicon atom remains divalent, as indicated by its active lone pair in the HOMO and HOMO‐1 (Figure S34) and the corresponding IBO2 (Figure S35). The Si···Si distance of 3.0 Å also expands slightly relative to the ‘free’ bis‐NHSi **PhN(LSi:)_2_** (2.9 Å).

**FIGURE 7:**
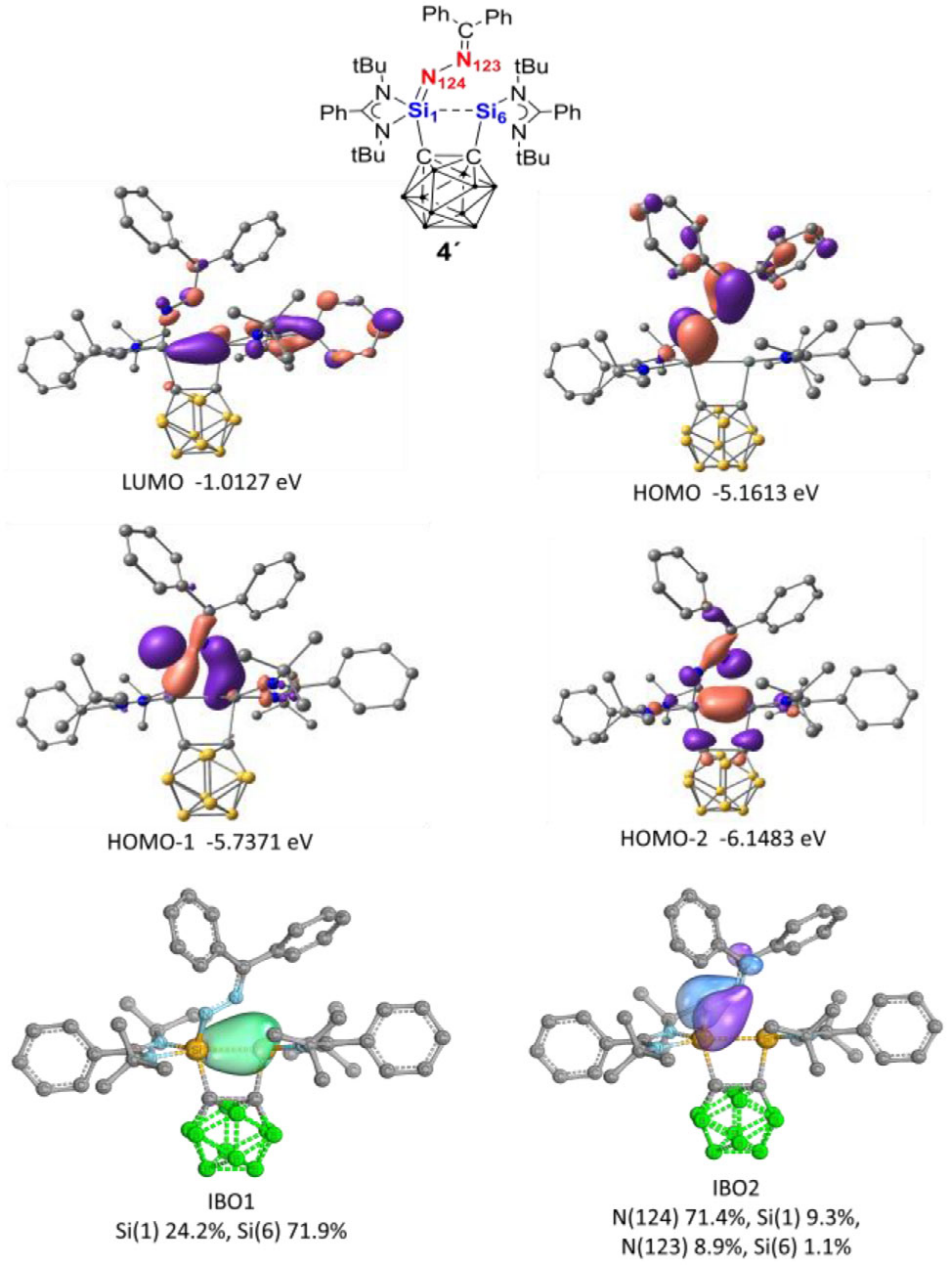
Selected frontier molecular orbitals and IBOs of **4´** (Hydrogen atoms are omitted for clarity), illustrating the Si═N double bond and partial Si–Si bond formation as well as the loss of lone‐pair character at the second silicon center (Si_6_).

In contrast, **CB(LSi:)_2_** undergoes substantial structural reorganization upon forming **4´**; the Si···Si distance contracts from 3.3 Å to 2.5 Å, and a Si─Si bond with approximate bond order of 0.5 is generated. NBO analysis indicates that the newly formed Si─Si interaction in **4´** corresponds to a σ‐bonding orbital with an occupancy of 1.90e, with the electron predominantly localized on the second silicon atom (Figure S38). Furthermore, second‐order perturbation analysis reveals significant donor–acceptor interactions (14.59 and 11.68 kcal mol^−^^1^) between lone pair of the coordinated N–N unit and vacant orbitals on the second silicon atom, which further stabilize intermediate **4´** (Figure S39). Consequently, the second silicon atom loses its divalent lone‐pair character, as evidenced by the frontier molecular orbitals (HOMO, HOMO‐1, and HOMO‐2), IBO analyses (Figure 7) and NBO results (Figure S38).

To evaluate the stability of intermediates **3´** and **4´** with respect to their rotational conformers, relaxed potential energy surface scans were performed around their central dihedral angles Si─Si─N─N. In both cases, **3´** and **4´** were found to be more stable than the alternative rotational conformers (Figure S40). As a result of retaining a reactive lone pair, intermediate **3′** preferentially undergoes a 1:2 reaction with a second equivalent of diazodiphenylmethane to furnish **3′′** (Δ*G* ^‡^ = 8.3 kcal mol^−^^1^) rather than isomerizing to the hypothetical N═N‐scission product **III** (Figure S32). The latter pathway requires a prohibitive barrier of 50.7 kcal mol^−^^1^, rendering N═N bond cleavage inaccessible under the current reaction conditions. This stands in stark contrast to the carborane‐based bis‐NHSi **CB(LSi:)_2_**, which readily proceeds to N = N scission due to its ability to reorganize and engage both silicon centers cooperatively in chemical bond activation. Thus, although **PhN(LSi:)_2_** displays a shorter Si···Si separation in the scXRD structure, this feature alone does not appear sufficient to enforce the degree of Si···Si contraction and electronic reorganization required for N = N bond scission during the reaction process. By comparison, the carborane framework in **CB(LSi:)_2_** provides a more rigid geometric constraint that may facilitate cooperative participation of both divalent silicon centers and thereby support N═N bond activation and cleavage. Furthermore, conversion of intermediate **4′** with a second equivalent of diazodiphenylmethane to give a hypothetical **3‐like** product is calculated to be thermodynamically unfavorable (Δ*G* = +17.7 kcal mol^−^^1^), plausibly reflecting steric congestion (Figure 6), and formation of a **1‐like** 1:2 adduct is likewise endothermic (ΔG = +7.8 kcal mol^−^^1^). Both species are therefore significantly less stable than the experimentally observed product **4** and are not expected to be detectable under the reaction conditions. Taken together, these observations suggest that not only the Si···Si distance and the rigidity of **CB(LSi:)_2_**, but also the steric and electronic characteristics of the C,C′‐dicarborandiyl linker, govern the observed selectivity for N═N bond cleavage.

## 3 Conclusions

In summary, we have uncovered the N═N bond scission in diazodiarylmethanes (Ar_2_C═N═N) utilizing a carborane‐based bis(silylene). Remarkably, the two silylene units in the xanthene‐based bis(silylene) **XT(LSi:)_2_**, featuring a relatively large Si···Si separation (4.3 Å), behave nearly independent and react each with one diazodiarylmethane molecule to give the end‐on addition products **1** and **2**, respectively. Similarly, the aniline‐based bis(silylene) **PhN(LSi:)_2_**, despite its shorter Si···Si distance (2.9 Å), also undergoes end‐on addition with two molar equivalents of Ph_2_C═N═N, yielding the N═N activation product **3**. In sharp contrast, the carborane‐based bis(silylene) **CB(LSi:)_2_**, with an intermediate Si···Si separation of 3.3 Å, reacts with only one equivalent of diazodiarylmethane under complete N═N bond cleavage, producing **4** and **5**, which upon hydrolysis furnish Ar_2_C═NH and NH_3_. DFT calculations attribute this divergent reactivity to a distinctive cooperative interaction between the two divalent silicon centers supported by the carborane framework. Unlike the reaction of **PhN(LSi:)_2_** with Ph_2_C═N═N in the molar ratio of 1:1, the reaction of **CB(LSi:)_2_** triggers a significant structural reorganization that transiently enhances Si···Si interaction, diminishes the lone‐pair character at the second silicon(II) center, and provides a low barrier pathway for N═N bond rupture. Overall, these results showcase that rationally designed bis(silylene) architectures can access previously inaccessible modes of small molecule activation. The striking role of cooperative silylene units in bis(silylenes) for promoting challenging scission of N─N multiple bonds will be further studied in our laboratory.

## Conflicts of Interest

The authors declare no conflicts of interest.

## Supporting information




**Supporting File 1**: The authors have cited additional references within the Supporting Information [47, 48, 49, 50, 51, 52, 53, 54, 55, 56, 57, 58, 59, 60, 61, 62, 63].

## Data Availability

The data that support the findings of this study are openly available in the supporting information.
