# Supplementary material for: Bis(silylene)‐Mediated N═N Bond Scission of Diazo Compounds
Source: Angew Chem Int Ed Engl. 2026 Mar 19;65(18):e3873649. doi: 10.1002/anie.3873649 (PMC13110765; doi:10.1002/anie.3873649)
Supplement: Supplementary file 1 — Supporting File 1: The authors have cited additional references within the Supporting Information [47, 48, 49, 50, 51, 52, 53, 54, 55, 56, 57, 58, 59, 60, 61, 62, 63]. [file ANIE-65-e3873649-s001.pdf]

Supporting Information

Bis(silylene)-Mediated N=N Bond Scission of Diazo Compounds

Yun Xiong, Shenglai Yao, and Matthias Driess*

Table of Content

A. Experimental Section…………………………………………….………………...…...……...S2

A1 General Considerations……………………………………….……….…….……….……S2

A2 Single-Crystal X-ray Structure Determination………………………...…...….……….…..S2

A3 Synthesis and Characterization………………………………….…………….………..….S3

A4 Details of the Single Crystal X-ray Diffraction Analyses…………………….……..…….…S22

B. Computational Details …………….…………….……………….….…….… …....……..S35

C. References………………………………………………….……….….….……....…......S80

A. Experimental Section

A1. General Considerations

All experiments were carried out under dry oxygen-free nitrogen using standard Schlenk techniques or an MBraun glove box fitted with a gas purification and recirculation unit. Solvents were dried by standard methods and freshly distilled prior to use. The starting material **PhN(LSi:)$_2$**[1] [L = PhC(N^{t}Bu)$_2$], **CB(LSi:)$_2$**[2] [CB = 1,2-dicarboranediyl], and **XT(LSi:)$_2$**[3] [XT = 9,9-Dimethyl-9H-xanthene-4,5-diyl] were prepared according to the literature procedures. Diazodiphenylmethane[4] and 9-diazo-9*H*-fluorene[5] was synthesized according to literature procedures. The NMR spectra were recorded with Bruker spectrometers Avance II 400, Avance III 400, and Avance III 500 referenced to residual solvent signals as internal standards. Abbreviations: *s* = singlet; *d* = doublet; *t* = triplet; *sept* = septet; *m* = multiplet; *br* = broad. High-resolution ESI-MS were measured on a Thermo Scientific LTQ orbitrap XL. IR spectra were measured with a Nicolet iS5 FT-IR-Spectrometer from the company of Thermo Scientific. Melting points were measured on a Stuart SMP30 melting point apparatus.

A2. Single-Crystal X-ray Structure Determination

The crystals were mounted on a glass capillary in per-fluorinated oil and measured in a cold N_2 flow. The data of **1**, **2**, **3**, **4**, and **5** were collected on an Oxford Diffraction Supernova, Single source at offset, Atlas at 150 K (Cu- Kα-radiation, λ = 1.5418 Å). The structures were solved with the SHELXT[6] and refined with Olex2[7,8] software package. The positions of the H atoms were calculated and considered isotropically according to a riding model. In the molecular structure of compound **1**, one of the phenyl ring is disordered over two orientations with an occupancy ratio of 0.58:0.42. Three diethyl ether molecules can be found in the asymmetric unit, but they are severely disordered and have been removed with the solvent-mask procedure in Olex2.[7] In the molecular structure of compound **2**, 0.5 n-pentane molecule can be found in the asymmetric unit, but it is severely disordered and has been removed with the solvent-mask procedure in Olex2. In the molecular structure of compound **3**, three toluene molecules can be found in the asymmetric unit, but two of these are severely disordered and have been removed with the solvent-mask procedure in Olex2. In the molecular structure of compound **4**, one toluene solvent molecule is found in the asymmetric unit. In the molecular structure of compound **5**, one of the *tert*-butyl group is disordered over two orientations with an occupancy ratio of 0.58:0.42. Deposition CCDC 2506159 (compound **1**), 2506156 (compound **2**), 2506158 (compound **3**), 2506155 (compound **4**) and 2506157 (compound **5**) contain the supplementary crystallographic data for this paper. These data are provided free of charge by the joint Cambridge Crystallographic Data Centre and Fachinformationszentrum Karlsruhe Access Structures service.

A3. Synthesis and Characterization

Compound 1: To a solution of 0.41 g (0.56 mmol) of **XT(LSi:)₂** in Et_2O (25 mL) at -20 °C was added a solution of 0.11 g (0.56 mmol) of Ph_2CN_2 in Et_2O (10 ml) dropwise while stirring. After 10 minutes, a yellow precipitate was observed. The cooling bath was removed, and the reaction mixture was stirred overnight. ^{1}H NMR analysis revealed that only half of the starting material **XT(LSi:)₂** had reacted, leading to the formation of yellow crystals of compound **1**. To drive the reaction to completion, an additional 0.11 g (0.56 mmol) of Ph_2CN_2 in Et_2O (10 ml) was added, and the mixture was stirred for another night. Volatiles were removed under reduced pressure and the residue was washed with n-pentane. The crude product was dissolved in diethyl ether and stored at 4 °C, which yielded 0.38 g (0.34 mmol, 61%) of compound **1** as yellow crystals after vacuum drying. **M.p.** 207 °C (decomp.). **^{1}H NMR** (400 MHz, THF-*d*$_8$, 298K): δ = 0.80 (*s*, 18 H, C(C*H*$_3$)$_3$), 1.02 (*s*, 18 H, C(C*H*$_3$)$_3$), δ = 1.62 (*s*, 3 H, C*H*$_3$), 1.69 (*s*, 3 H, C*H*$_3$), 5.51 (*d*, $^3J_{\text{(H, H)}}$ = 7.4 Hz, 2 H, arom.-*H*), 6.60 (*t*, $^3J_{\text{(H, H)}}$ = 7.6 Hz, 2 H, arom.-*H*), 6.71 (*t*, $^3J_{\text{(H, H)}}$ = 7.2 Hz, 1 H, arom.-*H*), 6.83 – 6.86 (*m*, 2 H, arom.-*H*), 7.10 (*t*, $^3J_{\text{(H, H)}}$ = 7.6 Hz, 2 H, arom.-*H*), 7.14 – 7.16 (*m*, 3 H, arom.-*H*), 7.24 (*d*, $^3J_{\text{(H, H)}}$ = 8.0 Hz, 2 H, arom.-*H*), 7.28 – 7.30 (*m*, 3 H, arom.-*H*), 7.37 – 7.38 (*m*, 4 H, arom.-*H*), 7.42 – 7.44 (*m*, 7 H, arom.-*H*), 7.57 (*t*, $^3J_{\text{(H, H)}}$ = 7.2 Hz, 2 H, arom.-*H*), 7.66 – 7.68 (*m*, 2 H, arom.-*H*), 7.74 – 7.76 (*m*, 2 H, arom.-*H*), 7.91 ppm (*d*, $^3J_{\text{(H, H)}}$ = 8.0 Hz, 2 H, arom.-*H*). **^{1}H NMR** (400 MHz, C_6D_6, 298K): δ = 0.93 (*s*, 18 H, C(C*H*$_3$)$_3$), 1.25 (*s*, 18 H, C(C*H*$_3$)$_3$), δ = 1.67 (*s*, 3 H, C*H*$_3$), 1.82 (*s*, 3 H, C*H*$_3$), 5.97 (*d*, $^3J_{\text{(H, H)}}$ = 7.5 Hz, 2 H, arom.-*H*), 6.84 – 6.94 (*m*, 10 H, arom.-*H*), 7.09 – 7.34 (*m*, 14 H, arom.-*H*), 7.43 (*t*, $^3J_{\text{(H, H)}}$ = 7.8 Hz, 2 H, arom.-*H*), 7.57 (*d*, $^3J_{\text{(H, H)}}$ = 7.0 Hz, 2 H, arom.-*H*), 7.67 (*d*, $^3J_{\text{(H, H)}}$ = 6.8 Hz, 2 H, arom.-*H*), 8.01 (*d*, $^3J_{\text{(H, H)}}$ = 7.8 Hz, 2 H, arom.-*H*), 8.04 ppm (*d*, $^3J_{\text{(H, H)}}$ = 7.4 Hz, 2 H, arom.-*H*). **^{13}C{^{1}H} NMR** (100 MHz, C_6D_6, 298K): δ = 22.5, 29.6 (C(*C*H$_3$)$_2$), 31.1, 32.7 (NC(*C*H$_3$)$_3$), 38.0 (*C*Me$_2$), 53.4, 54.5 (N*C*Me$_3$), 122.1, 122.4, 125.3, 126.4, 126.7, 126.9, 127.0, 127.24, 127.3 (Ph-*C*), 127.5, 127.6, 127.8 (Ph-*C*), 128.0, 128.1, 128.6, 128.9, 129.6, 129.9, 130.2, 130.4, 131.3, 133.5, 135.6 (Ph-*C*), 136.4 (Ph-*C*), 137.9 (Ph-*C*), 138.0 (Ph-*C*), 141.2 (Ph-*C*), 141.6 (Ph-*C*), 144.5 (Ph-*C*), 145.1 (Ph-*C*), 160.5 (N*C*N), 164.6 (N*C*N), 168.2 ppm (*C*Ph$_2$). **^{29}Si{^{1}H} NMR** (79 MHz, C_6D_6, 298 K): δ = -94.2 ppm (*s*, *Si*). **HR-ESI-MS: m/z**: 1115.5800 (*calc.* 1115.5865 [M]$^+$, m/z); **IR** (cm^{-1}): 2973(*w*), 2584(*m*), 2563 (*m*), 1702(*m*), 1631(*w*), 1603(*w*), 1471(*w*), 1451(*w*), 1394(*s*), 1364(*w*), 1232(*w*), 1191(*m*), 1135(*vs*), 1088(*s*), 1048(*w*), 1022(*m*), 986(*m*), 917(*w*), 904(*m*), 878(*m*), 849 (*m*), 818(*w*), 797(*m*), 771(*m*), 759(*m*), 740(*vs*), 712(*w*), 701(*m*), 680(*m*), 658(*w*), 645(*m*), 625(*w*), 612(*w*), 573(*m*).

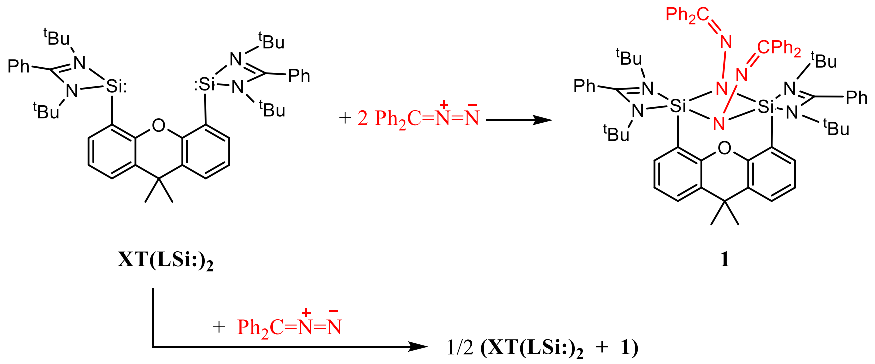

Scheme S1. Synthesis of **1**.

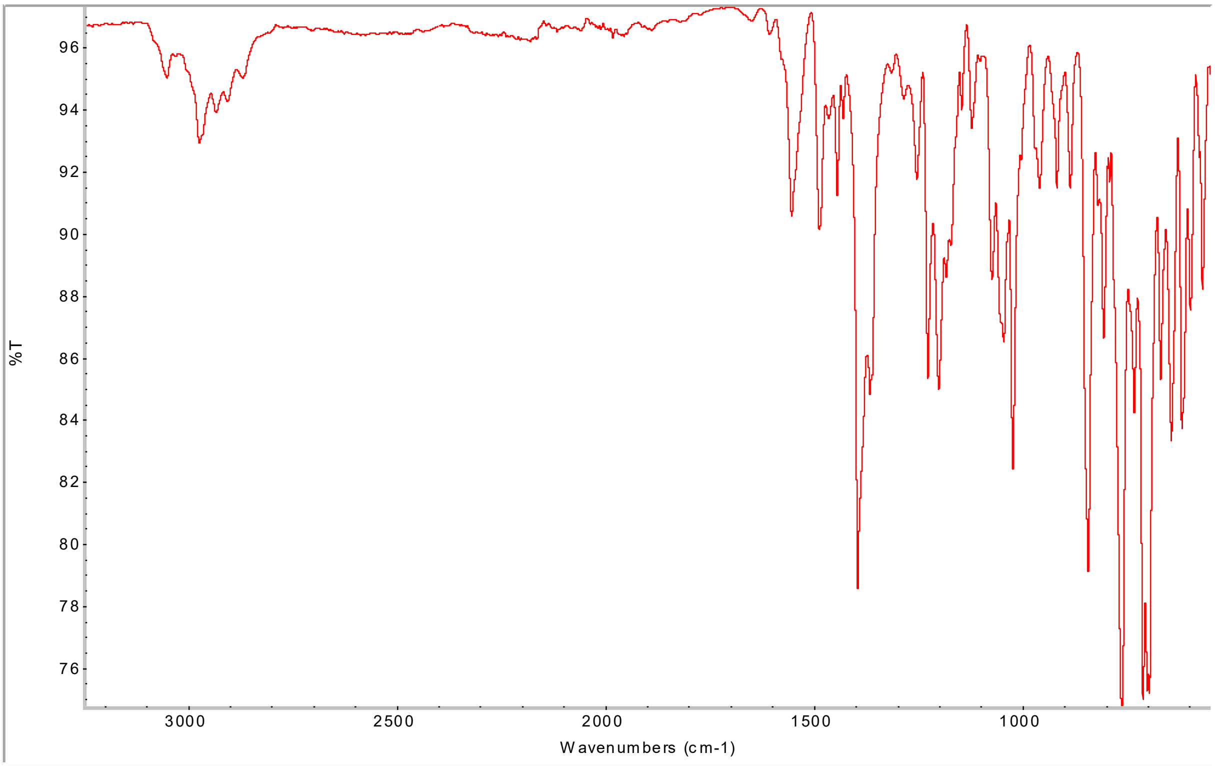

Figure S1. IR spectrum of compound **1**.

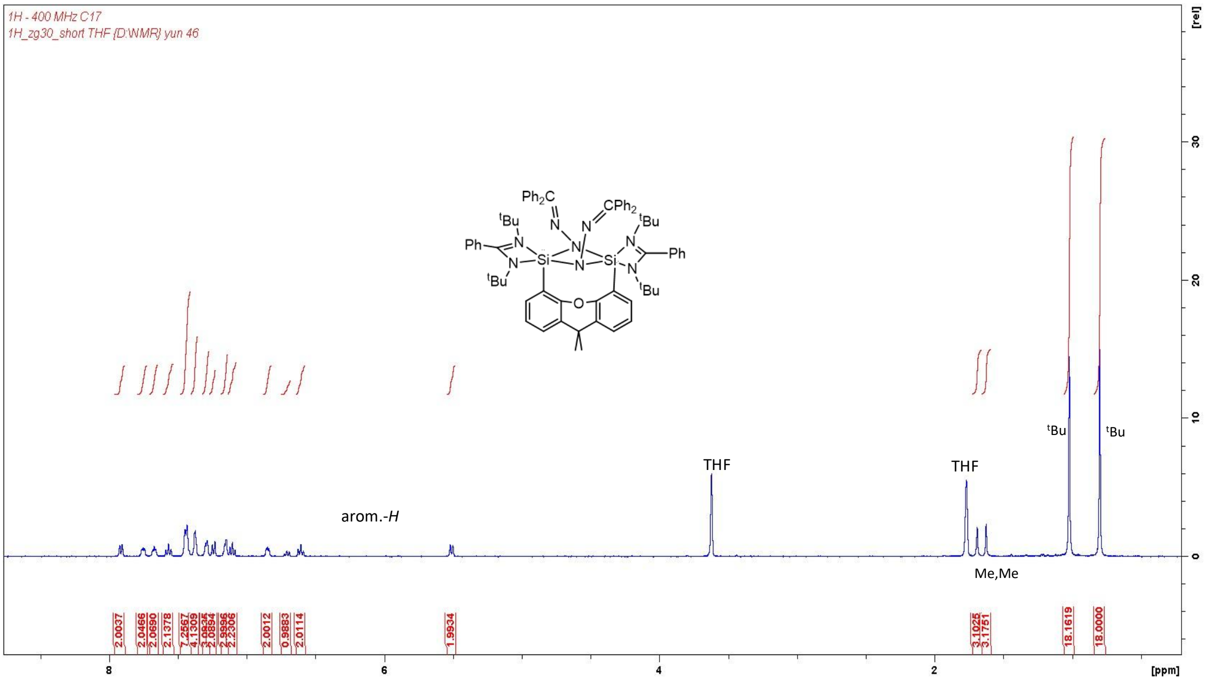

Figure S2a. ^{1}H-NMR spectrum of compound 1 (400 MHz, THF-d_8, 298K).

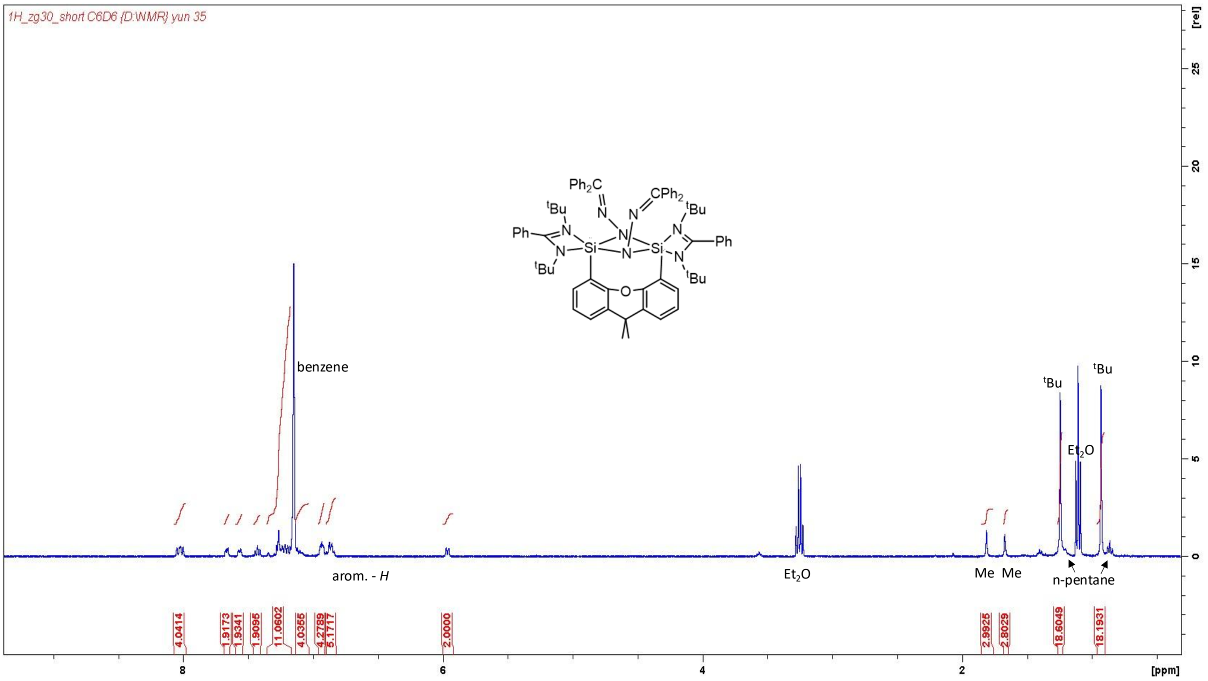

Figure S2b. ^{1}H-NMR spectrum of compound 1 (400 MHz, C_6D_6, 298K).

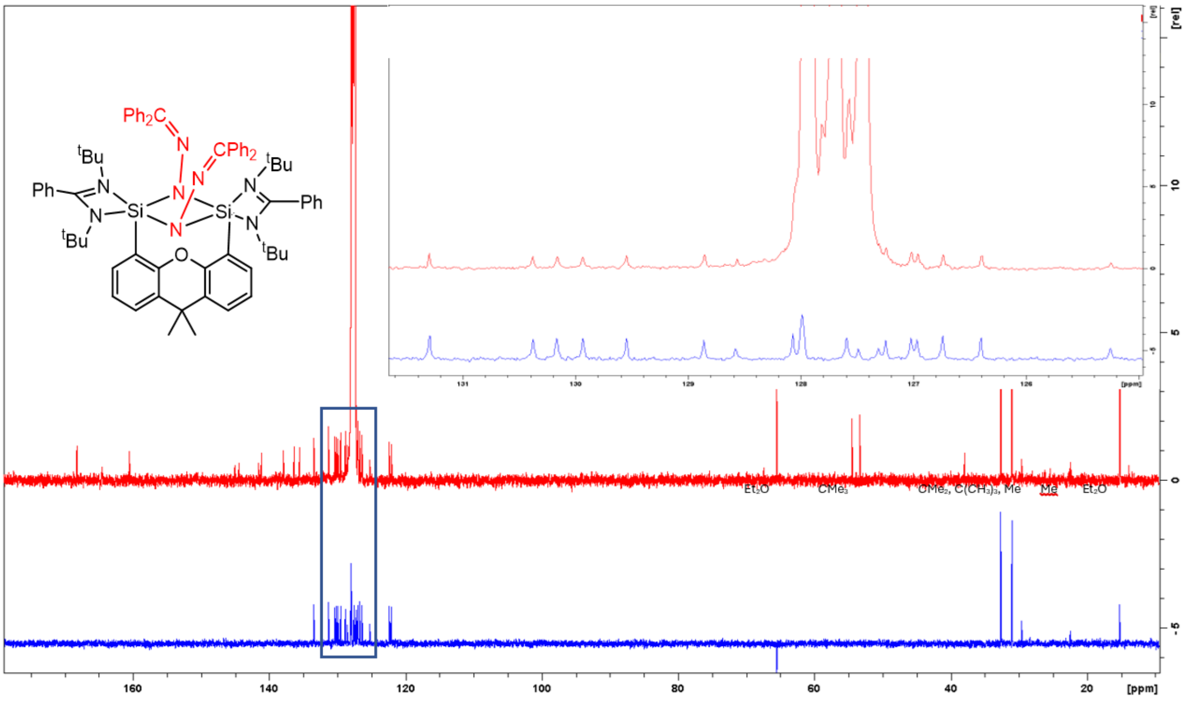

Figure S3. $^{13}C\{^1H\}$- (top) and Dept-135 (bottom)-NMR spectra of compound 1 (C_6D_6, 100 MHz, 298K)

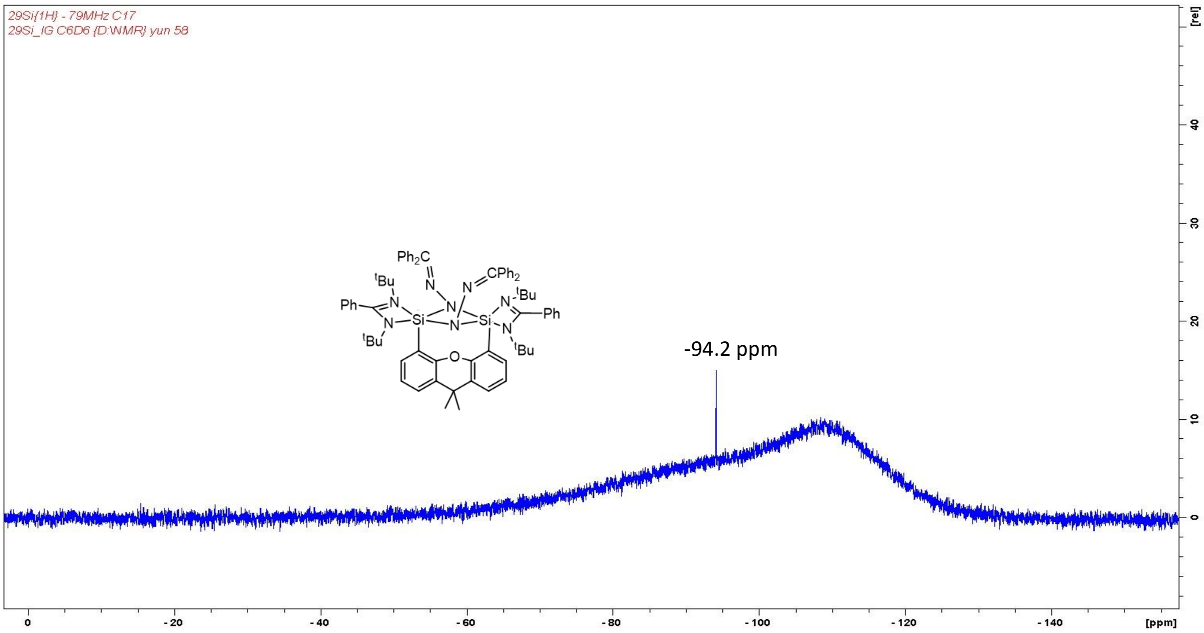

Figure S4. ^{29}Si{^{1}H}-NMR spectrum of compound 1 (79 MHz, THF-d_8, 298 K)

Compound 2: 0.26 g (0.36 mmol) of **XT(LSi:)$_2$** and 0.14 g (0.72 mmol) of 9-diazo-9*H*-florene were combined in a Schlenk flask, followed by the addition of 30 mL of Et_2O at -20 °C while stirring. The cooling bath was removed after 10 minutes. The reaction mixture was stirred overnight. Volatiles were removed under reduced pressure. The crude product was washed with *n*-pentane and dried under vacuum to afford 0.32 g (0.28 mmol, yield: 78 %) of compound **2** as orange powder. Single crystals suitable for scXRD were obtained from the *n*-pentane solution. **M.p.** 193ºC (decomp.). **^{1}H NMR** (400 MHz, THF-d_8, 298K): δ = 1.00 (*s*, 18 H, C(C*H*$_3$)$_3$), δ = 1.29 (*s*, 3 H, C*H*$_3$), 1.31 (*s*, 18 H, C(C*H*$_3$)$_3$), 1.41 (*s*, 3 H, C*H*$_3$), 6.11 (*d*, $^3J_{\text{(H, H)}}$ = 7.5 Hz, 1 H, arom.-*H*), 6.76 (*t*, $^3J_{\text{(H, H)}}$ = 7.5 Hz, 2 H, arom.-*H*), 6.91 (*t*, $^3J_{\text{(H, H)}}$ = 7.5 Hz, 2 H, arom.-*H*), 7.17 – 7.57 (*m*, 19 H, arom.-*H*), 7.83 (*t*, $^3J_{\text{(H, H)}}$ = 8.7 Hz, 4 H, arom.-*H*), 7.95 (*d*, $^3J_{\text{(H, H)}}$ = 6.3 Hz, 1 H, arom.-*H*), 8.11 (*d*, $^3J_{\text{(H, H)}}$ = 7.5 Hz, 1 H, arom.-*H*), 9.17 (*d*, $^3J_{\text{(H, H)}}$ = 8.8 Hz, 1 H, arom.-*H*), 9.49 ppm (*d*, $^3J_{\text{(H, H)}}$ = 7.5 Hz, 1 H, arom.-*H*). **^{13}C{^{1}H} NMR** (100 MHz, THF-d_8, 298K): δ = 22.4, 29.9 (C(*C*H$_3$)$_2$), 30.5, 33.4 (NC(*C*H$_3$)$_3$), 37.7 (*C*Me$_2$), 55.5, 55.8 (N*C*(CH$_3$)$_3$), 117.6, 118.4, 120.1, 120.1, 121.3, 121.8 (Ph-*C*), 122.7, 123.6, 124.4, 124.6, 125.7, 125.9, 127.3, 127.7, 127.8, 127.89, 128.0, 128.8, 129.2, 130.0, 130.1, 130.7 (Ph-*C*), 130.8 (Ph-*C*), 131.3 (*br.*), 132.2 (Ph-*C*), 132.7 (*br.*), 135.2 (Ph-*C*), 135.5 (Ph-*C*), 136.0 (Ph-*C*), 139.0 (Ph-*C*), 140.3 (Ph-*C*), 140.4 (Ph-*C*), 140.5 (Ph-*C*), 142.0 (Ph-*C*), 142.8 (Ph-*C*), 158.9 (N*C*N), 160.3 (N*C*N), 171.9 ppm (*C*Ph$_2$). **^{29}Si-MAS NMR** (10kHz, 298 K): δ = -91.8 (*s*, Si), -105.3 ppm (*s*, *Si*). **HR-ESI-MS: m/z**: 1111.5557 (*calc*. 1111.5552 [M]$^+$, m/z); **IR** (cm^{-1}): 2959(*w*), 1569(*w*), 1526(m), 1445(*w*), 1388(*m*), 1361(*w*), 1334(*w*), 1311(*w*), 1226(*vs*), 1205(*s*), 1152(s), 1098(m), 1074 (*w*), 1045 (*m*), 1029 (*m*), 984 (*w*), 957 (*w*), 946 (w), 837 (*m*), 819 (w), 808 (*m*), 798 (*w*), 765 (*s*), 744 (w), 729 (s), 720 (m), 705 (m), 693 (m), 648 (w), 623 (s), 598 (*m*).

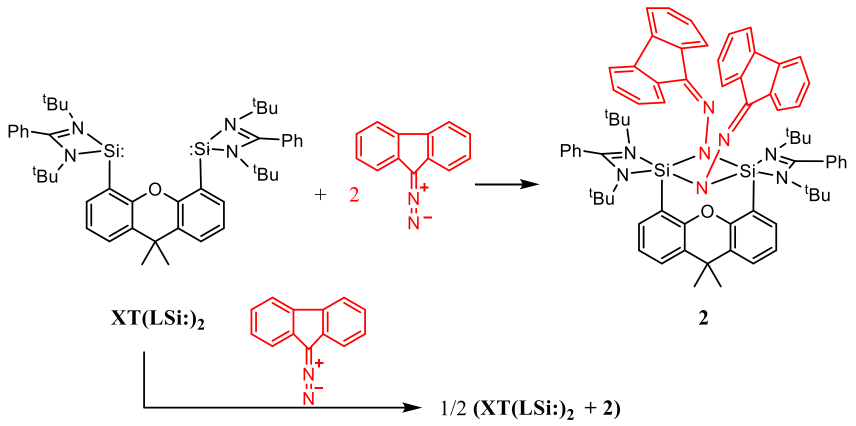

Scheme S2. Synthesis of **2**.

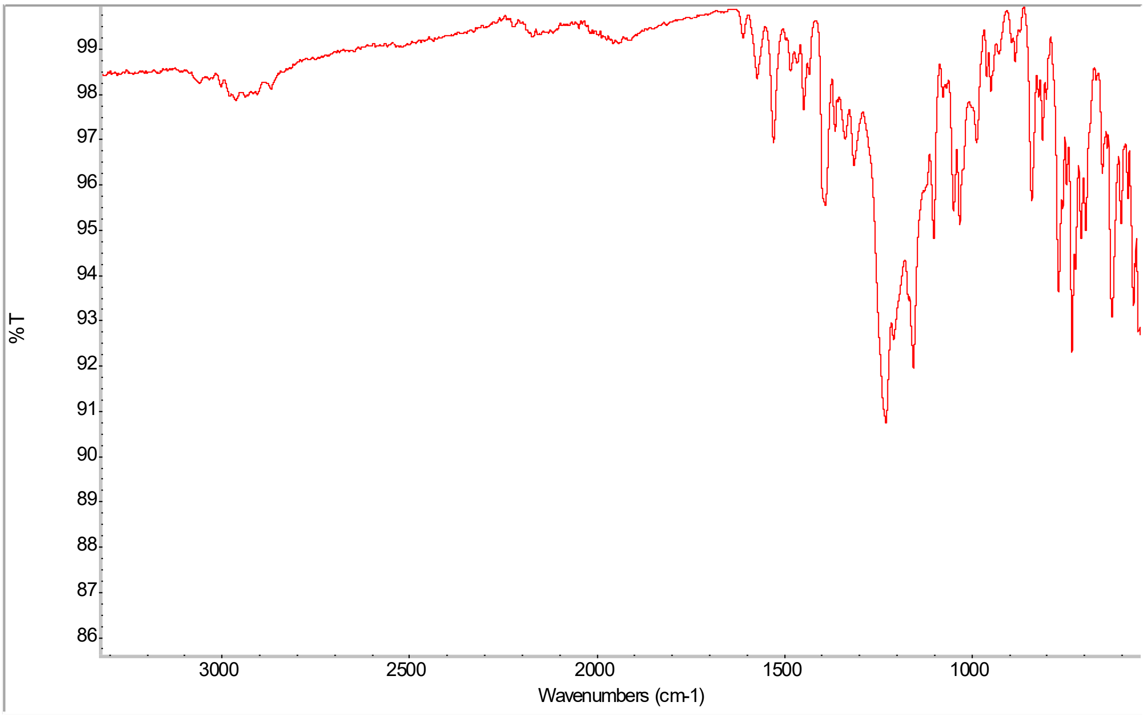

Figure S5. IR spectrum of compound **2**.

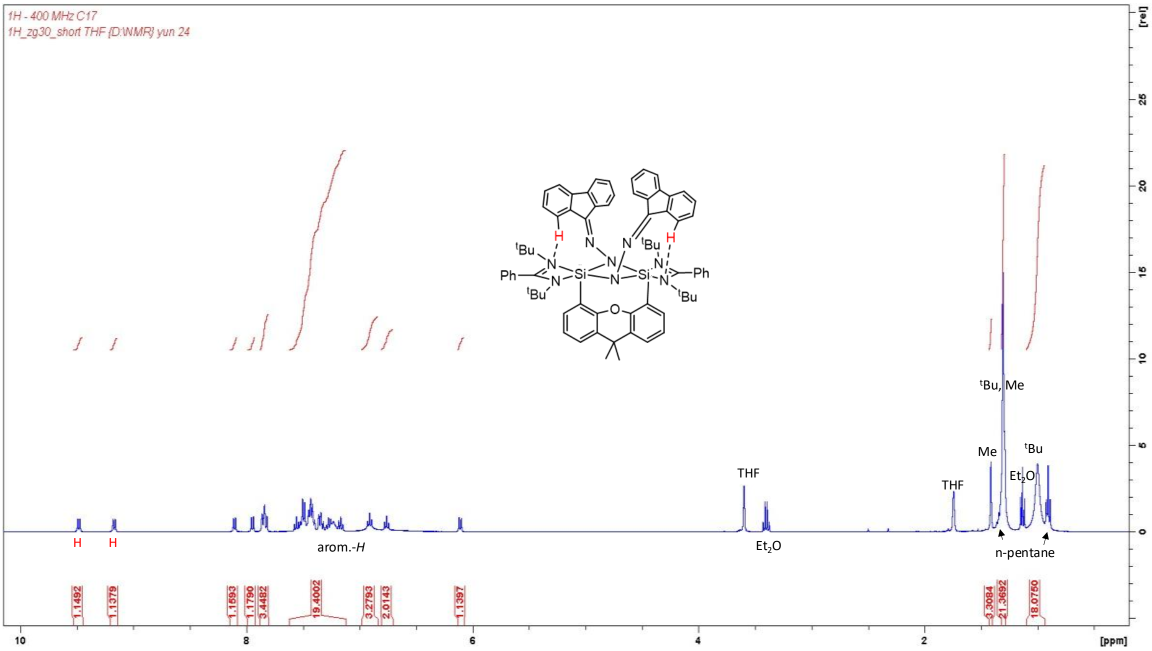

Figure S6. ^{1}H-NMR spectrum of compound 2 (400 MHz, THF-d_8, 298K). The two strongly downfield-shifted ^{1}H NMR signals at δ = 9.49 and 9.17 ppm originate from the protons at the 1 and 8 positions of the Ar_2C=N=N (Ar_2C = 9,9-fluorenyl) moieties. In the single-crystal X-ray structure, these protons exhibit the closest H···N contacts (2.307 Å and 2.575 Å), explaining their pronounced deshielding. This assignment is further supported by GIAO calculations, which predict two strongly downfield-shifted signals at δ = 10.60 and 10.45 ppm on the level of PBE0 D4/def2-TZVPP CPCM(THF). The calculated values are approximately 1 ppm more downfield than the experimental signals, consistent with known limitations of GIAO predictions.

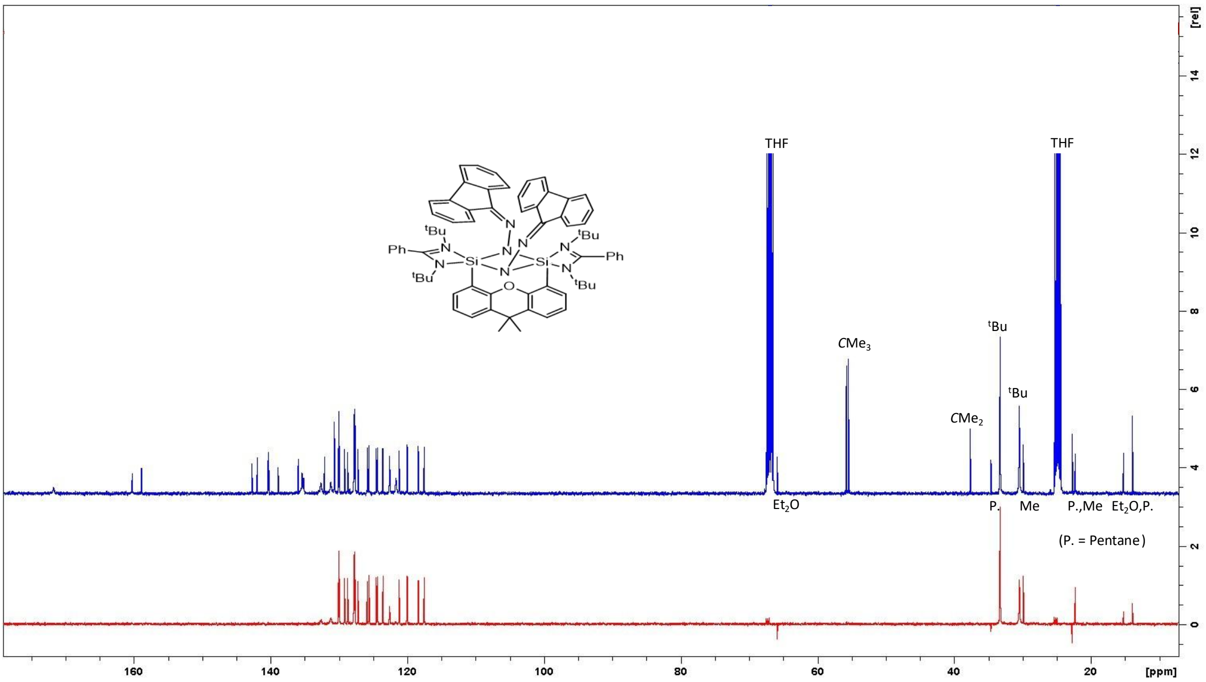

Figure S7. ^{13}C{^{1}H}- (top) and Dept-135 (bottom)-NMR spectra of compound 2 (THF-d_8, 100 MHz, 298K)

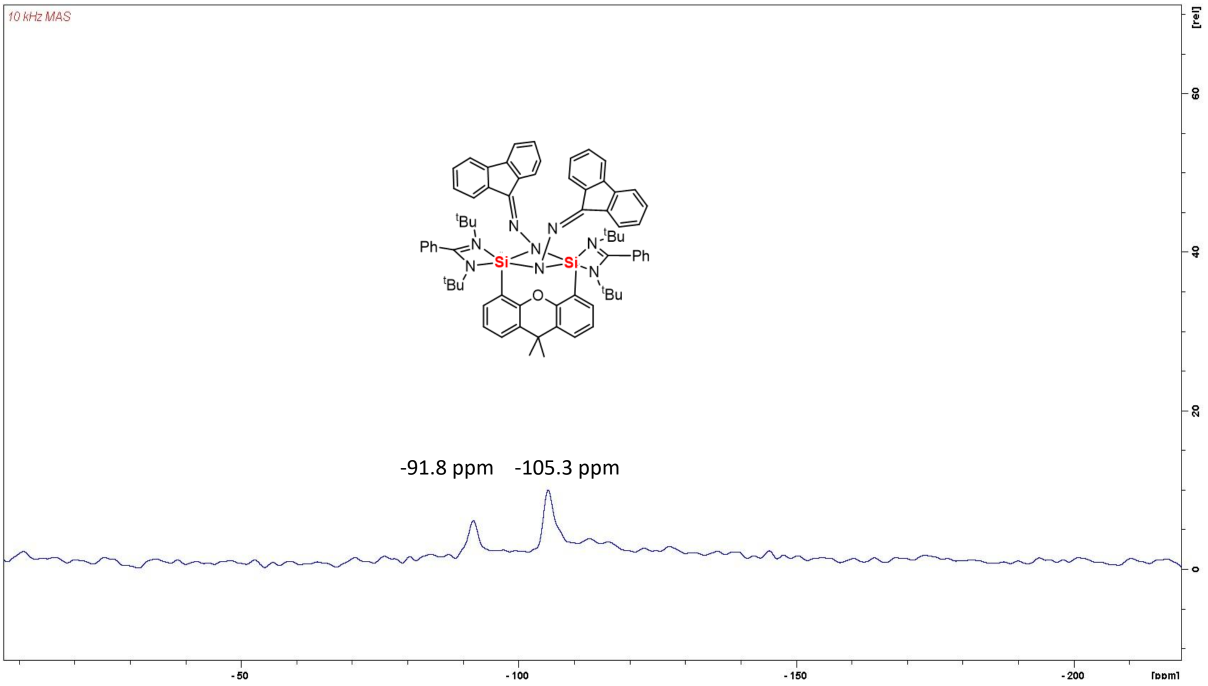

Figure S8. ^{29}Si-MAS NMR spectrum of compound 2 (10kHz, 298 K)

Compound 3: To a Schlenk flask charged with **PhN(LSi:)$_2$** (0.30 g, 0.49 mmol) and Ph_2CN_2 (0.19 g, 0.98 mmol) was added 20 mL Et_2O at -20 °C with stirring. Orange precipitate can be observed immediately. The reaction mixture was stirred overnight at room temperature and the orange precipitate was separated by filtration and washed with Et_2O (3×5 mL). It afforded 0.20 g yellow powder after dried under vacuum. The single crystals of **3** qualified for X-ray diffraction analysis were obtained by recrystallisation of the yellow powder in toluene at 4 °C. The NMR spectroscopy showed that the crystals were a mixture of **3** and some unidentified species. Attempts to isolate compound **3** in its pure form for thorough characterization were unsuccessful due to the presence of unidentified species. **HR-ESI-MS**: m/z: 998.5448 (calc. 998.5444 [M]+, m/z) for **3**. It should be noted that the equimolar reaction of PhN(LSi:)$_2$ and Ph_2CN_2 produced also compound **3**.

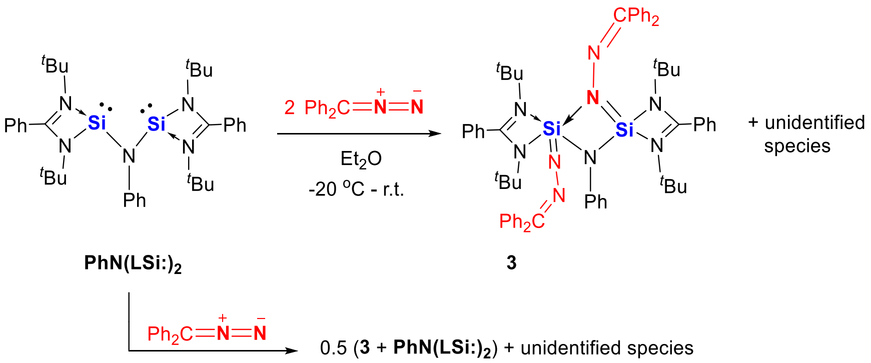

Scheme S3. Formation of **3**.

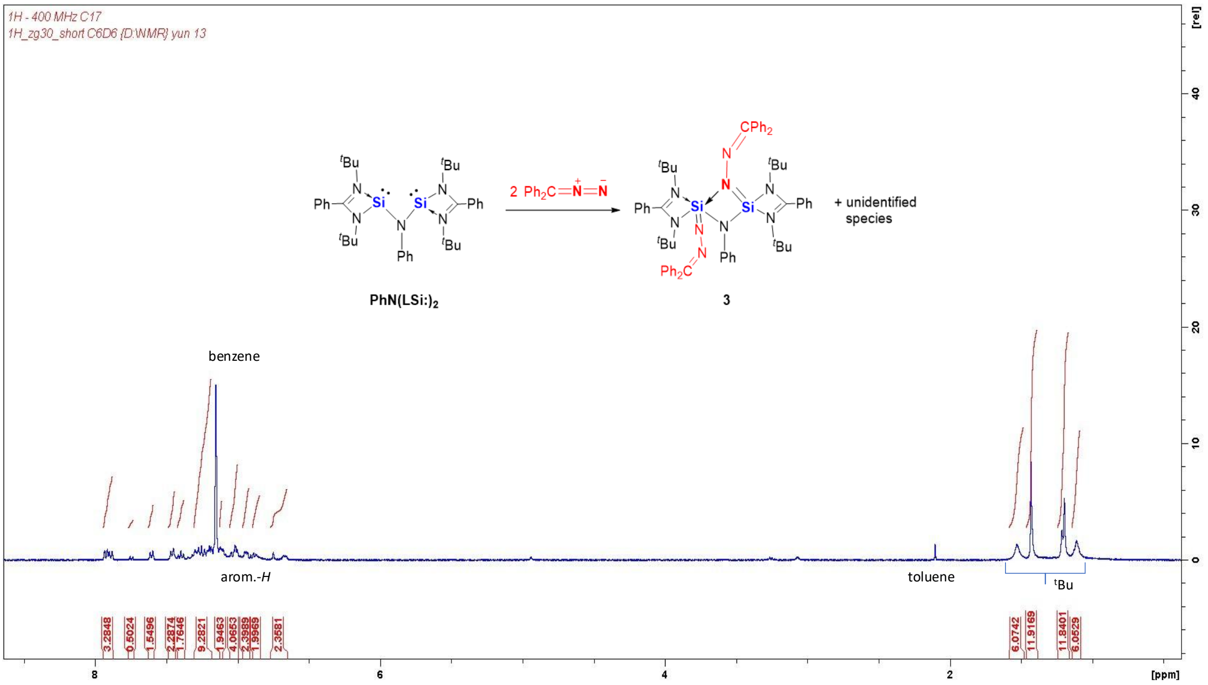

Figure S9a. ^{1}H NMR Spectrum of **3** with unidentified species (500 MHz, C_6D_6, 298K).

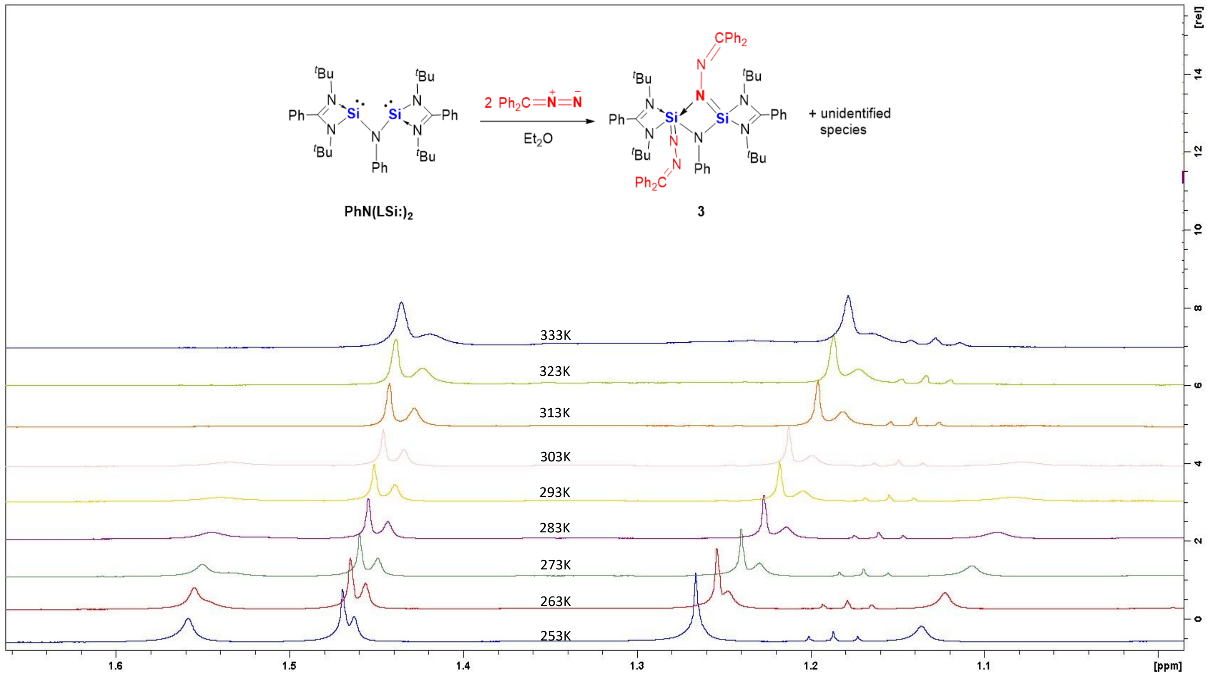

Figure S9b. ^{1}H NMR Spectra of **3** with unidentified species (only the 1.02–1.62 ppm region was shown) (VT, 500 MHz, toluene-d_8).

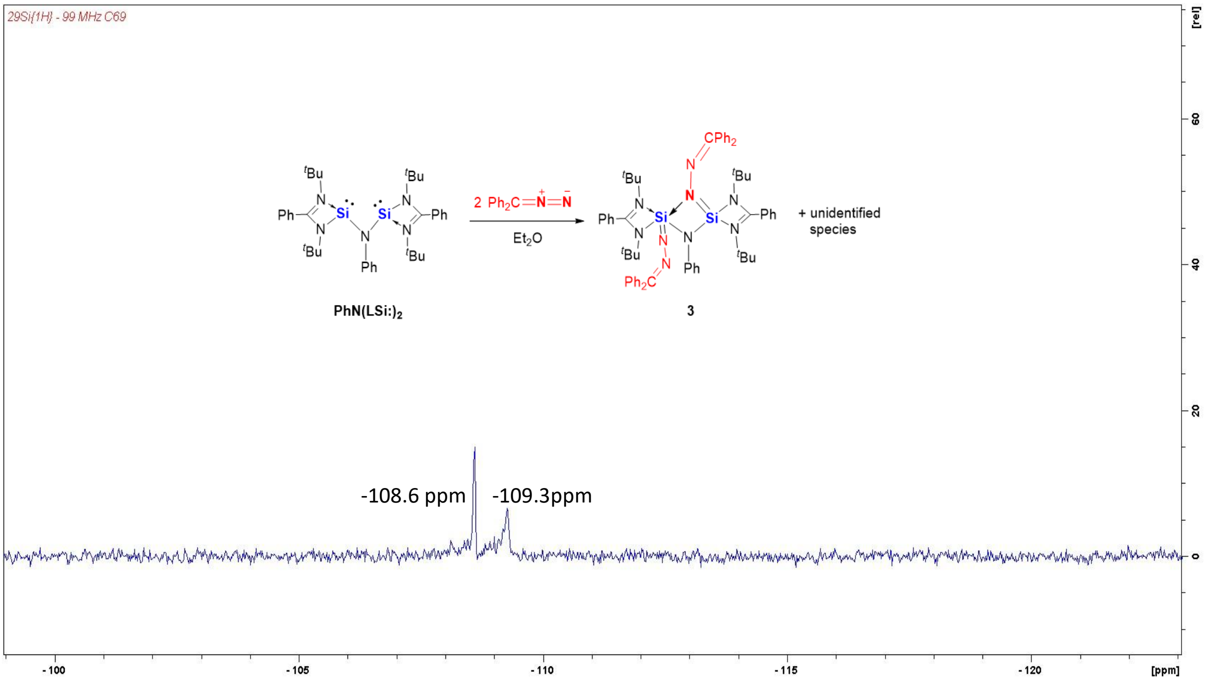

Figure S9c. ^{29}Si{^{1}H} NMR Spectrum of **3** with unidentified species (99 MHz, C_6D_6, 298K).

Compound 4: To a Schlenk flask charged with **CB(LSi:)$_2$** (0.41 g, 0.62 mmol) and Ph_2CN_2 (0.12 g, 0.62 mmol) was added 20 mL Et_2O at -40 °C with stirring. Yellow precipitate can be observed after 10 minutes. The cool bath was then removed and the reaction mixture was stirred overnight. The yellow precipitate of **4** was separated by filtration and washed with 5 mL Et_2O. It afforded 0.32 g (0.37 mmol, yield: 60 %) of **4** after dried under vacuum. The single crystals of **4** qualified for X-ray diffraction analysis were obtained by recrystallisation of the yellow solid in toluene at room temperature. **M.p.** 210°C (decomp.). **^{1}H NMR** (400 MHz, C_6D_6, 298K): δ = 0.65 (*br*, 9 H, C(C*H*$_3$)$_3$), 0.91 (*br*, 9 H, C(C*H*$_3$)$_3$), δ = 1.02 (*br*, 9 H, C(C*H*$_3$)$_3$), 2.11 (*br*, 9 H, C(C*H*$_3$)$_3$), 6.49 (*d*, $^{3}J_{\text{(H, H)}}$ = 7.6 Hz, 1 H, arom.-*H*), 6.60 – 6.24 (*m*, 2 H, arom.-*H*), 6.69 (*t*, $^{3}J_{\text{(H, H)}}$ = 7.2 Hz, 1 H, arom.-*H*), 6.75 – 6.79 (*m*, 1 H, arom.-*H*), 7.04 – 7.21 (*m*, 5 H, arom.-*H*), 7.24 – 7.28 (*m*, 6 H, arom.-*H*), 8.10 ppm (*d*, $^{3}J_{\text{(H, H)}}$ = 6.2 Hz, 4 H, arom.-*H*). **^{11}B NMR** (128 MHz, THF-*d*$_6$, 298K): δ = -10.0 ppm (*br*, 6 B), -3.0 ppm (*br,* 4 B). **^{13}C{^{1}H} NMR** (100 MHz, C_6D_6, 298K): δ = 31.2, 31.2, 31.9, 33.2 (NC(*C*H$_3$)$_3$), 54.7, 55.0, 55.4, 57.8 (N*C*(CH$_3$)$_3$), 74.2 (*C*$_{carb.}$), 86.9 (*C*$_{carb.}$), 126.5, 127.2, 127.3, 127.4, 127.8, 127.9, 128.0, 128.9 (Ph-*C*), 130.6, 131.0, 140.7 (Ph-*C*), 141.4 (Ph-*C*), 160.5 (N*C*N), 170.8 (N*C*N), 181.9 ppm (*C*Ph$_2$). **^{29}Si{^{1}H} NMR** (79 MHz, C_6D_6, 298 K): δ = -29.3 (*s*, *Si*=N), -41.4 ppm (*s*, *Si*). **HR-ESI-MS: m/z**: 855.5962 (*calc*. 855.5963 [M+H]$^{+}$); **IR** (cm^{-1}): 2972 (*w*), 2547 (*w*), 1649 (*m*), 1577 (*w*), 1444 (*w*), 1395 (*s*), 1369 (*m*), 1357 (*w*), 1247 (*m*), 1176 (*m*), 1136 (*vs*), 1086 (*s*), 1044 (*w*), 1023 (*m*), 979 (*m*), 907 (*w*), 876 (*m*), 844 (*m*), 808 (*m*), 793 (*m*), 775 (*m*), 758 (*m*), 730 (*w*), 695 (*vs*), 677 (*s*), 644.61(*s*), 631 (*m*), 624 (*m*), 581 (*w*).

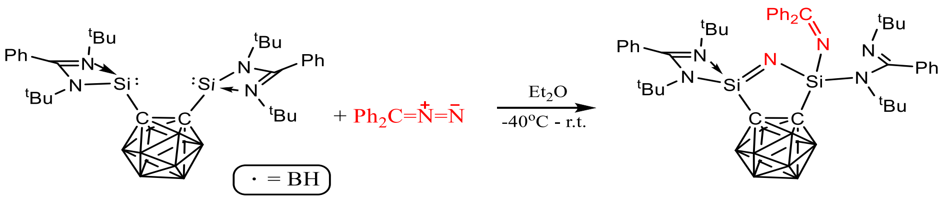

Scheme S4. Synthesis of **4**.

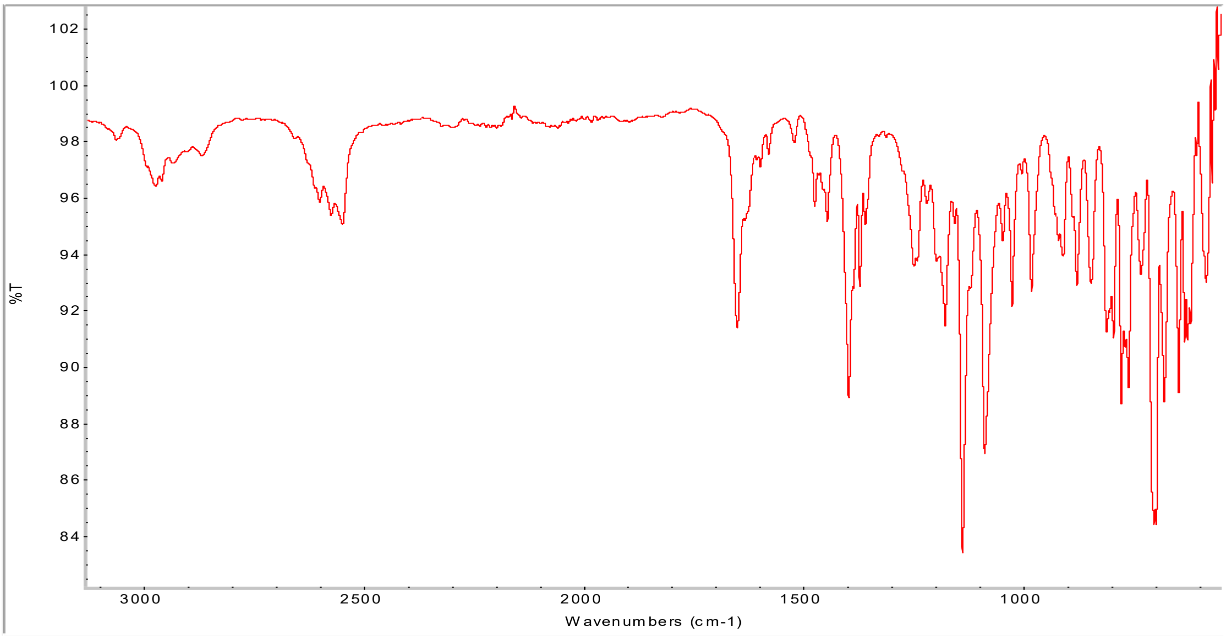

Figure S10. IR spectrum of compound 4.

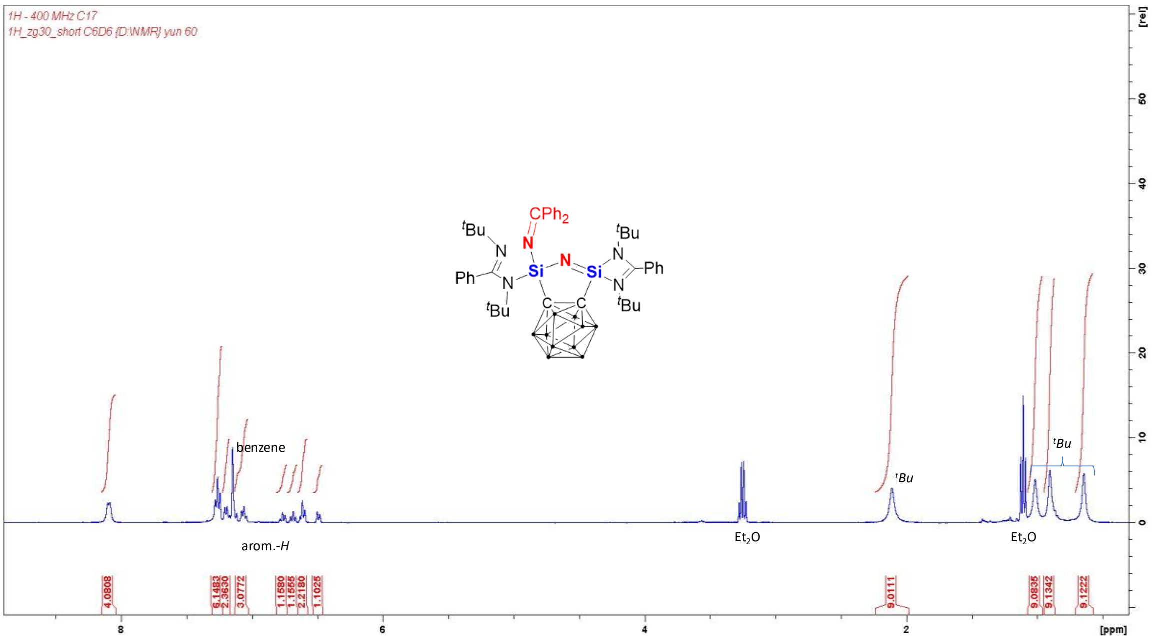

Figure S11. ^{1}H-NMR spectrum of compound 4 (400 MHz, C_6D_6, 298K).

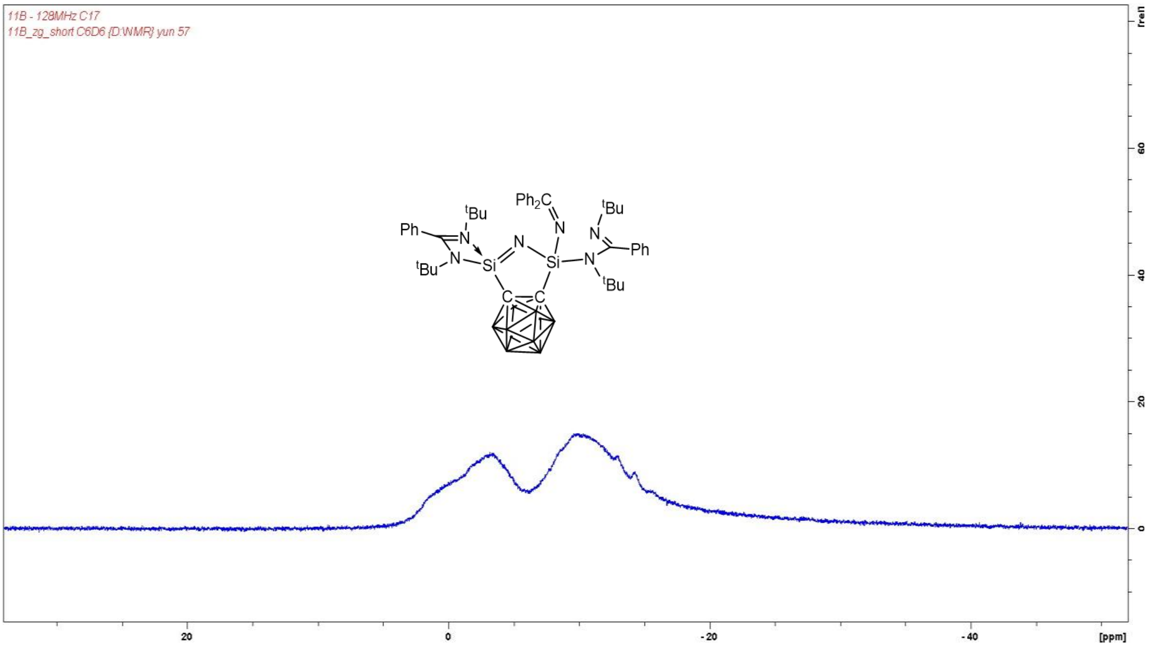

Figure S12. ^{11}B-NMR spectrum of compound 4 (400 MHz, C_6D_6, 298K).

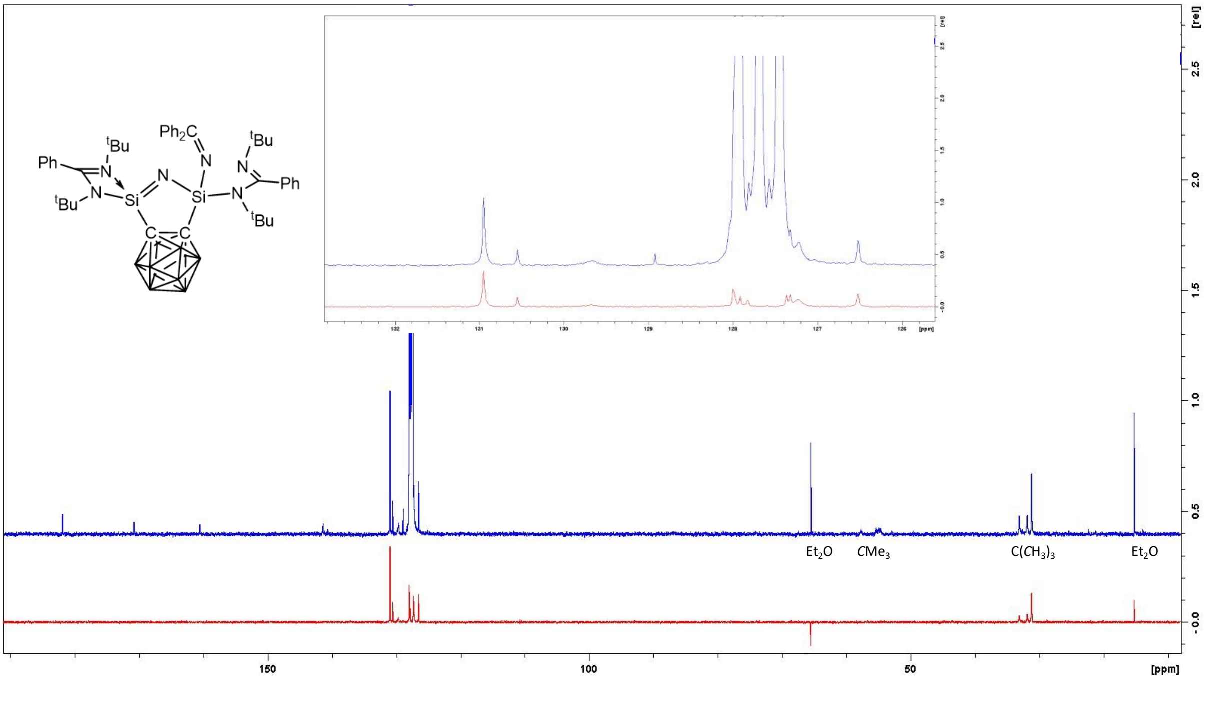

Figure S13. ^{13}C{^{1}H}- (top) and Dept-135 (bottom)-NMR spectra of compound 4 (C_6D_6, 100 MHz, 298K)

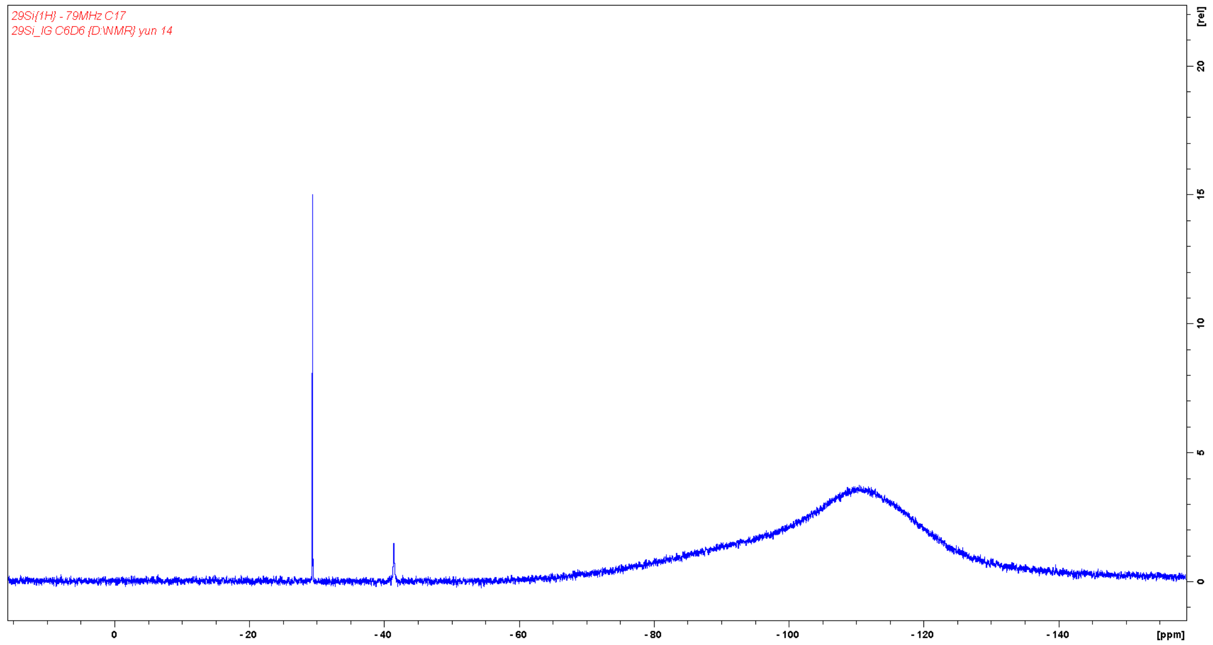

Figure S14. ^{29}Si{^{1}H}-NMR spectrum of compound 4 (79 MHz, THF-D$_6$, 298 K)

Compound 5: To a yellow solution of **CB(LSi:)$_2$** (0.55 g, 0.83 mmol) in Et$_2$O (50 mL) was added a red solution of 9-diazo-9*H*-florene (0.16 g, 0.83 mmol) in Et$_2$O (15 mL) at -20 °C. The reaction mixture turned to brown immediately. The cool bath was removed and the clear reaction mixture was stored overnight at room temperature. After one-night orange-yellow crystals of **5** were formed which were separated by filtration. It afforded 0.39 g (0.46 mmol, yield: 55 %) orange crystals of **5** after dried under vacuum. **M.p.** 220°C (decomp.). **^{1}H NMR** (400 MHz, THF-d_8, 298K): δ = 0.66 (*br*, 9 H, C(CH_3)$_3$), 0.84 (*br*, 9 H, C(CH_3)$_3$), δ = 1.30 (*br*, 9 H, C(CH_3)$_3$), 1.75 (*br*, 9 H, C(CH_3)$_3$), 1.95 – 3.16 (*br.*, 10 H, B*H*), 7.13 – 7.21 (*m*, 3 H, arom. -*H*), 7.27 – 7.34 (*m*, 5 H, arom. -*H*), 7.40 (*t*, $^{3}J_{\text{(H, H)}}$ = 7.4 Hz, 2 H, arom. -*H*), 7.54 – 7.64 (*m*, 5 H, arom. -*H*), 7.74 (*d*, $^{3}J_{\text{(H, H)}}$ = 8.0 Hz, 1 H, arom. -*H*), 7.86 – 8.40 ppm (*br.*, 2 H, arom.-*H*). **^{11}B NMR** (128 MHz, THF-d_8, 298K): δ = -10.09 ppm (*br.*, 6 B), -6.60 – 2.30 ppm (*m*, 4 B). **^{13}C{^{1}H} NMR** (100 MHz, THF-d_8, 298K): δ = 30.6 – 32.1 (*br.*, NC(*C*H$_3$)$_3$), 55.3 – 55.8 (*br.*, N*C*(CH$_3$)$_3$), 74.4 ($C_{carb.}$), 86.0 ($C_{carb.}$), 119.0, 126.2, 127.5, 127.6, 128.2, 128.3, 128.4, 128.5, 129.2 (Ph-*C*), 130.9, 131.2, 131.4, 140.9 (*br.*, Ph-*C*), 143.4 (Ph-*C*), 158.8 (N*C*N), 168.1 (N*C*N), 182.9 ppm (*C*Ph$_2$). **^{29}Si{^{1}H} NMR** (79 MHz, THF-d_8, 298 K): δ = -29.0 (*s*, *Si*=N), -41.0 ppm (*Si*). **HR-ESI-MS: m/z**: 853.5765 (*calc.* 853.5807 [M+H]$^+$, m/z); **IR** (cm^{-1}): 2973 (*w*), 2594 (*m*), 2563 (*m*), 1702 (*m*), 1631 (*w*), 1603 (w), 1471 (w), 1451 (*w*), 1394 (*s*), 1366 (*m*), 1232 (*w*), 1191 (*s*), 1135 (*vs*), 1088 (*s*), 1048 (*w*), 1022 (*m*), 986 (*m*), 917 (*w*), 904 (m), 878 (m), 849 (m), 818 (w), 797 (*m*), 771 (*m*), 759.28(*m*), 740 (*vs*), 712 (w), 701 (m), 680 (*m*), 658 (*w*), 645 (*m*), 625 (*w*), 612 (*w*), 573 (*w*).

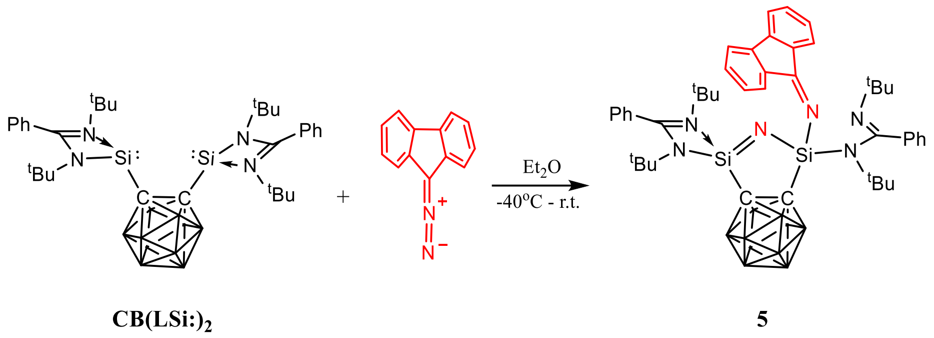

Scheme S5. Synthesis of **5**.

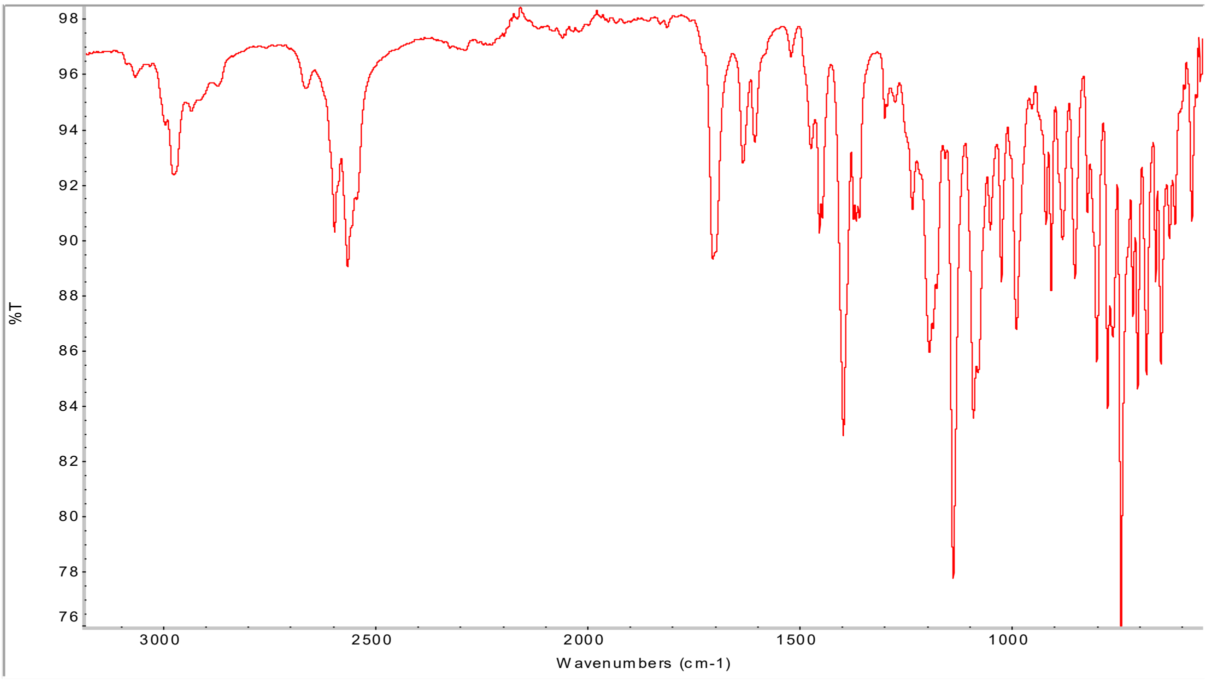

Figure S15. IR spectrum of compound 5.

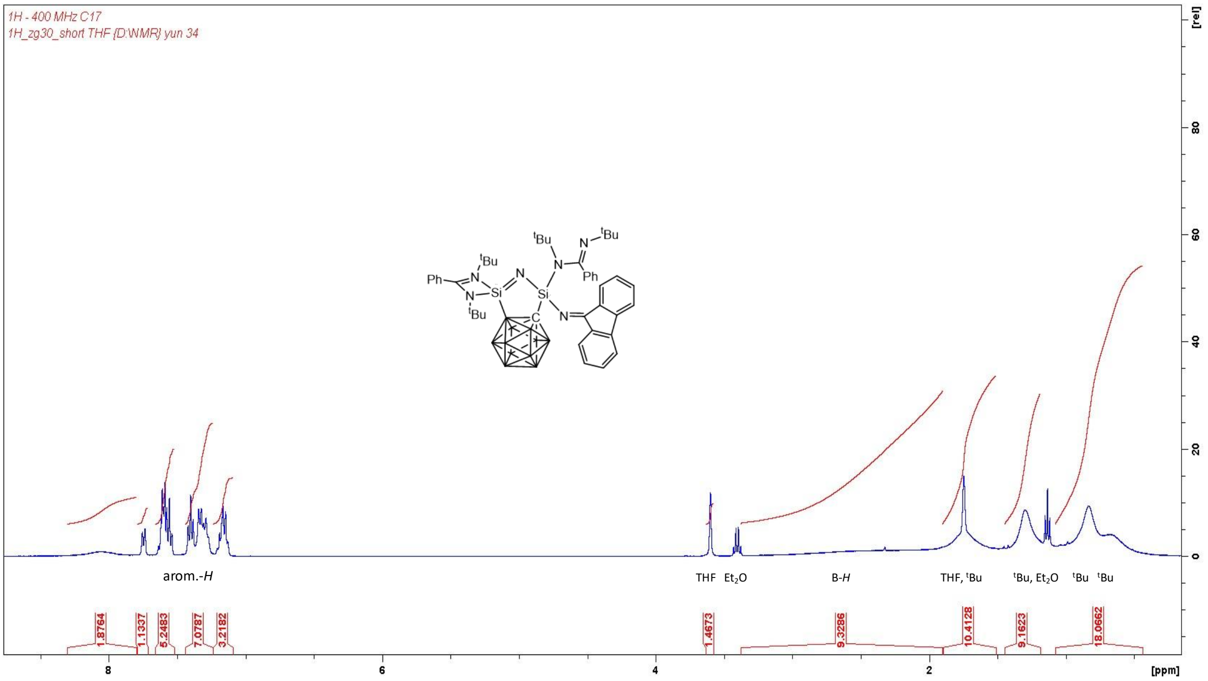

Figure S16. ^{1}H-NMR spectrum of compound 5 (400 MHz, THF-d_8, 298K).

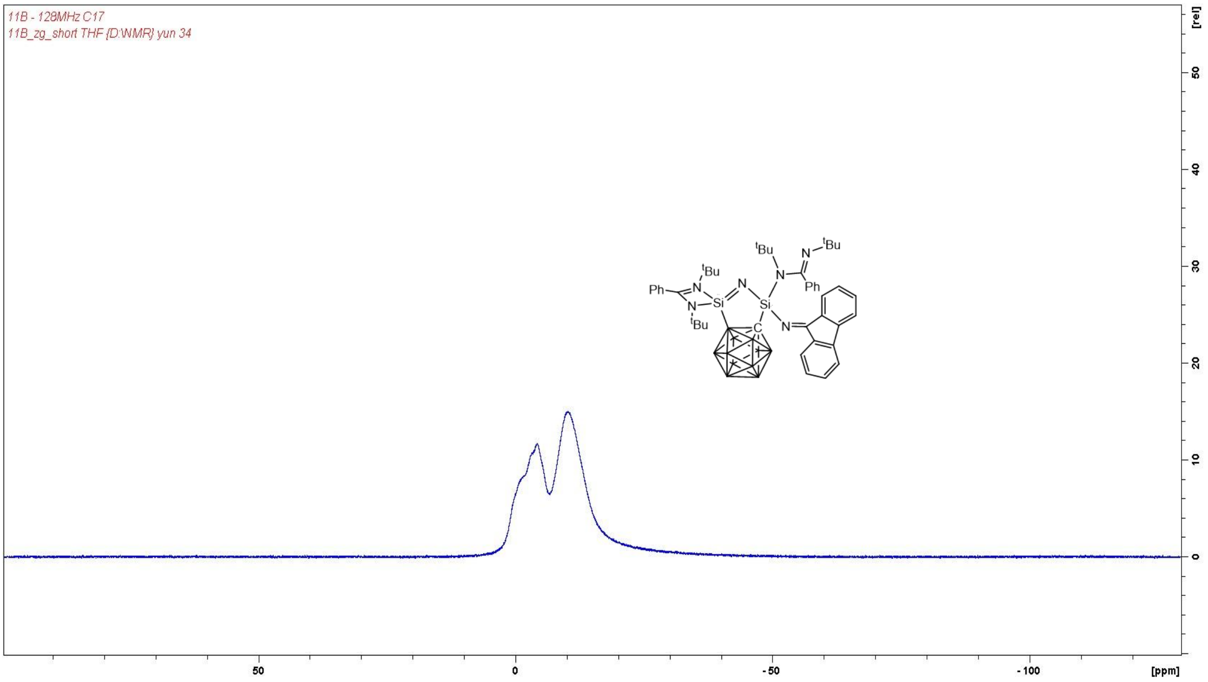

Figure S17. ^{11}B{^{1}H}-NMR spectrum of compound 5 (THF-d_8, 128 MHz, 298K)

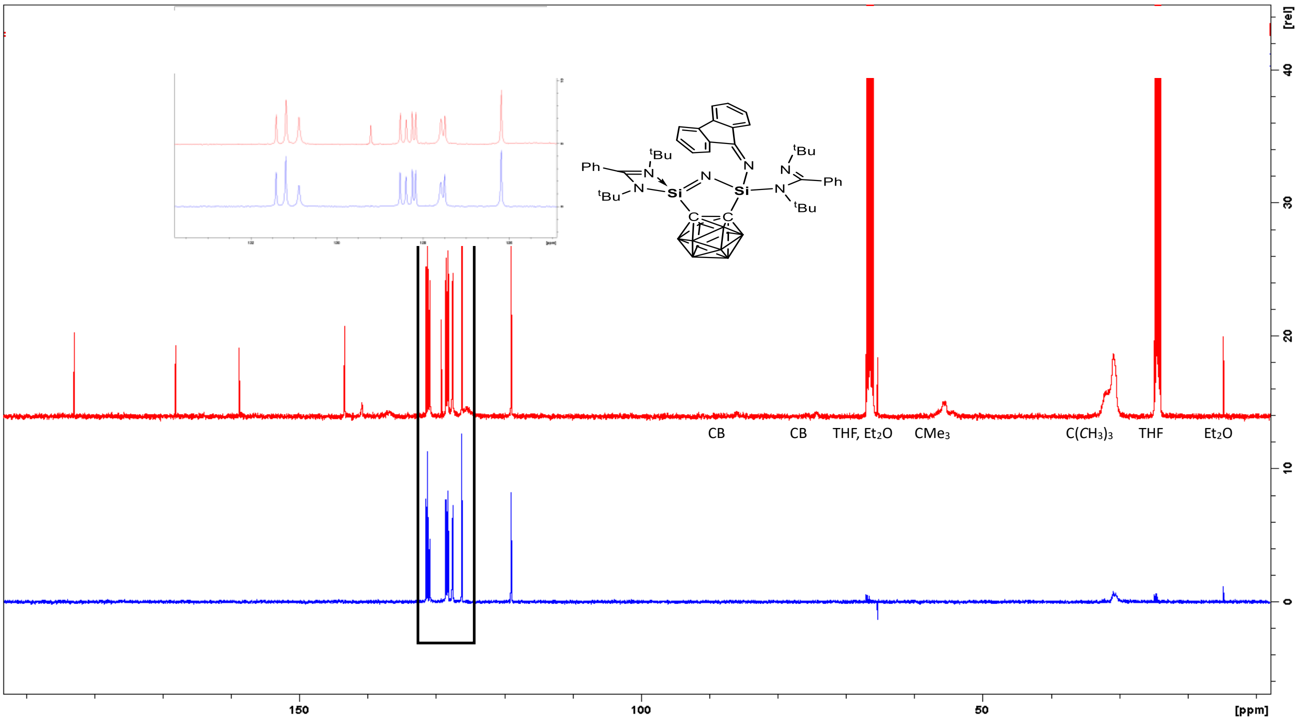

Figure S18. $^{13}C\{^1H\}$- (top) and Dept-135 (bottom)-NMR spectra of compound 5 (THF-d_8, 100 MHz, 298K)

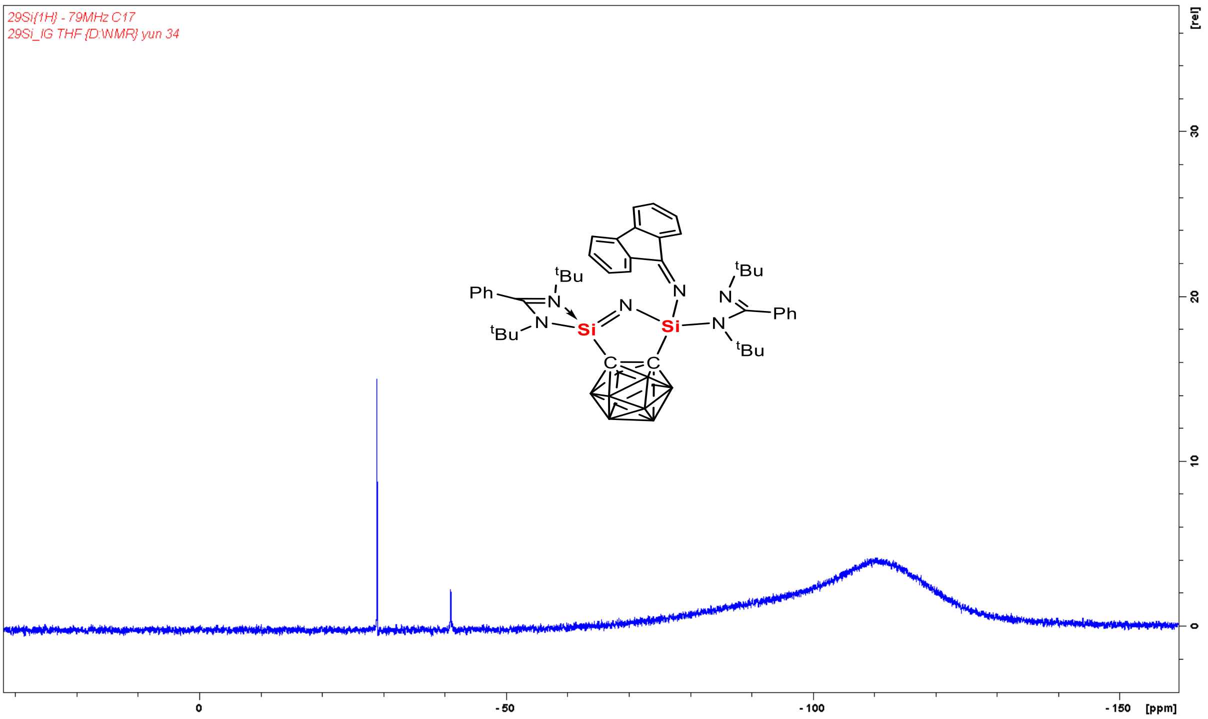

Figure S19. $^{29}Si\{^1H\}$-NMR spectrum of compound 5 (79 MHz, THF-d_8, 298 K)

Reaction of compound 5 with H_2O: The Schlenk flask containing **5** (0.21 g, 0.24 mmol) and toluene (30 mL) was connected to another Schlenk flask containing toluene (10 mL) and water (5 drops) at room temperature. After one day, the orange-yellow color of **5** disappeared, resulting in a colorless colloid. ESI-MS spectroscopy confirmed the formation of 9*H*-fluorene-9-imine ($180.0803_{exp.}$, $180.0808_{calc.}$), amidine **L**H ($233.2008_{exp.}$, $233.2012_{calc.}$), and 1,2-dicarborane ($145.2027_{exp.}$, $145.2015_{cacl.}$) (Scheme S6). Sublimation at 60 °C and 1 × 10^{-3} mbar yielded colorless crystals of **L**H and 1,2-dicarborane, which were confirmed by ^{1}H and ^{11}B{^{1}H} NMR spectroscopy (Figures S20 and S21) and X-ray diffraction analysis. Subsequent sublimation at 80 °C and 1 × 10^{-3} mbar afforded yellowish crystalline 9*H*-fluorene-9-imine (^{1}H NMR spectrum in Figure S20). SiO_2 remained in the residue. The produced NH_3 could be found in the sealed nmr tube reaction (Figure S22).

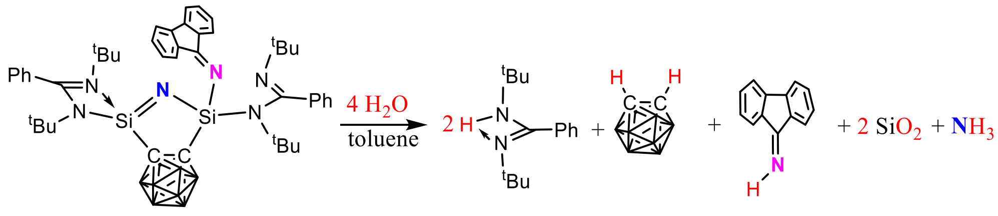

Scheme S6. Reaction of **5** with H_2O.

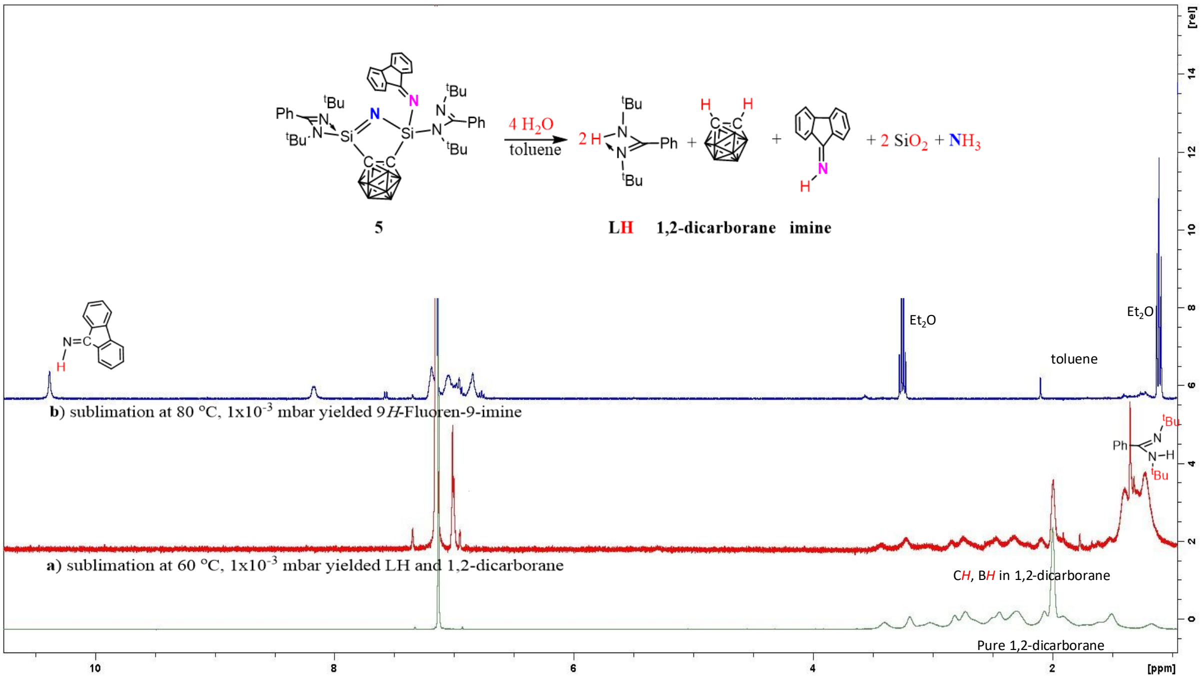

Figure S20. ^{1}H NMR spectra of the products obtained via stepwise sublimation from the reaction of **5** with water: **a**) **L**H with 1,2-dicarborane, **b**) 9*H*-fluorene-9-imine (400 MHz, C_6D_6, 298K). The ^{1}H NMR spectrum of pure 1,2-dicarborane is attached for comparison (bottom).

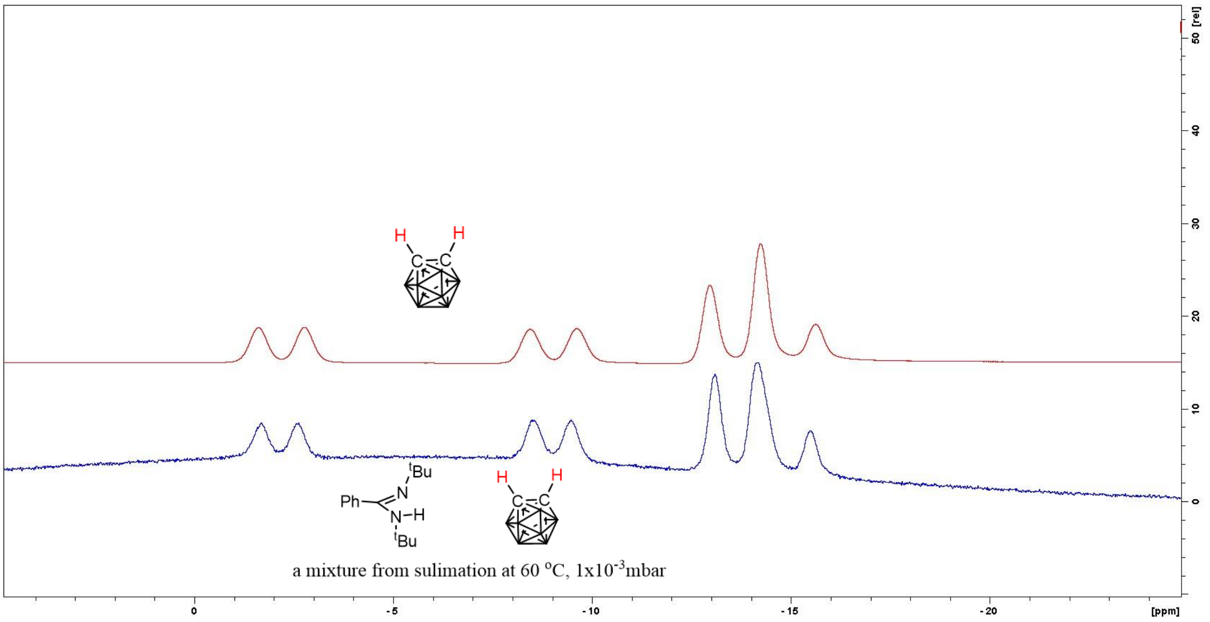

Figure S21. $^{11}B\{^{1}H\}$ NMR spectra of the pure 1,2-dicarborane (top) and the products (**L**H and 1,2-dicarborane) via sublimation at 60 ºC and $1x10^{-3}$ mbar (bottom) (128 MHz, C_6D_6, 298K).

Identification of NH_3 from the reaction of compound 5 with H_2O in a sealed NMR tube: 5 μL of water was added to an NMR tube containing **5** (0.0050 g) and 0.50 mL of C_6D_6 under a nitrogen atmosphere at room temperature. After 16 hours, the reaction was complete. The proton NMR spectrum indicated that, in addition to the formation of the corresponding 9*H*-fluorene-9-imine, **L**H, and 1,2-dicarborane, ammonia was also present, resulting in a 1:1:1 triplet at δ -0.19 ppm with a coupling constant of $^{1}J_{(14N\text{-}1H)} = 43$ Hz (Figure S22).

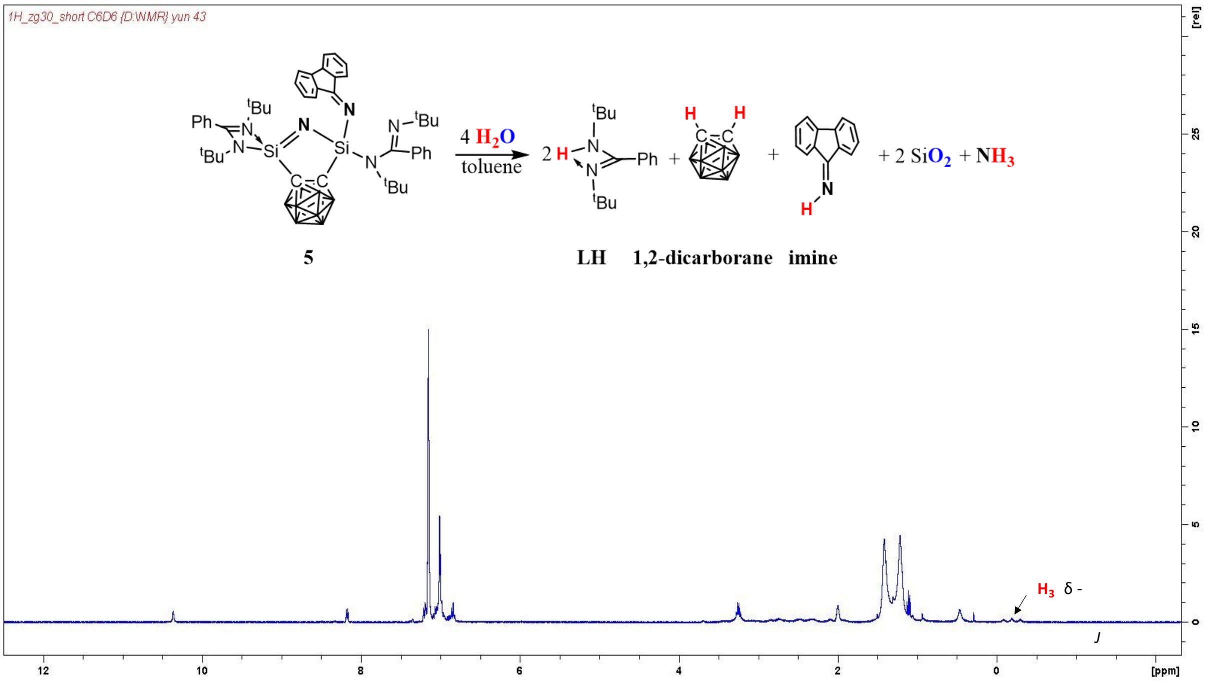

Figure S22. ^{1}H NMR spectrum of the reaction solution of **5** and H_2O in a sealed NMR tube (400 MHz, C_6D_6, 298 K)

To further identify the reaction products, the reaction of **5** with D_2O in a sealed NMR tube was conducted. In the products the corresponding signals for D atoms from D_2O disappeared in the ^1H NMR spectrum (Figures S23), however, they appeared in the ^2H NMR spectrum (Figure S24).

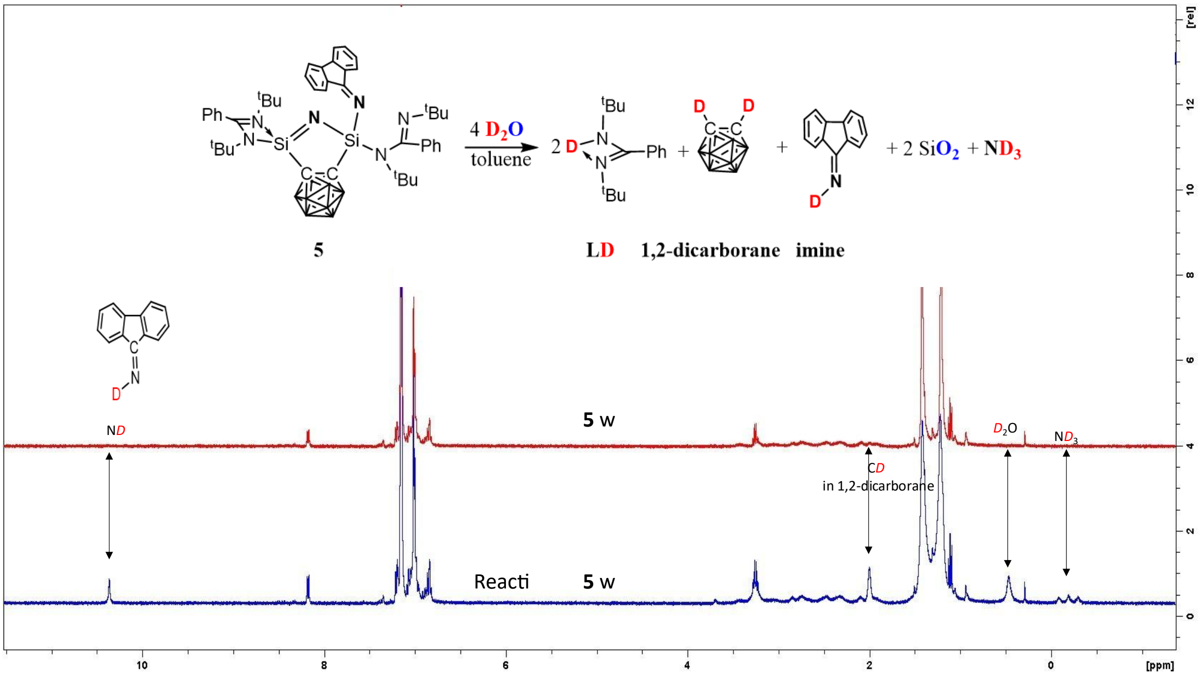

Figure S23. ^1H NMR spectra of the reaction solutions of **5** with H_2O (bottom) and D_2O (top) in sealed NMR tubes(400 MHz, C_6D_6, 298 K)

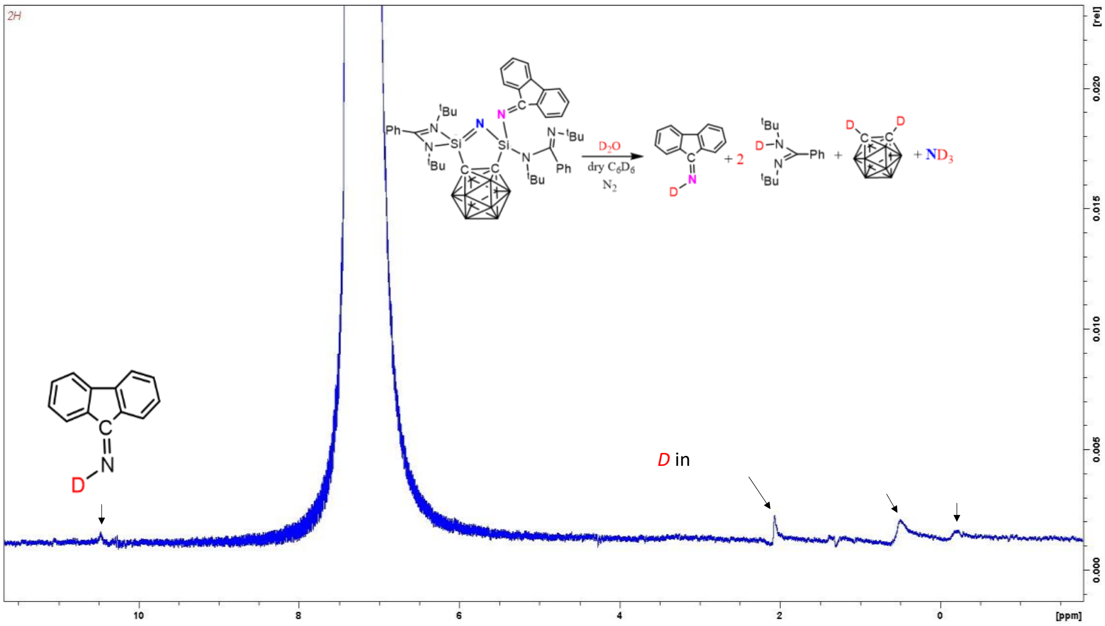

Figure S24. ^2H NMR spectrum of the reaction solution of **5** and D_2O in a sealed NMR tube (400 MHz, C_6D_6, 298 K)

Reaction of compound 4 with H_2O in a sealed NMR tube: To an NMR tube containing **4** (0.010 g) and C_6D_6 (0.5 mL), 5 µL of water was added at room temperature. Within 10 minutes the reaction was complete. The proton NMR spectrum indicated the formation of the corresponding benzophenone imine, **L**H, 1,2-dicarborane, and ammonia (Figure S25). SiO_2 remained as colorless insoluble solid in the NMR tube (Scheme S7).

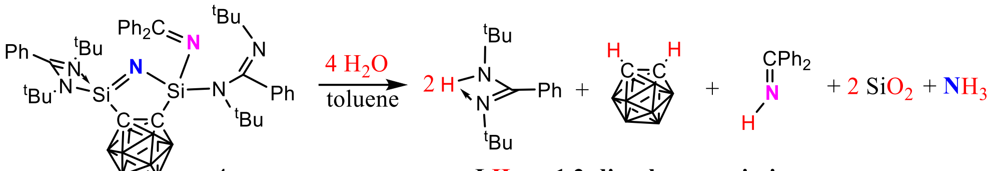

Scheme S7. Reaction of **4** with H_2O

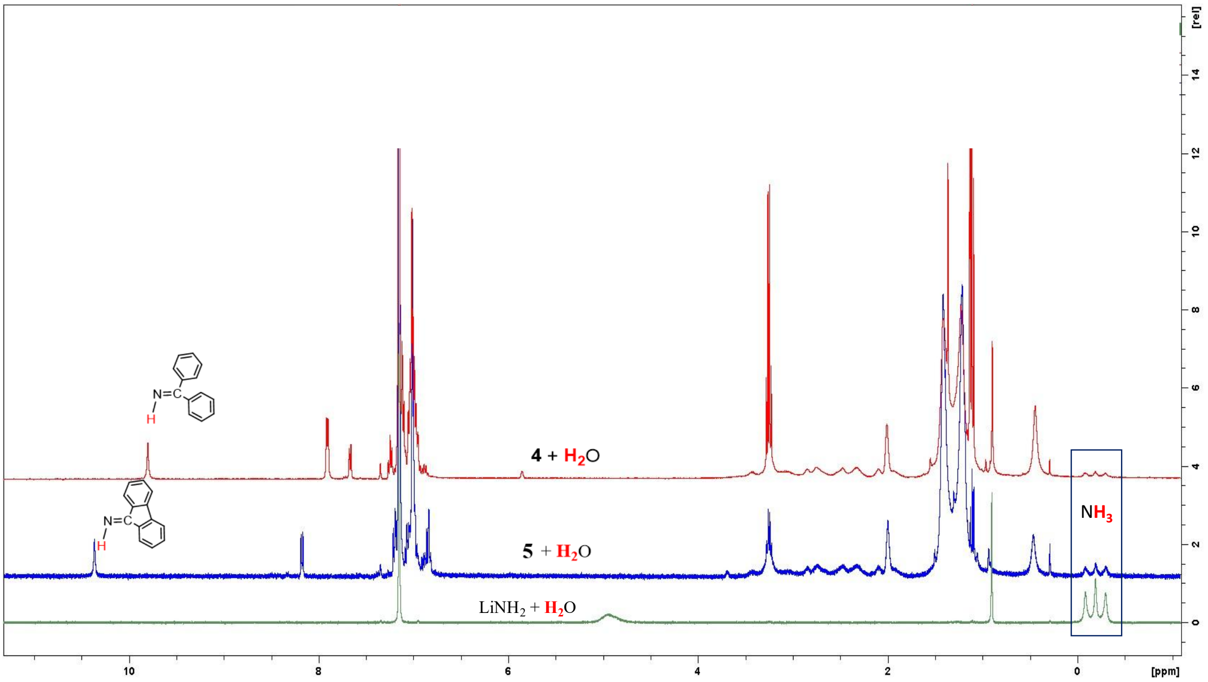

Figure S25. Comparison of the ^{1}H NMR spectra of the reaction solutions of **4** with H_2O (top), **5** with H_2O (middle), and **LiNH₂** with H_2O (bottom) in sealed NMR tubes (400 MHz, C_6D_6, 298 K)

A4. Crystallographic data

Table S1. Crystal data and structure refinement for **1**.

Empirical formula	C71 H78 N8 O Si2	
Formula weight	1115.59	
Temperature	150.15 K	
Wavelength	1.54184 Å	
Crystal system	Monoclinic	
Space group	$P\,2_1/c$	
Unit cell dimensions	a = 24.5276(2) Å	a= 90°.
	b = 24.4294(2) Å	b= 91.6320(10)°.
	c = 23.3938(3) Å	g = 90°.
Volume	14011.8(2) $Å^3$	
Z	8	
Density (calculated)	1.058 Mg/m^3	
Absorption coefficient	0.803 mm^{-1}	
F(000)	4768	
Crystal size	0.42 x 0.07 x 0.06 mm^3	
Theta range for data collection	2.553 to 73.076°.	
Index ranges	-23<=h<=30, -30<=k<=30, -28<=l<=26	
Reflections collected	103385	
Independent reflections	27534 [R(int) = 0.0949]	
Completeness to theta = 67.684°	100.0 %	
Absorption correction	Semi-empirical from equivalents	
Max. and min. transmission	1.00000 and 0.04218	
Refinement method	Full-matrix least-squares on F^2	
Data / restraints / parameters	27534 / 72 / 1536	
Goodness-of-fit on F^2	1.018	
Final R indices [I>2sigma(I)]	R_1 = 0.0630, wR_2 = 0.1618	
R indices (all data)	R_1 = 0.0938, wR_2 = 0.1875	
Extinction coefficient	n/a	
Largest diff. peak and hole	0.545 and -0.320 e.$Å^{-3}$	

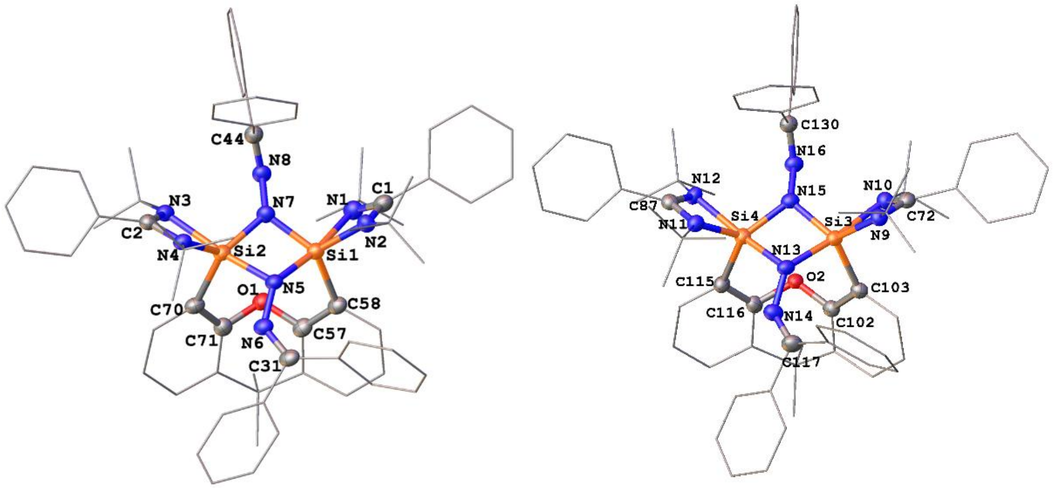

Figure S26. Molecular Structure of **1**. Two independent molecules are found in the asymmetric unit. Thermal ellipsoids are drawn at the 50% probability level. H atoms are omitted for clarity.

***Table S2*. Bond lengths [Å] and angles [°] for 1.**

**Molecule 1**		**Molecule 2**	
Si1-N1	2.053(2)	Si3-N10	2.100(2)
Si1-N2	1.830(2)	Si3-N9	1.829(2)
Si1-N5	1.839(2)	Si3-N13	1.829(2)
Si1-N7	1.776(2)	Si3-N15	1.768(2)
Si1-C58	1.921(3)	Si3-C103	1.909(3)
Si2-N3	2.054(2)	Si4-N12	2.053(2)
Si2-N4	1.822(2)	Si4-N11	1.830(2)
Si2-N5	1.823(2)	Si4-N13	1.834(2)
Si2-N7	1.758(2)	Si4-N15	1.758(2)
Si2-C70	1.941(3)	Si4-C115	1.925(3)
N1-C1	1.295(4)	N9-C72	1.365(3)
N2-C1	1.367(3)	N10-C72	1.307(3)
C2-N3	1.316(3)	N11-C87	1.354(3)
C2-N4	1.357(3)	N12-C87	1.307(3)
N5-N6	1.371(3)	N13-N14	1.376(3)
N6-C31	1.295(3)	N14-C117	1.290(4)
N7-N8	1.414(3)	N15-N16	1.415(3)
N8-C44	1.282(4)	N16-C130	1.275(3)
C57-C58	1.380(4)	C102-C103	1.394(3)
C70-C71	1.377(4)	C115-C116	1.377(4)
O1-C57	1.387(3)	O2-C102	1.379(3)
O1-C71	1.381(3)	O2-C116	1.384(3)

C71-O1-C57	112.1(2)	C102-O2-C116	111.7(2)
N2-Si1-N1	67.4(1)	N9-Si3-N10	66.66(9)
N2-Si1-N5	117.5(1)	N13-Si3-N9	117.9(1)
N2-Si1-C58	107.8(1)	N9-Si3-C103	106.2(1)
N5-Si1-N1	171.0(1)	N13-Si3-N10	173.50(9)
N5-Si1-C58	97.0(1)	N13-Si3-C103	95.9(1)
N7-Si1-N1	92.77(9)	N15-Si3-N10	94.12(9)
N7-Si1-N2	114.1(1)	N15-Si3-N9	118.5(1)
N7-Si1-N5	78.32(9)	N15-Si3-N13	79.66(9)
N7-Si1-C58	134.9(1)	N15-Si3-C103	131.6(1)
C58-Si1-N1	88.2(1)	C103-Si3-N10	86.8(1)
N1-C1-N2	108.6(2)	N10-C72-N9	108.6(2)
C1-N1-Si1	88.0(2)	C72-N10-Si3	86.7(1)
N4-Si2-N3	67.44(9)	N11-Si4-N12	67.27(9)
N4-Si2-N5	108.6(1)	N11-Si4-N13	107.9(1)
N4-Si2-C70	104.8(1)	N11-Si4-C115	106.1(1)
N5-Si2-N3	172.8(1)	N13-Si4-N12	172.8(1)
N5-Si2-C70	98.3(1)	N13-Si4-C115	99.3(1)
N7-Si2-N3	98.06(9)	N15-Si4-N12	98.45(9)
N7-Si2-N4	125.6(1)	N15-Si4-N11	124.6(1)
N7-Si2-N5	79.21(9)	N15-Si4-N13	79.76(9)
N7-Si2-C70	127.8(1)	N15-Si4-C115	127.3(1)
C70-Si2-N3	88.7(1)	C115-Si4-N12	87.4(1)
C1-N2-Si1	95.6(2)	C72-N9-Si3	96.9(1)
N3-C2-N4	107.9(2)	N12-C87-N11	108.4(2)
C2-N3-Si2	87.0(1)	C87-N12-Si4	87.4(1)
C2-N4-Si2	95.9(1)	C87-N11-Si4	95.8(2)
Si2-N5-Si1	96.0(1)	Si3-N13-Si4	95.41(9)
N6-N5-Si1	140.0(2)	N14-N13-Si3	133.8(2)
N6-N5-Si2	110.5(2)	N14-N13-Si4	109.6(2)
Si2-N7-Si1	100.7(1)	Si4-N15-Si3	100.4(1)
N8-N7-Si1	125.5(1)	N16-N15-Si3	125.5(2)
N8-N7-Si2	129.3(2)	N16-N15-Si4	127.4(2)
C57-C58-Si1	113.1(2)	C102-C103-Si3	114.2(2)
C71-C70-Si2	115.9(2)	C116-C115-Si4	115.6(2)
C70-C71-O1	115.5(2)	C115-C116-O2	115.1(2)
C58-C57-O1	114.3(2)	O2-C102-C103	114.1(2)

Table S3. Crystal data and structure refinement for **2**.

Empirical formula	C73.50 H80 N8 O Si2	
Formula weight	1147.63	
Temperature	150.15 K	
Wavelength	1.54184 Å	
Crystal system	Triclinic	
Space group	*P*-1	
Unit cell dimensions	a = 12.7790(3) Å	a= 73.700(2)°.
	b = 12.8987(3) Å	b= 73.640(2)°.
	c = 21.7244(5) Å	g = 69.820(2)°.
Volume	3157.33(14) $Å^3$	
Z	2	
Density (calculated)	1.207 Mg/m^3	
Absorption coefficient	0.905 mm^{-1}	
F(000)	1226	
Crystal size	0.25 x 0.14 x 0.12 mm^3	
Theta range for data collection	3.729 to 72.580°.	
Index ranges	-15<=h<=15, -14<=k<=15, -26<=l<=26	
Reflections collected	23781	
Independent reflections	12171 [R(int) = 0.0191]	
Completeness to theta = 67.684°	99.7 %	
Absorption correction	Semi-empirical from equivalents	
Max. and min. transmission	1.00000 and 0.79401	
Refinement method	Full-matrix least-squares on F^2	
Data / restraints / parameters	12171 / 0 / 753	
Goodness-of-fit on F^2	1.031	
Final R indices [I>2sigma(I)]	R_1 = 0.0345, wR_2 = 0.0905	
R indices (all data)	R_1 = 0.0396, wR_2 = 0.0949	
Extinction coefficient	n/a	
Largest diff. peak and hole	0.399 and -0.312 e.$Å^{-3}$	

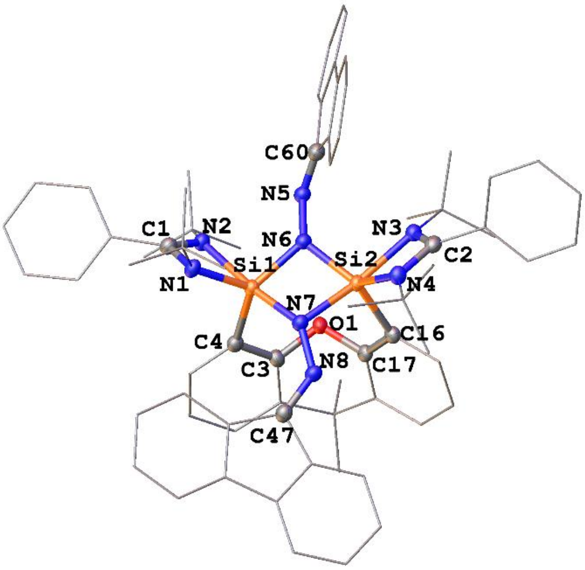

Figure S27. Molecular Structure of **2**. Thermal ellipsoids are drawn at the 50% probability level. H atoms and 0.5 molecule of n-pentane are omitted for clarity.

Table S4. Bond lengths [Å] and angles [°] for **2.**

Si1-N1	1.813(1)	N7-Si1-C4	96.90(5)
Si1-N2	2.100(1)	C4-Si1-N2	85.60(5)
Si1-N6	1.773(1)	N4-Si2-N3	68.95(4)
Si1-N7	1.822(1)	N4-Si2-C16	105.05(5)
Si1-C4	1.919(1)	N6-Si2-N3	97.60(4)
Si2-N3	1.967(1)	N6-Si2-N4	125.09(5)
Si2-N4	1.846(1)	N6-Si2-N7	77.99(4)
Si2-N6	1.790(1)	N6-Si2-C16	128.82(5)
Si2-N7	1.830(1)	N7-Si2-N3	171.60(4)
Si2-C16	1.930(1)	N7-Si2-N4	107.58(5)
O1-C3	1.386(1)	N7-Si2-C16	97.73(5)
O1-C17	1.388(1)	C16-Si2-N3	90.61(5)
N1-C1	1.385(2)	C3-O1-C17	110.82(9)
N2-C1	1.296(2)	C1-N1-Si1	95.92(7)
N3-C2	1.315(2)	C1-N2-Si1	86.14(7)
N4-C2	1.358(2)	C2-N3-Si2	89.18(7)
N5-N6	1.400(1)	C2-N4-Si2	93.08(7)
N5-C60	1.288(2)	Si1-N6-Si2	100.49(5)
N7-N8	1.357(1)	N5-N6-Si1	122.49(7)
N8-C47	1.300(2)	N5-N6-Si2	130.92(8)
C3-C4	1.381(2)	Si1-N7-Si2	97.18(5)
N1-Si1-N2	67.50(4)	N8-N7-Si1	136.43(8)

N1-Si1-N7	116.20(5)	N8-N7-Si2	111.89(7)
N(1)-Si1-C4	108.79(5)	N2-C1-N1	109.2(1)
N6-Si1-N1	115.07(5)	N3-C2-N4	107.8(1)
N6-Si1-N2	95.93(4)	C4-C3-O1	115.1(1)
N6-Si1-N7	78.60(4)	C3-C4-Si1	113.30(9)
N6-Si1-C4	133.03(5)	C17-C16-Si2	115.82(9)
N7-Si1-N2	174.29(4)		

Table S5. Crystal data and structure refinement for **3**.

Empirical formula	C83 H95 N9 Si2	
Formula weight	1274.85	
Temperature	150.15 K	
Wavelength	1.54184 Å	
Crystal system	Monoclinic	
Space group	$P\,2_1/c$	
Unit cell dimensions	a = 21.9499(6) Å	a= 90°.
	b = 13.2486(4) Å	b= 105.151(3)°.
	c = 26.0710(8) Å	g = 90°.
Volume	7318.1(4) $Å^3$	
Z	4	
Density (calculated)	1.157 Mg/m^3	
Absorption coefficient	0.821 mm^{-1}	
F(000)	2736	
Crystal size	0.15 x 0.1 x 0.03 mm^3	
Theta range for data collection	3.513 to 72.994°.	
Index ranges	-24<=h<=27, -16<=k<=16, -31<=l<=31	
Reflections collected	51895	
Independent reflections	14330 [R(int) = 0.1170]	
Completeness to theta = 67.684°	100.0 %	
Absorption correction	Semi-empirical from equivalents	
Max. and min. transmission	1.00000 and 0.16035	
Refinement method	Full-matrix least-squares on F^2	
Data / restraints / parameters	14330 / 0 / 734	
Goodness-of-fit on F^2	0.919	
Final R indices [I>2sigma(I)]	R_1 = 0.0617, wR_2 = 0.1307	
R indices (all data)	R_1 = 0.1134, wR_2 = 0.1553	
Extinction coefficient	n/a	
Largest diff. peak and hole	0.383 and -0.297 $e.Å^{-3}$	

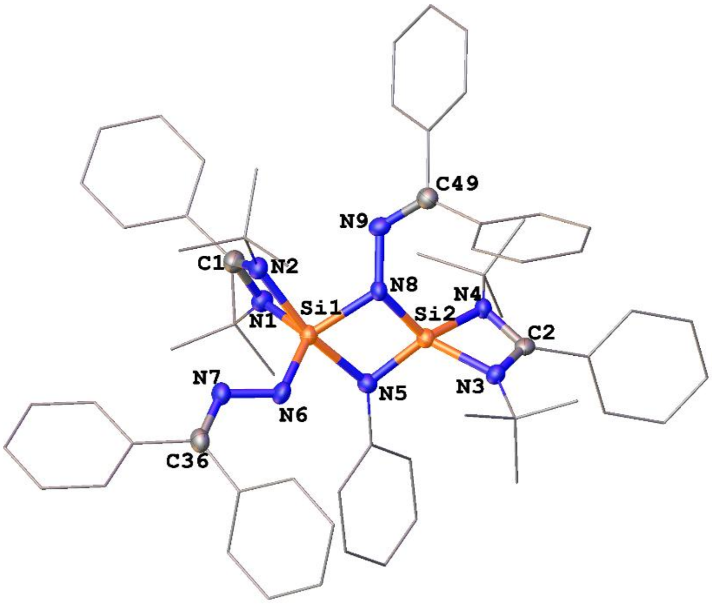

Figure S28. Molecular Structure of **3**. Thermal ellipsoids are drawn at the 50% probability level. H atoms are omitted for clarity.

Table S6. Crystal data and structure refinement for **3**.

Si1-N1	1.965(2)	N6-Si1-N8	125.2(1)
Si1-N2	1.851(2)	N8-Si1-N1	90.7(1)
Si1-N5	1.887(2)	N8-Si1-N5	79.5(1)
Si1-N6	1.700(3)	N3-Si2-N4	73.7(1)
Si1-N8	1.856(2)	N5-Si2-N3	117.2(1)
Si2-N3	1.795(2)	N5-Si2-N4	127.6(1)
Si2-N4	1.795(2)	N5-Si2-N8	91.1(1)
Si2-N5	1.667(3)	N8-Si2-N3	131.6(1)
Si2-N8	1.685(2)	N8-Si2-N4	120.5(1)
N1-C1	1.288(4)	C1-N1-Si1	90.1(2)
N2-C1	1.376(4)	C1-N2-Si1	92.3(2)
N3-C2	1.335(4)	C2-N3-Si2	90.3(2)
N4-C2	1.359(3)	C2-N4-Si2	89.5(2)
N6-N7	1.353(3)	Si2-N5-Si1	94.5(1)
N7-C36	1.311(4)	C3-N5-Si1	135.9(2)
N8-N9	1.405(3)	C3-N5-Si2	126.2(2)
N9-C49	1.291(4)	N7-N6-Si1	117.9(2)
N2-Si1-N1	68.9(1)	C36-N7-N6	117.7(2)

N2-Si1-N5	104.9(1)	Si2-N8-Si1	95.0(1)
N2-Si1-N8	112.4(1)	N9-N8-Si1	122.2(2)
N5-Si1-N1	165.5(1)	N9-N8-Si2	126.5(2)
N6-Si1-N1	99.4(1)	C49-N9-N8	118.9(2)
N6-Si1-N2	121.5(1)	N1-C1-N2	108.5(2)
N6-Si1-N5	95.0(1)	N3-C2-N4	106.1(2)

Table S7. Crystal data and structure refinement for **4**.

Empirical formula	C52 H74 B10 N6 Si2	
Formula weight	949.46	
Temperature	150.15 K	
Wavelength	1.54184 Å	
Crystal system	Monoclinic	
Space group	$P\ 2_1/c$	
Unit cell dimensions	a = 19.5343(4) Å	a= 90°.
	b = 10.9309(2) Å	b= 102.250(2)°.
	c = 26.4836(5) Å	g = 90°.
Volume	5526.22(19) $Å^3$	
Z	4	
Density (calculated)	1.141 Mg/m^3	
Absorption coefficient	0.877 mm^{-1}	
F(000)	2032	
Crystal size	0.41 x 0.23 x 0.21 mm^3	
Theta range for data collection	3.415 to 72.608°.	
Index ranges	-23<=h<=23, -13<=k<=10, -32<=l<=26	
Reflections collected	22473	
Independent reflections	10675 [R(int) = 0.0472]	
Completeness to theta = 67.684°	99.8 %	
Absorption correction	Semi-empirical from equivalents	
Max. and min. transmission	1.00000 and 0.49004	
Refinement method	Full-matrix least-squares on F^2	
Data / restraints / parameters	10675 / 0 / 644	
Goodness-of-fit on F^2	1.041	
Final R indices [I>2sigma(I)]	R_1 = 0.0572, wR_2 = 0.1553	
R indices (all data)	R_1 = 0.0751, wR_2 = 0.1755	
Extinction coefficient	n/a	
Largest diff. peak and hole	0.453 and -0.457 e.$Å^{-3}$	

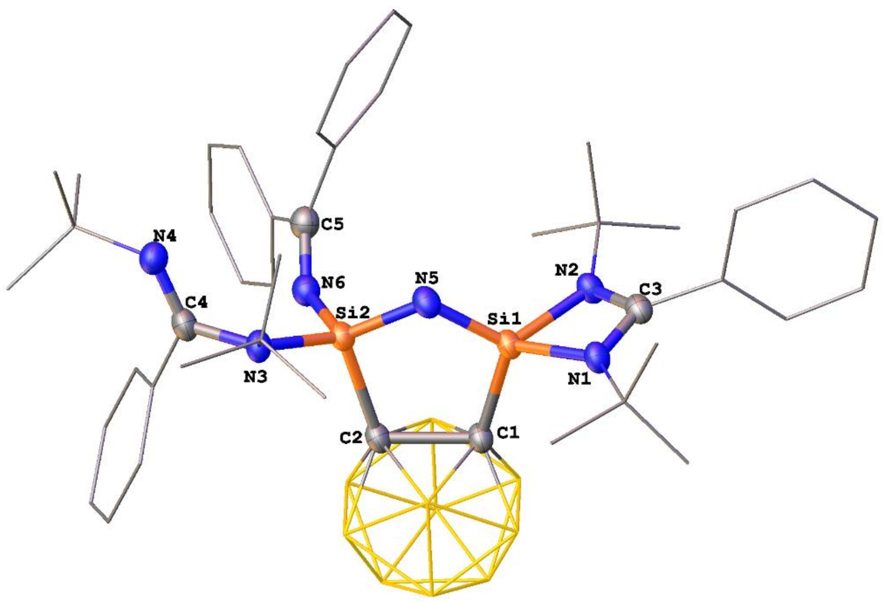

Figure S29. Molecular Structure of **4**. Thermal ellipsoids are drawn at the 50% probability level. H atoms and one co-crystalized toluene molecule are omitted for clarity.

Table S8. Bond lengths [Å] and angles [°] for **4.**

Si1-C1	1.910(2)	N5-Si1-C1	107.00(9)
Si1-N2	1.819(2)	N5-Si1-N2	122.29(9)
Si1-N5	1.622(2)	C3-N1-Si1	90.0(1)
N1-C3	1.337(3)	C2-C1-Si1	105.4(1)
C1-C2	1.717(3)	N3-Si2-C2	113.63(9)
Si2-C2	1.973(2)	N5-Si2-C2	101.94(9)
Si2-N3	1.749(2)	N5-Si2-N3	117.47(9)
Si2-N5	1.681(2)	N5-Si2-N6	119.29(9)
Si2-N6	1.718(2)	N6-Si2-C2	99.31(9)
N2-C3	1.343(3)	N6-Si2-N3	104.02(9)
N3-C4	1.439(3)	C3-N2-Si1	90.4(1)
N4-C4	1.271(3)	C1-C2-Si2	107.1(1)
C5-N6	1.270(3)	C4-N3-Si2	114.3(1)
Si1-N1	1.833(2)	N1-C3-N2	107.1(2)
N1-Si1-C1	113.61(9)	N4-C4-N3	117.3(2)
N2-Si1-N1	72.35(8)	Si1-N5-Si2	116.5(1)
N2-Si1-C1	113.74(8)	C5-N6-Si2	141.1(2)
N5-Si1-N1	124.74(9)		

Table S9 Crystal data and structure refinement for **5**.

Empirical formula	C45 H64 B10 N6 Si2	
Formula weight	853.30	
Temperature	150.15 K	
Wavelength	1.54184 Å	
Crystal system	Monoclinic	
Space group	$P\ 2_1/n$	
Unit cell dimensions	a = 15.58750(10) Å	a= 90°.
	b = 19.8339(2) Å	b= 104.5860(10)°.
	c = 16.1730(2) Å	g = 90°.
Volume	4838.91(9) $Å^3$	
Z	4	
Density (calculated)	1.171 Mg/m^3	
Absorption coefficient	0.949 mm^{-1}	
F(000)	1816	
Crystal size	0.28 x 0.24 x 0.19 mm^3	
Theta range for data collection	3.520 to 72.575°.	
Index ranges	-19<=h<=17, -23<=k<=24, -18<=l<=19	
Reflections collected	35148	
Independent reflections	9493 [R(int) = 0.0234]	
Completeness to theta = 67.684°	100.0 %	
Absorption correction	Semi-empirical from equivalents	
Max. and min. transmission	1.00000 and 0.74016	
Refinement method	Full-matrix least-squares on F^2	
Data / restraints / parameters	9493 / 15 / 610	
Goodness-of-fit on F^2	1.026	
Final R indices [I>2sigma(I)]	R_1 = 0.0372, wR_2 = 0.0993	
R indices (all data)	R_1 = 0.0417, wR_2 = 0.1042	
Extinction coefficient	n/a	
Largest diff. peak and hole	0.295 and -0.320 e.$Å^{-3}$	

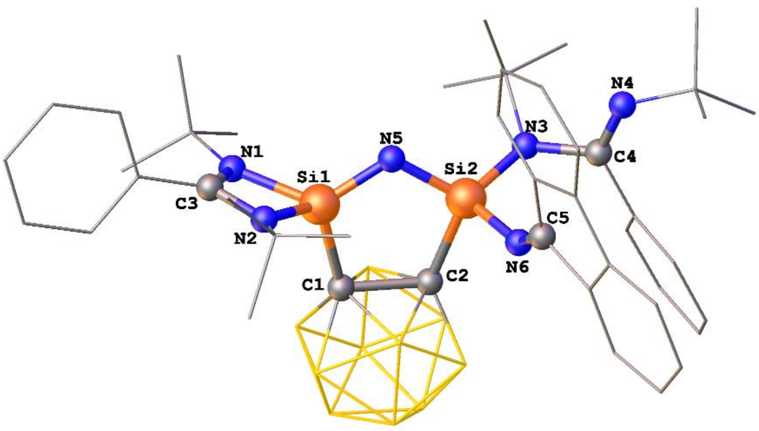

Figure S30. Molecular Structure of **5**. Thermal ellipsoids are drawn at the 50% probability level. H atoms are omitted for clarity.

Table S10. Bond lengths [Å] and angles [°] for **5.**

Si1-N1	1.831(1)	N2-Si1-N1	72.62(5)
Si1-N5	1.617(1)	N2-Si1-C1	114.41(6)
Si1-N2	1.808(1)	N3-Si2-C2	116.78(5)
Si1-C1	1.906(1)	N5-Si2-N3	115.94(5)
Si2-N3	1.737(1)	N5-Si2-N6	120.44(6)
Si2-N5	1.674(1)	N5-Si2-C2	101.83(5)
Si2-N6	1.728(1)	N6-Si2-N3	103.31(5)
Si2-C2	1.968(1)	N6-Si2-C2	97.80(5)
N3-C4	1.447(2)	C4-N3-Si2	115.41(8)
N1-C3	1.338(2)	C3-N1-Si1	89.68(8)
N4-C4	1.264(2)	Si1-N5-Si2	116.84(6)
N6-C5	1.262(2)	C5-N6-Si2	140.5(1)
N2-C3	1.341(2)	C3-N2-Si1	90.55(8)
C2-C1	1.711(2)	N1-C3-N2	107.1(1)
N1-Si1-C1	113.02(5)	N4-C4-N3	116.5(1)
N5-Si1-N1	125.27(5)	C1-C2-Si2	107.40(8)
N5-Si1-N2	121.25(5)	C2-C1-Si1	105.16(8)
N5-Si1-C1	107.17(5)		

B. Computational Details

DFT calculations were carried out with Orca 6.0 quantum chemical package.[9] Geometry optimizations were performed in the gas phase using the r^{2}SCAN-3c functional,[10] which combines the r²SCAN meta-GGA functional with the D4 dispersion correction,[11] a geometrical counterpoise (gCP) correction,[12] and a short-range basis set correction (SRB). Vibrational frequency calculations were carried out at the same level of theory as the geometry optimizations (No imaginary frequency for local minima). The single-point energy calculations for the electronic structures were performed at the PW6B95-D4/def2-TZVP[13] level of theory using the above optimized structures. The solvation effects of the experimentally used solvent (diethyl ether) were taken into consideration by using the SMD[14] solvation model. Transition-state optimizations were located with NEB[15]/CI-NEB[16] method using the r^{2}SCAN-3c functional and the resulting transition-states were characterized by frequency calculations to confirm the presence of a single imaginary frequency corresponding to the desired reaction coordinate. Intrinsic reaction coordinate (IRC)[17] calculations were subsequently performed to ensure that each transition state connects the correct reactant and product minima. Intrinsic bond orbital (IBOs)[18,19] computations were performed with ORCA program at the PW6B95-D4/def2-TZVP level of theory and visualized by IBOview program.[20,21] Natural bond orbital (NBO) calculations were carried out using NBO 7.0 program[22] at the PW6B95-D4/def2-TZVP level. GIAO chemical shifts[23] were calculated at the PBE0-D4/def2-TZVPP CPCM(THF) level of theory.

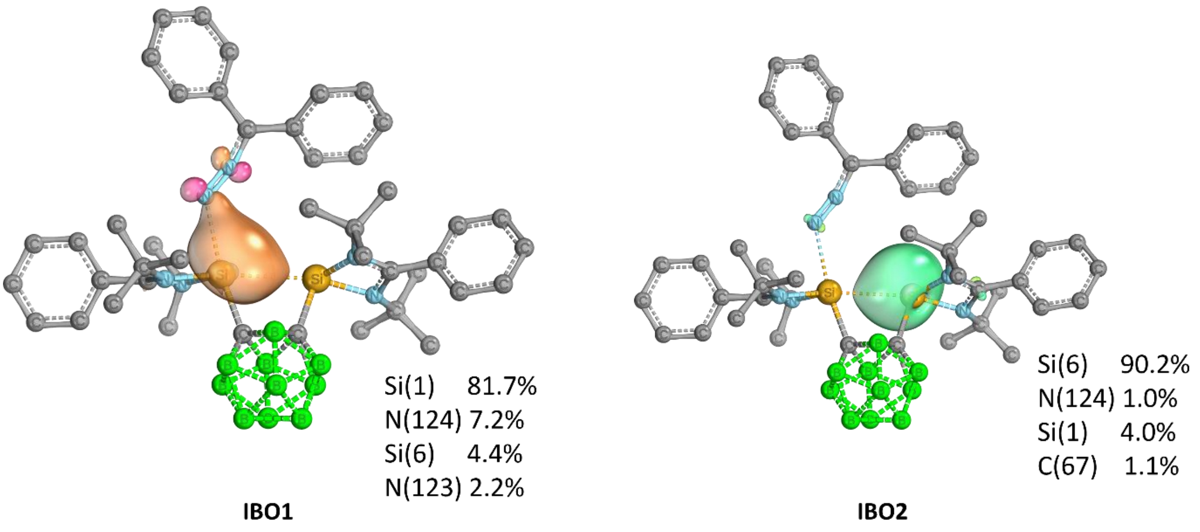

Figure S31. Selected IBOs of **TS1** (hydrogen atoms omitted for clarity). **IBO1** illustrates donation from a silicon lone pair into the π* orbital of the diazomethane N=N bond. **IBO2** highlights the slight participation of the second silicon center.

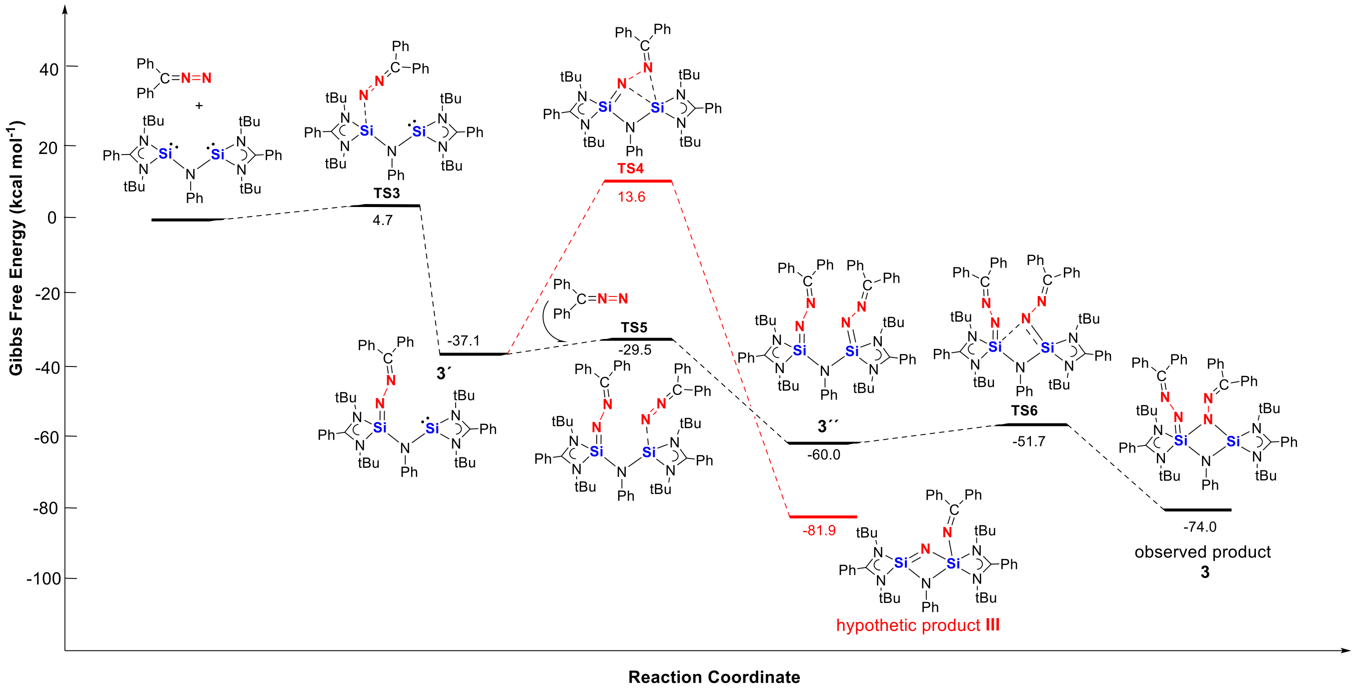

Figure S32. DFT-derived mechanism for the reaction of **PhN(LSi:)₂** with diazodiphenylmethane, leading to the hypothetic N=N cleavage product **III** and the observed product **3** via intermediate **3´** and **3´´**.

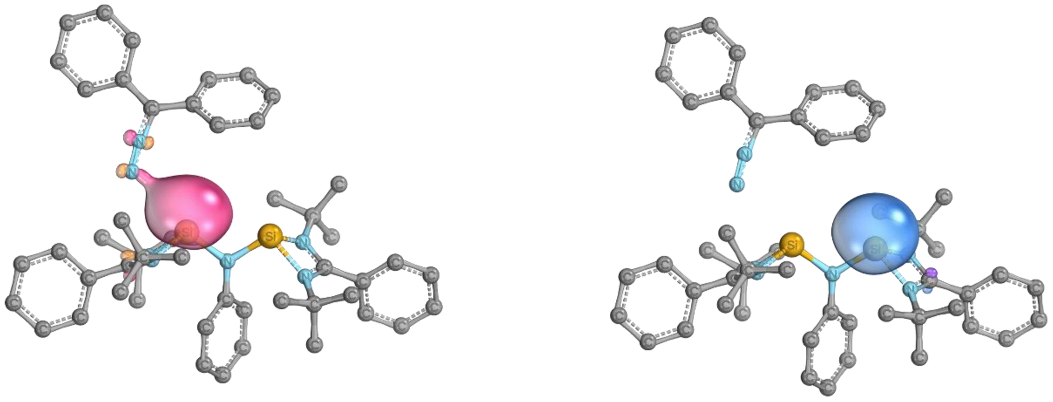

IBO1
Si(1) 90.4%, N(8) 2.6%, Si(2) 1.6%

IBO2
Si(2) 94.7%, Si(1) 1.5%, C(11) 1.3%

Figure S33. Selected IBOs of **TS3** (hydrogen atoms omitted for clarity). **IBO1** illustrates donation from a silicon lone pair into the π* orbital of the diazomethane N=N bond. **IBO2** highlights the lone pair of the second silicon center.

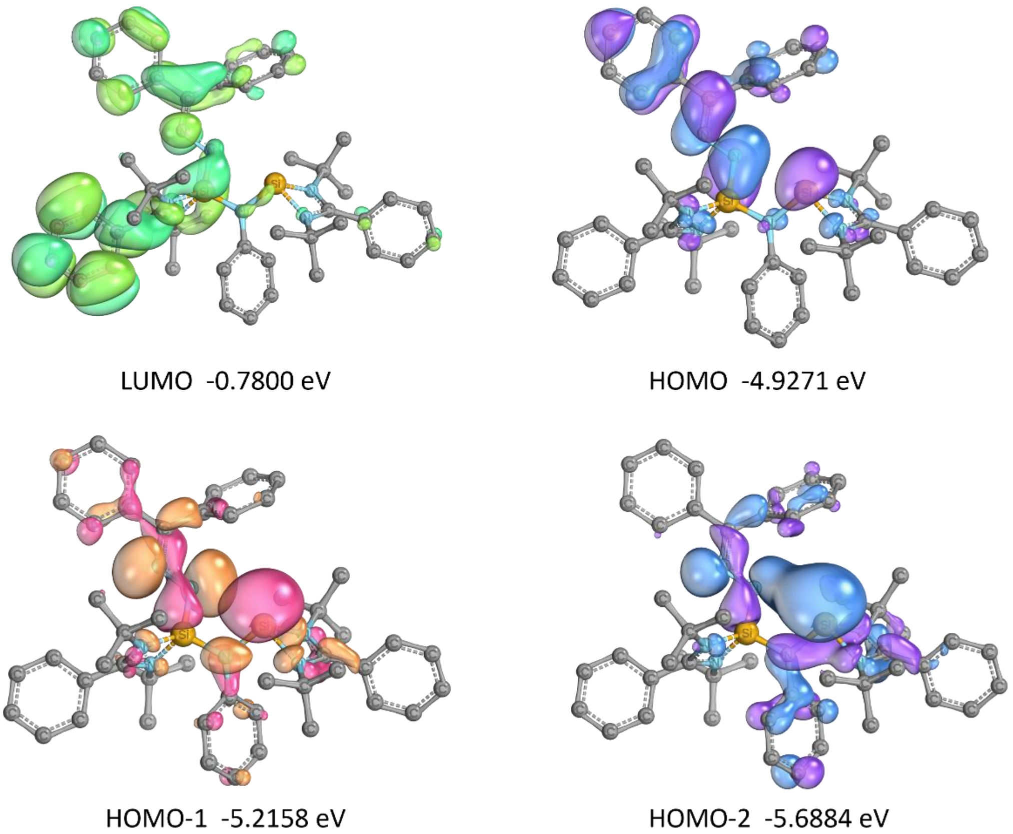

Figure S34. Selected frontier molecular orbitals for **3´** (hydrogen atoms omitted for clarity), illustrating the presence of lone-pair character at the second silicon center.

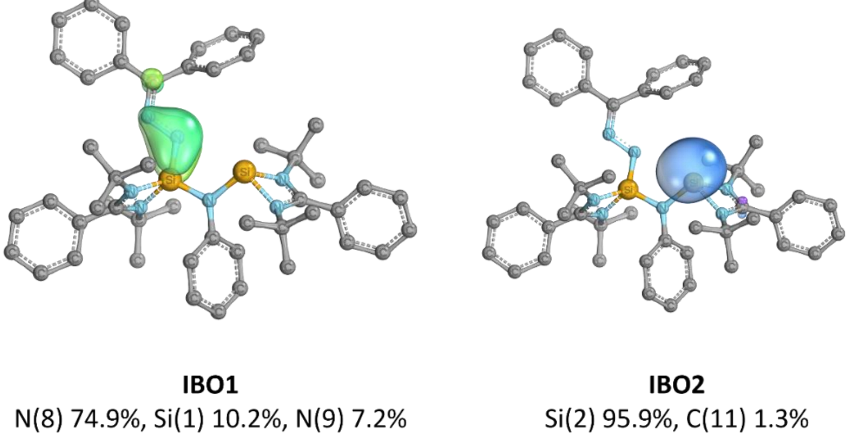

Figure S35. Selected IBOs for **3´** (hydrogen atoms omitted for clarity), illustrating the formation of a Si=N double bond and the presence of lone-pair character at the second silicon center.

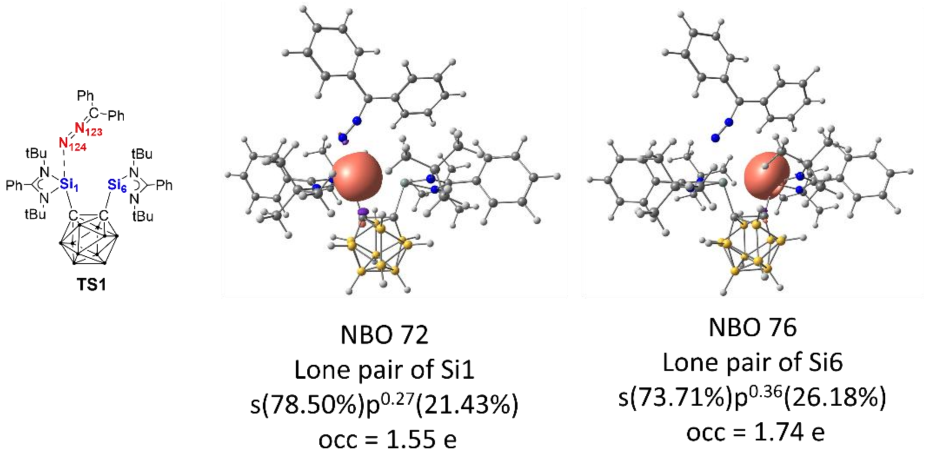

Figure S36**.** Selected NBO orbitals for **TS1**, illustrating the lone pair orbitals on both silicon atoms.

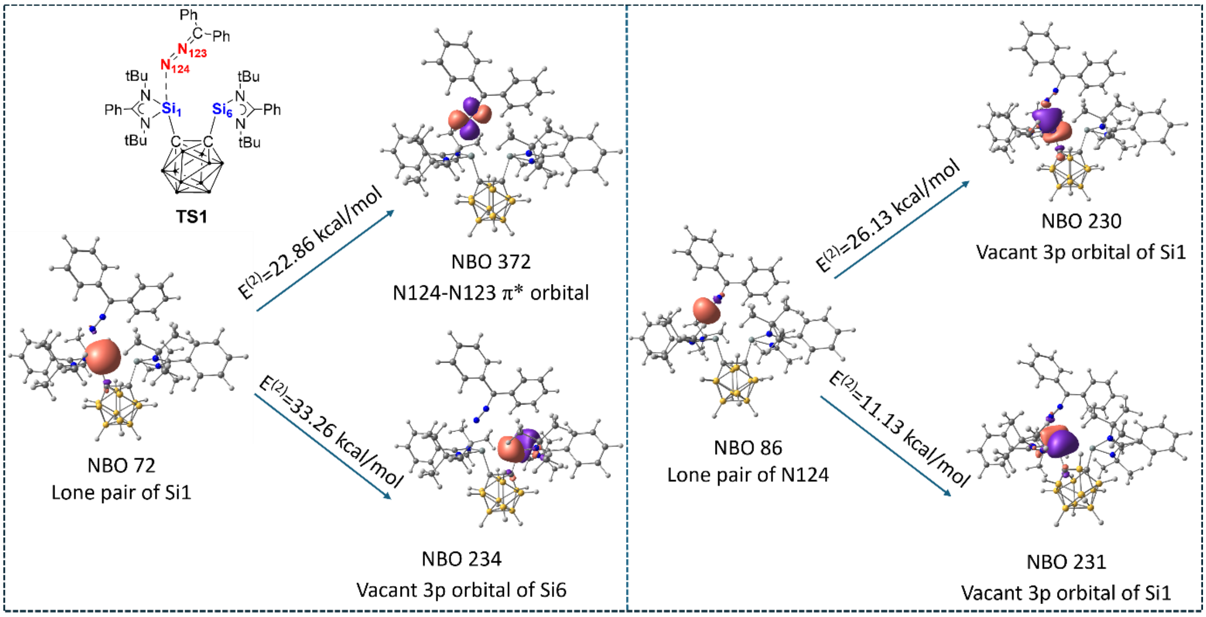

Figure S37**.** Selected NBO orbitals and second-order perturbation analysis results for **TS1**, illustrating the key interactions between **CB(LSi:)$_2$** and diazodiphenylmethane.

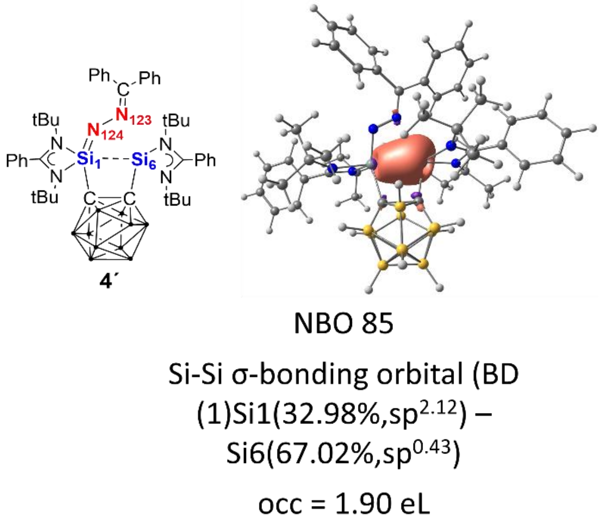

Figure S38. Selected NBO orbital for **4´**, illustrating the Si…Si interaction.

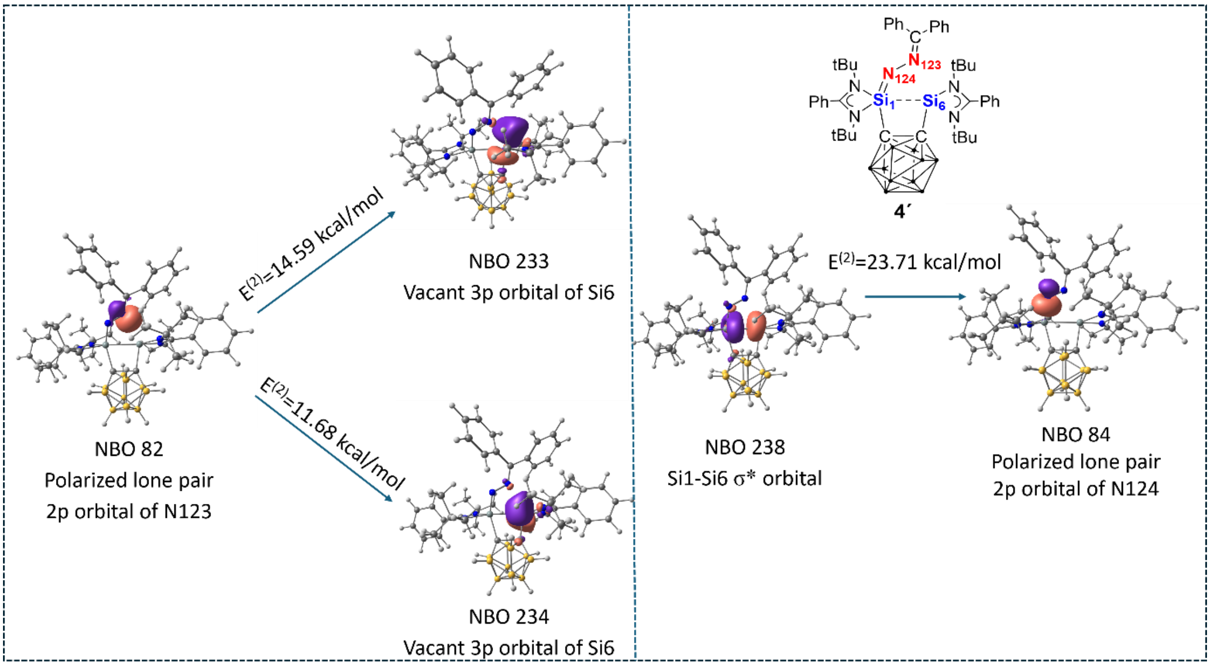

Figure S39. Selected NBO orbitals and second-order perturbation analysis results for **4´**, illustrating the interactions between the N-N fragment of the diazomethane and the two silicon centers.

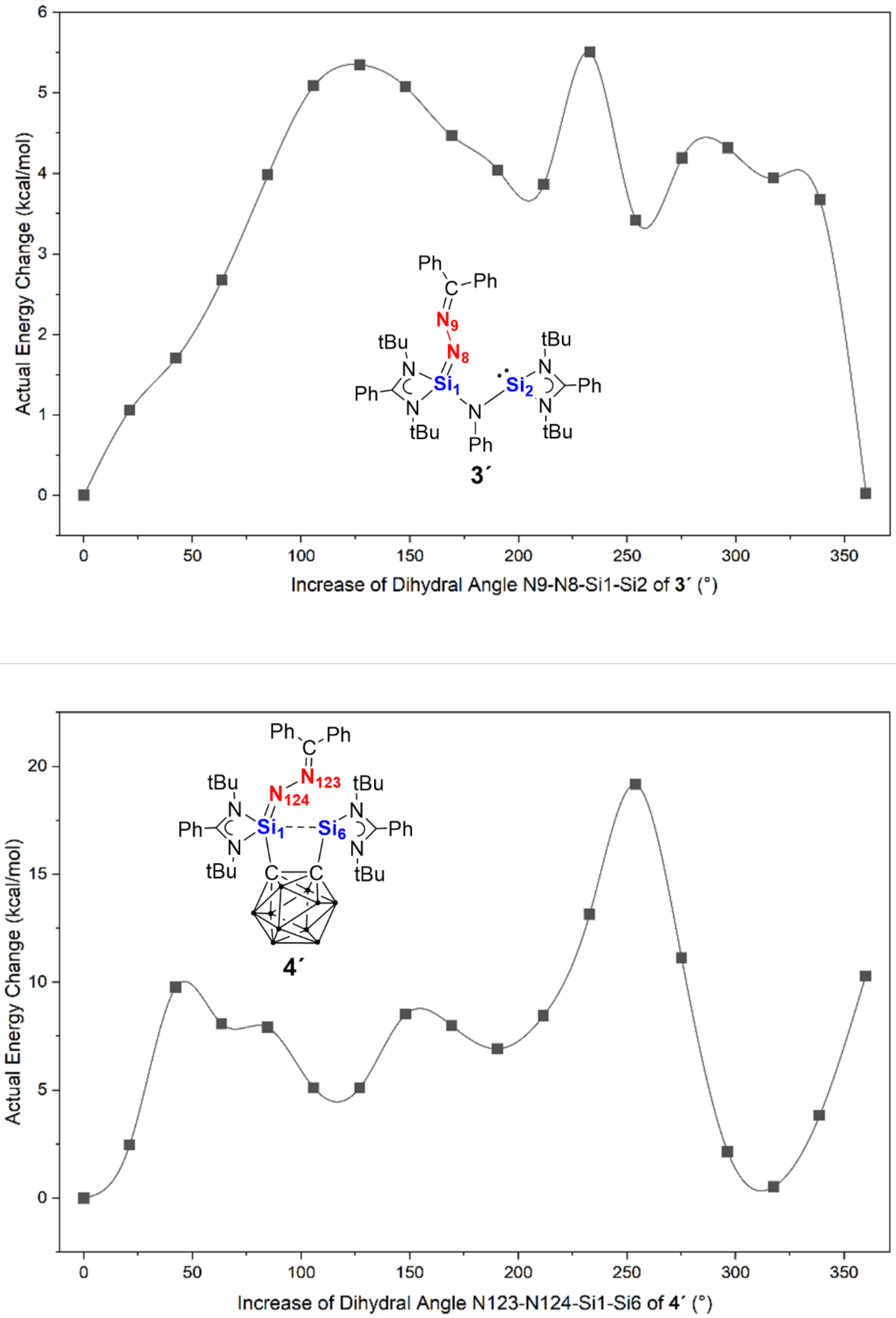

Figure S40. Conformational analysis of intermediates **3′** (top)and **4′** (bottom) obtained from relaxed potential energy surface scans performed by rotating the N–N=Si–Si dihedral angle over 360°. The results show that the originally reported geometries of both **3′** and **4′** correspond to their respective lowest-energy conformers, whereas alternative rotational conformations are energetically less favorable.

Optimized x,y,z-coordinates

TS1

Si	-0.32622831222488	0.47382982338291	-1.45891633047124
N	0.78895515887860	-3.08639035647190	1.64019314520337
C	-0.70215251360119	-2.40970410496299	-0.79837671452052
B	-2.25690256713575	-1.80791217242043	-1.14026160216905
H	-2.74300029120599	-1.01523597145917	-0.41080164171478
Si	0.33832675556124	-1.53778173854200	0.64312676395127
N	-0.80127226315690	-1.72020304640604	2.12989579947662
C	-0.90953779410881	-1.32224029941841	-2.06771058838547
B	0.24191753932647	-2.56529070652578	-2.21524446892030
H	1.39396686609622	-2.30448461984610	-2.16596217743807
N	0.97488515269461	0.95435940776946	-2.70108963851191
C	0.10742642780323	1.75101531458906	-3.34917434955981
B	-0.80284832799172	-2.12628283018716	-3.57273605756303
H	-0.33318272405903	-1.52819283838363	-4.48552848600040
N	-1.09884345183187	1.52706251412724	-2.81126839517405
C	0.40913716567495	2.65448968066652	-4.49052073020711
B	-2.00663518564357	-3.50988403457711	-0.73297413477340
H	-2.34385841669708	-3.90052004744731	0.33695571815692
C	0.76411560913272	3.99274134450386	-4.31034955241755
H	0.84296065768617	4.39601548572872	-3.30501030656341
B	-2.36033547240923	-1.66360074261225	-2.90006143285094
H	-2.97391833105470	-0.76750481201069	-3.36435845367465
C	1.02473849534117	4.79570991081530	-5.41368178383079
H	1.30148944435329	5.83564196898794	-5.26750588606760
B	-0.45644743053889	-3.98250079915916	-1.41171681269186
H	0.25171723853571	-4.72271394277539	-0.82216152749862
C	0.93547270139209	4.26954470033924	-6.70041701705088
H	1.14049920212967	4.90029054120813	-7.56046740735799
B	-0.52445281895897	-3.83622869046806	-3.17861710287823
H	0.13618972514494	-4.53458186373391	-3.87819906580985
C	0.58440353213775	2.93576636854127	-6.88228554662074
H	0.51298320324781	2.52039596197576	-7.88300135594677
B	-2.16298163558406	-3.27498184553997	-3.59914643942685
H	-2.69573016686701	-3.56618550801292	-4.62173935560921
C	0.32010119149397	2.12824570178433	-5.78044222421505
H	0.03732872505136	1.08705077218987	-5.91347301765930
B	-3.07909479485085	-3.06141579052743	-2.07733880159795
H	-4.25874389634961	-3.18787635043501	-1.99888011025139
C	2.44979399745971	0.88850564900751	-2.80626604347485
B	-1.94365252788849	-4.42162749238543	-2.26016347841778
H	-2.30413226710769	-5.55378103466054	-2.31129178340063
C	2.95074313383342	0.17120314904668	-1.54923409216151
H	2.59639965535650	0.67801266946551	-0.64545653679812
H	4.04454742687503	0.15508044414768	-1.54427005571861
H	2.59230962072404	-0.85899946742788	-1.50740983992802

C	3.09550717000471	2.28140388331499	-2.83771730959154
H	2.92654280832434	2.79692950518401	-3.78439758287131
H	4.17722453289705	2.17245046177711	-2.70961284274343
H	2.71328875493040	2.90074589877020	-2.01895909617567
C	2.84615997699145	0.09025446926700	-4.05479830602076
H	2.38662673856183	-0.90267597733871	-4.03920208933657
H	3.93460287269273	-0.02844532595104	-4.09199614842903
H	2.53034812360278	0.60695532425136	-4.96663165409984
C	-2.34830965631811	2.31102971043830	-2.95657826307702
C	-3.28921250269824	1.82994037553558	-1.84600521431017
H	-3.48704505913305	0.75832181235666	-1.92050084825328
H	-4.24484013377870	2.35770256949076	-1.91952358315555
H	-2.85275614702376	2.04267749733097	-0.86269045647157
C	-2.97958424427086	2.06535179047387	-4.33314680006883
H	-2.33338584785744	2.43899177745935	-5.13315705450040
H	-3.93587924109569	2.59550953098015	-4.39681151057203
H	-3.16080764578432	1.00042274242780	-4.49847854346197
C	-2.12028317147321	3.81856374681374	-2.75916624641687
H	-1.53707698872081	4.00185277908801	-1.85175364274766
H	-3.09065679110143	4.31532386559793	-2.65605342630767
H	-1.60775206912016	4.26841203042073	-3.61129141376785
C	-0.10951800605814	-2.77477771543674	2.58797501013331
C	-0.30388051357786	-3.47275944569013	3.88617654906975
C	0.35736104709152	-3.06577394654623	5.04617973112519
H	1.01762351723924	-2.20395789801940	5.01118869151338
C	0.14432412554345	-3.74183176602848	6.24071242509792
H	0.65806976915421	-3.41859126328336	7.14133325885202
C	-0.73149675203561	-4.82412949389239	6.28562649359626
H	-0.89834727191930	-5.34959719397660	7.22131984504482
C	-1.39383780527699	-5.22979281824170	5.13137480471589
H	-2.07746540332683	-6.07286116447707	5.16124041569579
C	-1.18053138012834	-4.55764952391126	3.93202061734893
H	-1.68596713911074	-4.87411093224948	3.02328511936837
C	1.98380961942729	-3.95420211196146	1.73199530079134
C	1.57463927471716	-5.42442255727527	1.89178972198043
H	1.10314241488523	-5.60326070201424	2.86222326222858
H	2.46265835250606	-6.06206882529097	1.82508574518384
H	0.87589469999402	-5.71825834882567	1.10355415495420
C	2.90263647861579	-3.53669744024710	2.88946619595057
H	3.14202740590488	-2.47137303847337	2.81302179704137
H	3.83904198841091	-4.10243005672798	2.83843196054777
H	2.44779271805537	-3.73240087768441	3.86234623272130
C	2.76763599955298	-3.77163466374506	0.42639149175368
H	2.18494822786550	-4.08288625931112	-0.44267124696614
H	3.67898530392802	-4.37632575543648	0.45999447533933
H	3.05844457632096	-2.72224111883582	0.29747046736236
C	-1.90379337372983	-0.95992247508608	2.76400126169030
C	-1.68854816629353	-0.73323678062537	4.26773565589424
H	-1.83980930798499	-1.64226324934900	4.85296267302714
H	-2.41749074220628	0.00818024777148	4.61117446612990

H	-0.68754110452180	-0.33768350697743	4.46746769697528
C	-3.23305953411342	-1.69507300556563	2.54729921777953
H	-3.43872296793993	-1.84174077405720	1.48510027961345
H	-4.05255220272485	-1.11563120458074	2.98619658857141
H	-3.21312731922880	-2.67630369579099	3.03339240817791
C	-1.92431209507467	0.41730610381559	2.09306637260595
H	-0.99583927958223	0.95069734785176	2.31926005342425
H	-2.76656197630006	1.00249038721659	2.47435272353303
H	-2.01997288794662	0.34252166240187	1.00646144352835
C	1.09632088639572	1.92201734221193	5.40918383548960
C	1.84986826951504	0.77235205402734	5.18000087372264
C	2.22797004102635	0.45590273064669	3.87804389630585
H	2.82877966172806	-0.42735498398510	3.68137291834240
C	1.87545021813662	1.28331702164839	2.81947685646756
C	1.12897770015386	2.44890628697417	3.03914462023690
C	0.76255011851121	3.33689582273604	1.92391110891175
C	0.77068637325136	4.80591972778626	1.99971362413340
C	0.03564633510626	5.58166749640773	1.08785098558802
H	-0.56952384808968	5.09210182172440	0.32957953464401
C	0.06019093541698	6.96806980519436	1.15011290258086
H	-0.52381609820157	7.54438758805363	0.43769962138094
C	0.81670395844916	7.62004636647242	2.12075817018514
H	0.83285846254908	8.70458201698972	2.16942530929046
C	1.55583003032585	6.86169393677172	3.02505568318326
H	2.16349713383856	7.35504397904523	3.77872407791524
C	1.54016344179989	5.47439894441870	2.96695138079986
H	2.13992322515282	4.89977071793156	3.66585937170068
N	0.42631453535007	2.80924652974499	0.77655707277259
N	0.07502300773666	2.61723826724117	-0.31580744423345
H	2.19036361862147	1.03217567124230	1.81075552491265
H	2.14504712781505	0.13666675994215	6.01014764197835
H	0.77907130963715	2.17264971741528	6.41765107676204
C	0.73726752963557	2.74962937971350	4.35351637396044
H	0.13900681971261	3.63681009063317	4.54012257248713

4′

Si	-0.06762066636261	0.60290600305970	-1.33114715073480
N	0.90501290901566	-2.39868516380509	1.61855027983763
C	-0.85965056097018	-2.17556259616161	-0.80356146167906
B	-2.36382577247148	-1.49550120152374	-1.21419819462035
H	-2.86059893539782	-0.70970672633880	-0.48398777951267
Si	0.14085904442895	-1.08659347034178	0.52236528781964
N	-0.90588890633786	-1.30593685541657	2.07966515744712
C	-0.95711076017795	-1.05173440071628	-2.05192358598691
B	0.14810953577547	-2.33272581810468	-2.16090406879667
H	1.29888505245146	-2.09164883900791	-2.03447008110677
N	1.22095545837718	1.00076260918072	-2.61811338496457
C	0.35338301899997	1.78070693722690	-3.29419427712957
B	-0.79541450351933	-1.82227303567201	-3.56577329189151
H	-0.24091344777676	-1.23076421258423	-4.43332143577161

N	-0.80094299638945	1.75701708915820	-2.64114411894751
C	0.61033371173892	2.47040978474554	-4.58636258793672
B	-2.20021718907904	-3.22191508854922	-0.83553749950329
H	-2.61661222783922	-3.63283878930874	0.19702502151741
C	1.05775361783001	3.79053693506990	-4.64310894716635
H	1.27678825802332	4.32701855618958	-3.72501613986413
B	-2.36699715662416	-1.30670651173699	-2.97540429017289
H	-2.91097537605353	-0.37223164277115	-3.45645625622630
C	1.24353672525696	4.40677619814787	-5.87521943771249
H	1.59310003269394	5.43413800728260	-5.91408109815564
B	-0.63176740721600	-3.74371720788912	-1.43004617507555
H	0.02697541300752	-4.50993228477387	-0.80924677283380
C	0.98584269055746	3.71061690317283	-7.05308713090732
H	1.13102559963899	4.19568658321593	-8.01386860036871
B	-0.59506031874420	-3.55037905602974	-3.19625209331587
H	0.08084929299107	-4.25222333252091	-3.87790668636768
C	0.54417507242062	2.39213135532322	-6.99675367337065
H	0.34025594937040	1.84423866404337	-7.91178805949971
B	-2.18821064886552	-2.91564853201665	-3.69750948775896
H	-2.67328073161498	-3.17030032040237	-4.75291586124434
C	0.35462037000468	1.77129555376167	-5.76741067597566
H	-0.00718501817394	0.74815802535090	-5.71463122480681
B	-3.18075068464273	-2.69835219017336	-2.22551267609531
H	-4.36611269029568	-2.78918439316845	-2.21661478289096
C	2.66104883533605	0.77287763598815	-2.87325728068749
B	-2.08713778708310	-4.10113362847938	-2.37532462421770
H	-2.49450414004413	-5.21344401143943	-2.47832335473241
C	3.23011250082509	0.15961390819482	-1.59316331438153
H	3.07801593830364	0.82826876175924	-0.73832311625656
H	4.30177724225718	-0.02546219112254	-1.71411680180825
H	2.73779673929258	-0.78874005686541	-1.37803296642913
C	3.40615317697544	2.09096784291275	-3.13563868820838
H	3.18108906584540	2.50357897468059	-4.12022522993138
H	4.48383535199406	1.90228339650041	-3.09334883605209
H	3.15730804192890	2.83353936728966	-2.36989657805668
C	2.86543266893677	-0.18632240609138	-4.05278460290305
H	2.34155817972999	-1.13097149367719	-3.88453184012807
H	3.93297681736804	-0.39601773082384	-4.18171401188286
H	2.49354094718982	0.25585349032106	-4.98204638778098
C	-1.95778771062950	2.67765958292013	-2.74710543399015
C	-3.01380711820539	2.17812158528685	-1.75561299921889
H	-3.38138643399635	1.18588315833204	-2.02626377727401
H	-3.86329666702434	2.86776072367562	-1.75143992147670
H	-2.59148949785715	2.13946600224955	-0.74675047711757
C	-2.55332544041178	2.69687521934250	-4.16040509642827
H	-1.88282057454376	3.16905972840019	-4.88274345212651
H	-3.48641370369855	3.27017240902061	-4.14565212556025
H	-2.77888812917371	1.68224237211278	-4.50282938976410
C	-1.53014898404799	4.09196624561661	-2.32388739773650
H	-1.01246244668173	4.05702572874946	-1.36030371393885

H	-2.41499780459494	4.73085931297449	-2.22724723621108
H	-0.87453337100675	4.54783088333701	-3.07011654210217
C	-0.06851033998811	-2.24303832372867	2.53289766552990
C	-0.20114793133636	-3.01922695226635	3.79304600703358
C	0.45839931090582	-2.61789650570979	4.95471644753971
H	1.05002015373911	-1.70648257570344	4.95164903488647
C	0.31858186866751	-3.36365059455017	6.11922041734331
H	0.82651185673447	-3.04346055806131	7.02419290254090
C	-0.47651535218113	-4.50645092636765	6.12907392819020
H	-0.58467624878803	-5.08615276783969	7.04111373445270
C	-1.13566593414465	-4.90479154580436	4.96937337721962
H	-1.75489319041559	-5.79672187029186	4.97129703877161
C	-0.99987585564426	-4.16368166622017	3.80113534590316
H	-1.49523655789560	-4.47964639433796	2.88693073953211
C	2.08541073881473	-3.29740575596904	1.65049911833172
C	1.68767129842512	-4.75770599853930	1.91099613166707
H	1.37667116648203	-4.92166453673097	2.94455678861682
H	2.55402011516672	-5.39856462772679	1.71660743908291
H	0.87884821041602	-5.06661924559505	1.24244612365016
C	3.09875995884667	-2.83928183625579	2.70662343928368
H	3.44613792430169	-1.82547516226788	2.48899451117504
H	3.96539070063227	-3.50921948876369	2.69299644706007
H	2.67134799152681	-2.86074709484016	3.71129328499017
C	2.75591071081162	-3.20724852412726	0.28021248258866
H	2.10791380325074	-3.59106365927858	-0.51037746182065
H	3.67608616885451	-3.79934761903686	0.28577238267620
H	3.02579699696679	-2.17162720622882	0.05506687160903
C	-2.14262403533538	-0.78816938390141	2.73595083668569
C	-1.96098728992792	-0.59308543913337	4.25014764676240
H	-1.98430669424092	-1.53427377443423	4.80198121951135
H	-2.78984089171063	0.02394833101092	4.61253458461529
H	-1.02699261385008	-0.06672478912843	4.47163531694362
C	-3.31134107266927	-1.74939077717657	2.48139263438483
H	-3.52565446100044	-1.84048020205719	1.41481034788389
H	-4.20666076271311	-1.36811769456457	2.98405926434997
H	-3.09884778934001	-2.74451790105903	2.88189420099154
C	-2.47240778586327	0.58105094921296	2.13695603907341
H	-1.77344309141353	1.33859844311348	2.49665600168967
H	-3.48353978385538	0.86656740471462	2.44247649002871
H	-2.43738772555286	0.57006169489079	1.04619342591183
C	1.34260994312398	1.72349436252437	5.77483737308694
C	2.33026124412925	0.74160923374821	5.74018972240893
C	2.73057736650216	0.22336022187437	4.50967921959791
H	3.51660319694361	-0.52601414944478	4.47326170899937
C	2.15109038627606	0.67308470481644	3.32913844268994
C	1.16817310729101	1.67455384973223	3.34803691510856
C	0.56969532733167	2.19688727220105	2.11073473256351
C	0.21955035487520	3.63566147396476	2.04997630491098
C	-0.99249540080362	4.08673189811739	1.50986150012227
H	-1.69558989122096	3.36660264312552	1.10801381820972

C	-1.29884611128028	5.44205390260943	1.48656704372527
H	-2.24887210651997	5.76862422869630	1.07170420802551
C	-0.39721158885516	6.37903217718828	1.98491557137028
H	-0.63616266221813	7.43840841274777	1.95748672614445
C	0.81249794869043	5.94524351872677	2.52055842029144
H	1.52570712480226	6.66627950389448	2.91062437637411
C	1.11327687840831	4.58905064744154	2.55865828612331
H	2.05627217408968	4.25551626931678	2.98415808693640
N	0.38427033816588	1.35607914299662	1.11990061185502
N	-0.13604328235836	1.74371495905738	-0.02538474770579
H	2.47448455400460	0.28276619342019	2.36839715420360
H	2.79266318283705	0.39311125109038	6.65933074308846
H	1.01459109900745	2.13257155593583	6.72651041609604
C	0.77740191409531	2.19084290791929	4.59596160448595
H	0.01596822302230	2.96510379051811	4.63084730909572

TS2

Si	-0.31167544563529	0.62972005145159	-1.31145650135212
N	0.77797090586326	-2.20754976557795	1.92757384196237
C	-0.73861843438111	-2.09158850363132	-0.62649806313530
B	-2.27924926437849	-1.63608481146007	-1.18984783676240
H	-2.90353056650654	-0.86194307389627	-0.55045316113468
Si	-0.10675737854858	-0.87272541515167	0.87326767420746
N	-1.23658864771060	-1.47537468426927	2.23453172247484
C	-0.85244478842809	-1.10050272520284	-1.95514375260982
B	0.39871273849992	-2.24766223787794	-1.90007334587317
H	1.50112950009216	-1.86398350902506	-1.72994721595045
N	1.01458804871604	1.45418763978886	-2.25446072568747
C	0.10160678919906	2.12771672408344	-2.99155882785711
B	-0.48962552620822	-1.92545090471764	-3.39672539790724
H	0.05269729919298	-1.32031684359762	-4.26387777815793
N	-1.10224076511989	1.81049136014760	-2.51478503528677
C	0.38633644098454	2.99017945537718	-4.16182664433766
B	-1.95598894504080	-3.29755276524246	-0.69119798028890
H	-2.41100768364258	-3.67748854770306	0.33419890313807
C	0.67466534219473	4.34728685589401	-4.01081913720613
H	0.72142128243013	4.77887339100716	-3.01479623731944
B	-2.15671936567685	-1.55951958197043	-2.96110425300307
H	-2.74483286746206	-0.71556771128564	-3.54571336860573
C	0.93158541691504	5.12384668414029	-5.13397180653535
H	1.15689643997714	6.17941173749610	-5.01532429763447
B	-0.29231314920929	-3.66534353731723	-1.11993927153409
H	0.38662030145014	-4.31208421050082	-0.39733061211629
C	0.91081501331276	4.55150965730293	-6.40341421958175
H	1.11515320479765	5.16178464504650	-7.27799427664169
B	-0.14156329038248	-3.59600977221938	-2.89366630630246
H	0.65511827643770	-4.26075126946675	-3.47383263133727
C	0.62946223583966	3.19618328118993	-6.55093469281340
H	0.61114937363765	2.74550993069199	-7.53851704599122
B	-1.74594101464420	-3.17432839240732	-3.55277093393802

H	-2.11027041156030	-3.54413281698577	-4.62218025889594
C	0.36291504629252	2.41374329911995	-5.43362520619694
H	0.12750097804537	1.35824235971584	-5.54205358402478
B	-2.87241281884392	-2.98015111664174	-2.17909950010594
H	-4.03862142227348	-3.19989776339520	-2.25188946131839
C	2.48384288951808	1.32387255990321	-2.46204137595076
B	-1.62194856741998	-4.25504411114867	-2.14560629079599
H	-1.89623829276980	-5.41110931761280	-2.19523896716849
C	3.05055207897736	0.73383416410090	-1.17243097315023
H	2.88153987953762	1.40616695005783	-0.32703084331688
H	4.12581426926804	0.56738020989706	-1.28812223063687
H	2.56871468221298	-0.21919743838052	-0.94547822947757
C	3.13847288200374	2.69014754452039	-2.70327728541066
H	2.93613278734128	3.07634284126677	-3.70393056446171
H	4.22250199958108	2.57200481429757	-2.60521269955324
H	2.80799671449171	3.42031945017204	-1.95832125231507
C	2.77531793265104	0.38914935776891	-3.64196950210595
H	2.33372104079702	-0.59835307086899	-3.48693756898849
H	3.85830579892895	0.26871371194604	-3.75087996832965
H	2.38933528723034	0.80505598208458	-4.57740379419412
C	-2.42301199036580	2.44026653707281	-2.78689917220114
C	-3.44546073566598	1.73827307109276	-1.88914568161273
H	-3.60453880167815	0.70204446917973	-2.19198261576430
H	-4.40337045490963	2.26197208210524	-1.96148190017812
H	-3.11950558301541	1.75691293258041	-0.84287183271112
C	-2.82044646935325	2.28076239404770	-4.25992779848321
H	-2.21083370650846	2.90953254451312	-4.91363079914783
H	-3.86621158262879	2.58097471863241	-4.38217371761703
H	-2.72323947976060	1.23975853754882	-4.58017511285482
C	-2.40268115037045	3.92619290078604	-2.40985364995753
H	-2.21011639392756	4.04561803732863	-1.34134592224791
H	-3.38097577747495	4.36407045575248	-2.63281633865659
H	-1.65010389124882	4.48016749683835	-2.97506865831575
C	-0.33801721156772	-2.38397380061705	2.64097524363879
C	-0.55529786952198	-3.45974998073896	3.64189183311142
C	-0.08252880382730	-3.34514735988272	4.94979191957742
H	0.38859492815575	-2.42196556250028	5.27299728810084
C	-0.25110297111238	-4.39627157422597	5.84246779323985
H	0.11175343330471	-4.30010690833138	6.86143798164889
C	-0.88696031360154	-5.56627211518446	5.43446893220734
H	-1.01347314680184	-6.38808568120674	6.13292802161366
C	-1.36346452034826	-5.67833891452422	4.13162056313507
H	-1.85988953771464	-6.58801033253548	3.80736732465209
C	-1.20100646479526	-4.62856878454965	3.23517628588651
H	-1.54984139065891	-4.72025054507559	2.21044691408722
C	2.08670548809881	-2.90478940329469	2.03951342339602
C	1.96126403834518	-4.42757158381593	2.19520126314815
H	1.60061016299999	-4.71616157053465	3.18430817145083
H	2.95399091558060	-4.86992373354804	2.06022410585260
H	1.29495386916559	-4.85353133853023	1.44076013115157

C	2.88342183031052	-2.33836490591458	3.22551217569952
H	3.05925615571665	-1.26565954167422	3.10903138851622
H	3.85198901291490	-2.84686524205876	3.28308179804977
H	2.36387998759599	-2.50114882650688	4.17339036595606
C	2.86773461238438	-2.60014066474467	0.76099424460892
H	2.40741222913113	-3.07050204370292	-0.11004979928568
H	3.88844183081575	-2.98248551272407	0.85921960104049
H	2.92051324196491	-1.51951300571189	0.60085321810251
C	-2.53246931351046	-1.12202060432523	2.88788320913709
C	-2.39154868473190	-1.08747338625865	4.41811832469782
H	-2.31173536166268	-2.08384785474055	4.85534550994041
H	-3.28486878323691	-0.61437733997008	4.83765546127854
H	-1.52137860191492	-0.49104079287448	4.71447739816783
C	-3.63511274491564	-2.10967080864705	2.48979233983953
H	-3.79708027981801	-2.10166189968404	1.40911569730500
H	-4.57173010777738	-1.82376050757821	2.98081853133368
H	-3.38876313298816	-3.12930708503747	2.79989481265553
C	-2.92491907913582	0.28453376973036	2.43209873797513
H	-2.15676132830377	1.01557032563778	2.70374335592999
H	-3.86371929368541	0.55787108553274	2.92396308942023
H	-3.06434437776593	0.33677242669449	1.35214017601177
C	2.01897745937166	2.90530127558059	5.25932973937754
C	2.00126270856916	1.70072821626698	5.96474523226219
C	1.59573982597217	0.55010310634499	5.29152224535315
H	1.56904815359055	-0.39952398635534	5.82227173476927
C	1.23324893499419	0.59026504599077	3.95478985937469
C	1.27084917017543	1.79288758766240	3.20950170314696
C	0.84425531529277	1.86242998305054	1.83248802537412
C	1.08031136765395	3.10232843116633	1.06070654445469
C	0.06017179961914	3.78279771390968	0.37999700028584
H	-0.94612507959215	3.37986046371130	0.43166530178889
C	0.32031181617468	4.97463075148783	-0.29298114553753
H	-0.49282302214910	5.50146129713625	-0.78547516126172
C	1.59960771740276	5.52328493165264	-0.30073946749162
H	1.79173805737291	6.46572566456317	-0.80669317143182
C	2.62673618294495	4.85978089026056	0.36845591207078
H	3.63324192395047	5.27022080614462	0.36681831552086
C	2.36991164378592	3.66902696954575	1.03280887838960
H	3.17604290256911	3.15905734181831	1.55386683275100
N	0.35896628659695	0.75475061274694	1.20905812114648
N	-0.86118763525588	0.87444816013003	0.29347379927303
H	0.91854862090887	-0.32030980353061	3.46522229724878
H	2.28640330052916	1.66332423097773	7.01126192984332
H	2.30327944401688	3.82487286556711	5.76484009855356
C	1.66631524028260	2.95709962767944	3.92213020516018
H	1.66115123446752	3.91875248175366	3.42079030856352

4

Si	-3.67953468526437	12.34122604077509	16.46614496248095
N	-4.10421071853732	10.21103929542786	20.18938147659652

C	-5.19480382511516	10.05538842082163	17.23474197852091
B	-6.31543674682869	10.89297846968208	16.22761035921403
H	-6.77925500398894	11.90768470318950	16.61046660697374
Si	-4.63902057237740	11.15401539343237	18.79475610156643
N	-4.80542518641475	10.36611258536603	22.40885589803774
C	-4.65766678647944	10.83617791166948	15.82837953452153
B	-3.92010355191019	9.37767604789476	16.31249058158628
H	-2.82648436414468	9.41089978789205	16.74495149135429
N	-2.34900035183072	12.79708852867202	15.29407729544608
C	-3.08029638637249	13.83582808024383	14.84813129930545
B	-4.27855163941529	9.71636543100595	14.60599834354833
H	-3.37500476023567	9.97445322122106	13.87933793523015
N	-4.19490976114469	13.87616898500260	15.58565592396200
C	-2.76398520277840	14.69930934405784	13.68685514608364
B	-6.69759432050581	9.31821158693833	16.92979888411820
H	-7.48254461303719	9.27848950878971	17.81531033675356
C	-1.92136782444899	15.80466890324623	13.79764655668342
H	-1.50062299058714	16.07317520776553	14.76169488443452
B	-5.76510035738504	10.65965323994203	14.55663188530629
H	-5.88109581170480	11.55910581254158	13.78979583181768
C	-1.65507925664925	16.58156572127534	12.67637575598550
H	-1.00493634543482	17.44664045134499	12.76453156380823
B	-5.20677432756767	8.37176288670778	16.97101374119788
H	-4.93657180161631	7.66316175493900	17.87821801996335
C	-2.22354380970404	16.25644799176307	11.44785216614335
H	-2.01130523575188	16.86458372322615	10.57368109288461
B	-4.64492561666424	8.12663227754426	15.30218318716943
H	-4.01327585088886	7.16560001519014	15.00126817191156
C	-3.06703716556670	15.15430573212215	11.34080212797138
H	-3.51205156291477	14.89732409272503	10.38445602374571
B	-5.81304189222948	8.91833308397143	14.21294358548301
H	-6.02911406088081	8.52079003263628	13.11327007213807
C	-3.34239740111653	14.37523189446580	12.45819204441281
H	-3.98914106548481	13.50515285616604	12.38107028336490
B	-7.08240502570002	9.66741262374050	15.22091424108890
H	-8.20502049334738	9.81427822025855	14.85807562016221
C	-0.97480547642414	12.37201154017767	14.92919799921566
B	-6.39053030340684	8.08915140654278	15.67784486459364
H	-7.02482644958193	7.08439538003532	15.63451249082083
C	-0.73592459724237	11.02276039281384	15.60731990737861
H	-0.90466002521723	11.09298909613029	16.68673719631712
H	0.30038681704188	10.71385146041272	15.44332055094526
H	-1.39124690092139	10.25184064335893	15.19516023006438
C	0.03279489608387	13.38963408560138	15.48163435260320
H	-0.05932472057776	14.35586675989868	14.97937259442435
H	1.05172709962956	13.02461902141944	15.31688799172729
H	-0.11525976872212	13.53013731457957	16.55732108016184
C	-0.80735691963668	12.21105845033008	13.41397302002119
H	-1.58216637387137	11.55312185161383	13.00780615394539
H	0.16782781145587	11.75636938508258	13.21235543534996

H	-0.84554110722461	13.16772998293744	12.88816267639832
C	-5.30222978330866	14.86677233910783	15.62793899467143
C	-5.88087705500684	14.79682749939900	17.04351491295848
H	-6.24103830136755	13.79300905151336	17.27716669200935
H	-6.72842235278055	15.48350967523749	17.12803845142260
H	-5.12837197656481	15.06874225802653	17.79147351689847
C	-6.38170851481421	14.50374634255142	14.60283952342791
H	-5.98097857820616	14.53078577472720	13.58446475503050
H	-7.20191996193472	15.22612268386643	14.66728010771887
H	-6.78587515231358	13.50727628168923	14.79469930248848
C	-4.79922306838762	16.29268592076461	15.37352226601248
H	-3.94674915126005	16.53120806945995	16.01852899052991
H	-5.60908067349797	16.98758657809069	15.61546409194431
H	-4.51660859021377	16.46117341142041	14.33239980411584
C	-5.07267238165712	10.00628257146863	21.21501269459311
C	-6.29335612897983	9.25683877170292	20.76660939774185
C	-6.14607443354248	7.94080034187629	20.32205639860782
H	-5.14778933996389	7.52557784858135	20.22586121308790
C	-7.25811970278907	7.16152411468842	20.02995596151533
H	-7.12607677423801	6.13660865409189	19.69526819779334
C	-8.53616089885615	7.69973965600040	20.14745160390434
H	-9.40649730223452	7.09617375687904	19.90643447922786
C	-8.69234689837038	9.02211608606313	20.55437232652901
H	-9.68485272784421	9.45917790363217	20.62395928030073
C	-7.57914519454093	9.79296391287007	20.86674022956821
H	-7.70635412051339	10.82486413392075	21.17390480987074
C	-2.64593796410032	10.03139911101734	20.55626596558822
C	-2.46671572106760	8.86780191608481	21.54383535164786
H	-2.80890381775571	9.11624190479964	22.54655517953643
H	-1.40092499790870	8.62159954797287	21.59418495081784
H	-2.99995752746822	7.97520211447665	21.19709121688881
C	-2.04327529080231	11.31449409683808	21.14207054918728
H	-2.05404817140917	12.11135296201430	20.39142220827076
H	-1.00250315667372	11.13499844962045	21.43910418803139
H	-2.61561363091673	11.63302246812811	22.01615248884593
C	-1.88139400785250	9.64233365968103	19.28753278676422
H	-2.26725160638371	8.70469115310795	18.87355665818793
H	-0.82326128645866	9.50320254397715	19.53363170733662
H	-1.95455138659560	10.42626379191394	18.53226314646269
C	-5.49884141642983	9.97046956040415	23.65053932840531
C	-6.02990111586382	8.52762630147643	23.67700632478984
H	-6.91525302538466	8.38992174198176	23.05369123895978
H	-6.29826509286296	8.26499137441353	24.70653752051706
H	-5.25792292487507	7.82677889856896	23.33925631945177
C	-6.62480001962157	10.95902587871996	23.98039315862909
H	-6.24867902132300	11.98483249767922	23.94135954749960
H	-7.00391358610418	10.76306048306073	24.98998023734352
H	-7.45910584283886	10.86511480002347	23.28136465512393
C	-4.42808422390209	10.10353366728126	24.75166095267553
H	-3.61673017054801	9.38454238704290	24.59688829542054

H	-4.86407897714744	9.91639247360784	25.73939797609128
H	-3.99953171959401	11.11063424647641	24.73695189809732
C	-5.93405847278018	14.61924709983890	23.17301720136992
C	-4.56709525816322	14.86588219367113	23.08168014143265
C	-3.87285805239954	14.48892119623073	21.93531636126966
H	-2.80546699331342	14.67619700803648	21.85853684288066
C	-4.53510058760388	13.86134948106604	20.88853787150381
C	-5.90911469384055	13.61456409240933	20.96919287035773
C	-6.60791011499112	12.90623199416478	19.85799519622496
C	-7.99391330563148	13.35334891072318	19.51120912747200
C	-8.85150003656218	12.45827410173191	18.85861824086967
H	-8.49889498827969	11.44789271641122	18.67339466491682
C	-10.11494180237765	12.86197513977257	18.45089158094889
H	-10.77296130617508	12.15338280893097	17.95568633437688
C	-10.53514669722579	14.17355998527172	18.66690742737805
H	-11.52185459649973	14.49109638994424	18.34145230546840
C	-9.68305423429452	15.07684831282427	19.29536156103577
H	-9.99922834867517	16.10431046739270	19.45268291081895
C	-8.42366345114616	14.66933107857320	19.72254548962514
H	-7.75895558514090	15.38290158024836	20.20042358692240
N	-6.10809098474717	11.95825642655437	19.17352134569238
N	-3.52270883876600	12.21034066550202	18.07957074169238
H	-3.99409758170808	13.56872149740332	19.99210283682920
H	-4.04460526832655	15.34702828031498	23.90373894235834
H	-6.47958233892571	14.89609583125464	24.07091715583813
C	-6.60300193330091	14.00616403067689	22.12089698695506
H	-7.66664365572479	13.80022654474811	22.20463573241839

3

Si	11.07536685383326	4.79191987196018	18.34921791778449
Si	10.65850249120831	4.17727668349231	15.81619925803164
N	11.76214306478857	3.64362719306115	19.83214756466701
N	12.86088085111995	5.16089327718547	18.73037794299232
N	9.36617543074631	4.07263863883482	14.54099173818241
N	11.47141787643865	3.71018209703242	14.26546576491071
N	10.79776070039670	5.61276150801726	16.64533071881050
N	9.74294010710701	5.54271312978574	19.09099148842450
N	9.77431907447129	5.69678598831306	20.42076031646504
N	10.97048916432656	3.28329198732415	17.22697491884919
N	11.74990834800860	2.17298666279879	17.39349791834169
C	12.91531599479351	4.24119486883014	19.74855272496828
C	10.29083796896040	3.78599076680567	13.61517809187254
C	10.35789501714498	6.86276974784468	16.17250984419828
C	10.84988256140313	7.33550364560237	14.94478311434935
H	11.59787881851213	6.74524038911845	14.41665613782269
C	10.40702341671176	8.54142719778350	14.41330201766244
H	10.80345358070170	8.88768037086013	13.46195758197166
C	9.47188144538861	9.30859283192805	15.10326080997165
H	9.12575370582232	10.25255719788431	14.69252630525441
C	8.99403909520191	8.85271949600057	16.33066730033929

H	8.26077170297273	9.43533331839702	16.88277071793863
C	9.42632421113099	7.64547799720609	16.86855624630971
H	9.05134559704736	7.26924475210473	17.81704650890763
C	14.14140316761109	3.89578169860650	20.51800506636409
C	14.34486885829064	4.31063794994529	21.83319377502070
H	13.60877597304892	4.94251544711746	22.32021035716977
C	15.49138776814570	3.91846369150226	22.51784187945343
H	15.64485036875738	4.24925521898973	23.54082016538292
C	16.43354686465171	3.10356549254448	21.89864135669075
H	17.32434530167940	2.79453651937813	22.43771633423999
C	16.22986998541972	2.68390580698258	20.58596059747210
H	16.95912410551704	2.04320776684855	20.09865283032864
C	15.09218890383350	3.08170082302583	19.89727438228493
H	14.91871012756050	2.75484125937497	18.87608835418574
C	11.31362387225843	2.54132612893700	20.70905482491762
C	11.14174923027526	3.05394338646936	22.14676058603681
H	10.50987788129737	3.94619208691348	22.15655812323837
H	12.10770551595225	3.29294746481774	22.59868620389634
H	10.67410948406178	2.27390401579353	22.75740949999177
C	12.26351409655544	1.33532650165364	20.68403567054342
H	11.78315733043713	0.49392492646218	21.19576157753673
H	13.20499616686148	1.54366423894151	21.19877816952006
H	12.48343611434595	1.03675899361700	19.65490727642768
C	13.67380857832406	6.40920888237657	18.71108001458481
C	13.16656582623573	7.30410458105011	17.58191791672969
H	13.19084474256020	6.78991423713291	16.61800979550961
H	13.80329578646122	8.19206149403741	17.51982057674882
H	12.14227219109743	7.63759018582322	17.76638817971426
C	13.50111348587901	7.18099853756538	20.02957548660565
H	12.43586520777075	7.31307893206505	20.25023388903226
H	13.96720460210794	8.16957304305781	19.95195713053563
H	13.97180959503235	6.65882311953619	20.86580246937610
C	15.15646694171153	6.10405352262787	18.45139663927760
H	15.62383435078336	5.57134458119740	19.28171952380018
H	15.70204968419772	7.04274201383929	18.30523843805703
H	15.26349243470133	5.49669841128659	17.54522729260849
C	10.06561574357871	3.59093871778059	12.16741937895512
C	10.01752671123261	4.69136371339920	11.31086077188239
H	10.16278935990280	5.69229332416027	11.70773980839634
C	9.80353401350450	4.49275810052099	9.95219837993425
H	9.76939097221874	5.34730329524815	9.28338627344763
C	9.63509426042707	3.20466533818422	9.45111598044831
H	9.46613157133907	3.05412173192788	8.38908302652106
C	9.68217154588634	2.11039878530454	10.31007734036297
H	9.54557573771192	1.10570886672658	9.92149954987970
C	9.89908673735007	2.29830896839920	11.67028582114477
H	9.91549433351005	1.45435256602154	12.35477290115005
C	7.92475808948346	4.42311907974092	14.44269104707968
C	7.17139790430750	3.39820910272203	13.58992724334871
H	7.44161179630542	3.47034771386722	12.53278381475556

H	6.09729874952028	3.59203142971580	13.67369568632912
H	7.36815464200271	2.37999969200030	13.93671575797624
C	7.74922661267708	5.82941065228719	13.85715863224854
H	8.28806193937787	6.57594966845014	14.44574037919643
H	6.68493423402659	6.08656497346519	13.86715022028174
H	8.09325933680238	5.87396123917805	12.81966501771355
C	7.40036141094332	4.40887197039952	15.88276677961219
H	7.49573874041185	3.41255077819102	16.32448341199897
H	6.34298553958932	4.68767733185967	15.88429380476173
H	7.93546691061418	5.12643176667718	16.51682310575073
C	12.85690150747006	3.47908226690567	13.79968735202750
C	13.75961092949922	3.97616164047781	14.93525645624424
H	13.63855971549772	5.05356872365857	15.08841434826572
H	14.80637944694178	3.77787147591752	14.68644131220847
H	13.52847097273438	3.46993623008773	15.87932861186372
C	13.09282797288138	1.98334099293304	13.56785804826979
H	12.93466134723564	1.42156267623059	14.49286032024604
H	14.12433610656653	1.81816956043683	13.23928226667561
H	12.42225732908981	1.59498848107747	12.79462081954888
C	13.16517711745502	4.26805463794297	12.52251785216123
H	12.62654490703281	3.87669114353274	11.65619878853819
H	14.23697406486997	4.19529903090389	12.31313981887963
H	12.91459767759718	5.32723078058887	12.64485115253490
C	8.79196679437063	6.35325312324109	20.99771692500564
C	7.65286979299322	6.92560529387547	20.23771445176513
C	6.88709784437010	6.13673059363887	19.36894276505738
H	7.10683189047879	5.07687181509743	19.29258550101788
C	5.86516392476628	6.69895014028002	18.61307267994624
H	5.27429323653580	6.06961930016053	17.95207739090781
C	5.59183371572820	8.06289985539712	18.69880183784972
H	4.79550342897644	8.50110494006016	18.10319700226894
C	6.34294339026508	8.85756600946372	19.56069925693845
H	6.13579773890415	9.92173746772122	19.64057407733202
C	7.36102102643823	8.29208131124939	20.32260248331656
H	7.95015172591800	8.91428709140130	20.99206841917546
C	8.89499862437473	6.58315191107009	22.44108300014920
C	7.75694683056066	6.87055912279740	23.21627948709995
H	6.78671223157039	6.92700651571540	22.73047799159886
C	7.85234065340908	7.06595932668157	24.58882886594107
H	6.95342581674168	7.27860456993885	25.16201530761473
C	9.08407262150468	6.98274241210270	25.23265757256615
H	9.15769191553283	7.13789460315068	26.30521445853067
C	10.22406197711881	6.70681974253390	24.47641587041437
H	11.19554079254488	6.65643626383563	24.96274060556958
C	10.13532278759285	6.51753281114518	23.10593765435348
H	11.02410508777973	6.32147479740205	22.51385653778584
C	11.46974223054088	1.05552358816935	16.79765795193199
C	10.24645296641336	0.73028505664761	16.01701259951906
C	8.97345638441761	1.05358884402294	16.50685876769971
H	8.88391997856785	1.64732233207253	17.41050816597589

C	7.83177673071683	0.58057644080507	15.87126417372291
H	6.85411231941906	0.81483310184779	16.28368375125735
C	7.93291054724797	-0.19425827734313	14.71858521129519
H	7.03710087270182	-0.56329685639902	14.22726839213421
C	9.19095046328279	-0.49954143031270	14.20689208663858
H	9.28206420448390	-1.11518675867934	13.31566981364627
C	10.33568854480366	-0.04982943253974	14.85534623506843
H	11.31437607499168	-0.33666129525003	14.48102226209716
C	12.46115678455698	-0.02588438889728	17.01217662348607
C	13.81881229898892	0.28489388811386	17.16979632871555
H	14.12739225350795	1.32161498243492	17.08194383950327
C	14.74925472812500	-0.71038255748248	17.43541845917456
H	15.79779263552791	-0.45060533382301	17.55221515680565
C	14.34407754431878	-2.03850705893449	17.54302631597441
H	15.07171629151493	-2.81792592370264	17.74947394445956
C	12.99928463081066	-2.36049178564506	17.38136925523346
H	12.67241055514982	-3.39228976548658	17.47358959853076
C	12.06600796540565	-1.36640488858918	17.11302199311744
H	11.01692411924012	-1.62656179460168	17.00550633540305
C	9.93551934084550	2.10612514329919	20.19285680607745
H	10.02784089084775	1.60014449215891	19.22841804574958
H	9.27590999737173	2.97359929320490	20.08929816723029
H	9.48671718846752	1.40303434233932	20.90132096439466

3´

Si	-0.07434103653649	0.49227755604944	0.94039211288586
Si	-0.90610828873305	-1.24990870804659	-1.33920729474242
N	0.68916897663785	2.10146871092008	1.38838497533050
N	-1.21636522993295	1.38806263122327	2.08006356558281
N	-1.69572918374648	-0.77474005692972	-3.00572837155901
N	0.32252705772513	-1.45730863513930	-2.78790716551321
N	-0.52509869398366	0.41790362022500	-0.70718061430169
N	0.59342246995623	-0.94650682371142	1.42473180482475
N	0.86352360687994	-1.07358659804145	2.74712300290747
C	-0.30493478691895	2.35671966138291	2.25130884544060
C	-0.64996856792367	-1.25637376801961	-3.68815416500237
C	-0.58539800922237	1.60556960736560	-1.47777223396148
C	0.24682816589185	1.78929068266824	-2.59110189364288
H	0.94586058151700	1.00143478645096	-2.85295264745113
C	0.18113974275227	2.95922974843175	-3.33887697706308
H	0.84005766412876	3.08367605535930	-4.19468761934968
C	-0.70647115114616	3.97594259653744	-2.99322980545911
H	-0.75206461042975	4.88941379405674	-3.57830875608025
C	-1.53294998654710	3.80537545651166	-1.88572248365915
H	-2.23419730011121	4.58709382094937	-1.60491982245473
C	-1.47762636243780	2.63353581053138	-1.13889788198393
H	-2.12978821344545	2.49727534497229	-0.27939287865431
C	-0.40017435135040	3.50338047326283	3.18333202784474
C	0.04474130732690	3.39334070346969	4.50120755292301
H	0.49255987809126	2.46438462735879	4.84211367248628

C	-0.07537236503751	4.47894714148626	5.36144975674224
H	0.27321861552551	4.39274266477165	6.38616289722104
C	-0.63772069822427	5.67041559547277	4.91196954510881
H	-0.73134041083839	6.51531682739121	5.58771883668649
C	-1.07838246045822	5.77937999899805	3.59590628741210
H	-1.51676806069294	6.70758032873810	3.24195115298902
C	-0.96067076813213	4.69858718145068	2.72984065511528
H	-1.30709592847003	4.77116368933701	1.70210342689972
C	1.98066144040602	2.77416775835803	1.15011840932465
C	2.77152076797969	2.93096111703037	2.45484247295541
H	2.85785259309938	1.96522514162639	2.96481440420344
H	2.30137416208426	3.64977840048229	3.13053393556394
H	3.77925835679373	3.29362909175446	2.22733984066097
C	1.75729939332162	4.13310189282566	0.47603548476996
H	2.72349411635126	4.57725156615808	0.21344820290353
H	1.23872053933621	4.82707545358345	1.14464519859671
H	1.16691243723884	4.01414485296427	-0.43870078854517
C	-2.39443387232862	0.99646029955856	2.88076405331827
C	-3.05560386892867	-0.14242702561936	2.09492077201846
H	-3.36787409368535	0.19592325576668	1.10051084310864
H	-3.93886043603337	-0.49869409131529	2.63306159770011
H	-2.36368990561756	-0.98390505025341	1.97160516257407
C	-1.95904013944706	0.48144878729063	4.25890003866558
H	-1.16622986548732	-0.26725867625045	4.15570437342237
H	-2.81447287503531	0.03028794939114	4.77289716328672
H	-1.58878410739791	1.30018624992850	4.88315604031318
C	-3.38051455148124	2.16074482811335	3.02353604006934
H	-2.96836681252066	2.97391384824470	3.62706740012856
H	-4.28932668171397	1.80305347264123	3.51842002911888
H	-3.65959492968626	2.55891506782464	2.04149243394073
C	-0.59843001157197	-1.57596580456828	-5.13926516292801
C	-0.22015842912567	-0.58271912774379	-6.04402316233004
H	0.02041334096901	0.41022882676512	-5.67222075128708
C	-0.14244093071424	-0.86879949059364	-7.40244298277022
H	0.15521392779207	-0.09242460564464	-8.10105577798962
C	-0.44163504717151	-2.14738152328668	-7.86434045439672
H	-0.37868690605727	-2.37115638947647	-8.92523073590947
C	-0.82421052812583	-3.13870073239909	-6.96449918559704
H	-1.06420167564525	-4.13579208925696	-7.32193256948552
C	-0.90354462695656	-2.85545431161540	-5.60564648808996
H	-1.21486162460460	-3.62227580766785	-4.90196435499967
C	-3.05227264340808	-0.42355100665086	-3.45355578413556
C	-3.74463863955567	-1.58954497119658	-4.17395654329808
H	-3.28114411680962	-1.80054130981639	-5.14096902326295
H	-4.79619000818959	-1.33987873847146	-4.35313092493068
H	-3.70453412834816	-2.49498700324172	-3.55867733224452
C	-3.01228140726043	0.81380817575448	-4.36135223578998
H	-2.50387941915499	1.64333356266617	-3.86024963895483
H	-4.03369739258390	1.12540262664211	-4.60667934536887
H	-2.49259703601863	0.59770683214267	-5.29926292144751

C	-3.84239633539604	-0.08854711760225	-2.18218089608797
H	-3.88655152819819	-0.95602878426543	-1.51423479184662
H	-4.86416555835403	0.20290514649703	-2.44372529209491
H	-3.37194812593277	0.74301841556637	-1.64777660307986
C	1.61365662384771	-2.16095897062451	-2.89841231790680
C	2.37468649690656	-1.84337254264172	-1.60514995549279
H	2.58252379419413	-0.76999618137793	-1.53242721411504
H	3.32640297502879	-2.38372226768052	-1.58970478665911
H	1.80279067478964	-2.13573140154544	-0.71652559599720
C	1.39988016942481	-3.67851739331560	-2.99458129487388
H	0.77666115191256	-4.02068890261158	-2.16326220374233
H	2.36284886309615	-4.19758187556101	-2.93482979025511
H	0.92356275972371	-3.95272125262862	-3.94088499701381
C	2.42722348829335	-1.66646548909892	-4.10127462901460
H	1.97666684084010	-1.95595411357261	-5.05420363601094
H	3.43067556699909	-2.10245190416910	-4.05585943337592
H	2.53054463113620	-0.57562474140336	-4.08063977413592
C	1.38640038179324	-2.19729101058803	3.16001731968651
C	1.66652853728120	-3.33965824550859	2.25381323528305
C	2.95131997373300	-3.88807201002242	2.18038152272368
H	3.73783387064073	-3.47889753926334	2.81004057021335
C	3.22904077585791	-4.94141304013659	1.31455487625524
H	4.23449574381827	-5.35142372478145	1.26788549819592
C	2.22010996752769	-5.47237048187555	0.51565239441263
H	2.43220259377371	-6.30022095834470	-0.15576273054146
C	0.93535384036142	-4.93781003337639	0.58716147044930
H	0.13884609504016	-5.34864704132508	-0.02785762696572
C	0.66200334463670	-3.87433756580340	1.43861880965484
H	-0.33400876571645	-3.44594968748792	1.47010069009231
C	1.70928911094106	-2.29752101162129	4.59024053385316
C	1.87227483906113	-3.54680883193425	5.21152959557150
H	1.76802956911118	-4.45138216235099	4.61953403664504
C	2.14817374234092	-3.64066810409474	6.57096208668557
H	2.26263047025252	-4.62081139240301	7.02648952436611
C	2.27085320229045	-2.49342911240601	7.34876456765183
H	2.48956327933286	-2.56804085570640	8.41004010711886
C	2.11569181913693	-1.24526902876179	6.74460946350387
H	2.22265270675912	-0.34042428287531	7.33802002625171
C	1.84445158495579	-1.14658177377119	5.38869776679157
H	1.73611446293453	-0.17671174841972	4.91311848965042
C	2.75428065190023	1.84951288829778	0.20243811643912
H	2.21561898888221	1.73269046044085	-0.74383109363680
H	2.89470300235397	0.85998403502513	0.65164351736057
H	3.73659750857955	2.28005797750193	-0.01388350654609

3′′

Si	0.16206487540868	1.64029432437036	0.62366144942750
Si	0.22474095963324	-0.77270255103114	-1.14826937378448
N	1.65331277351023	2.23384263501319	1.52999126074170

N	0.15603638472785	3.46934224724223	0.59982945920955
N	-1.30784308598113	-1.17445096900847	-2.12293899266203
N	0.72947360697565	-1.26069396549215	-2.82772068988209
N	0.34620424984184	0.96913673096427	-0.95516708322337
N	-1.02725391005384	0.86107765408258	1.49189043615022
N	-1.23538317014613	1.44322924822135	2.70498661906419
N	0.85193302465105	-1.56366927293772	0.11579648763511
N	1.71077474508477	-2.55660879070720	0.33946687262345
C	1.17599060427435	3.48495042088753	1.47858034340670
C	-0.54894590218546	-1.51647810393189	-3.17120955835031
C	0.83941503953603	1.78728752252443	-2.01336410246501
C	1.99834671311841	2.55459302395360	-1.83957487035984
H	2.53696871308017	2.48931907397564	-0.89845772387624
C	2.47008022240323	3.36826916726896	-2.86314838929062
H	3.37327976872114	3.95217321123351	-2.70708198037567
C	1.80778386671284	3.42314604601593	-4.08627290060522
H	2.18082042129483	4.05490303772489	-4.88643529021730
C	0.66191334726018	2.65477721133070	-4.26982664695689
H	0.12912527540578	2.69119715199129	-5.21663039703716
C	0.17918069715761	1.84906991859400	-3.24484939771243
H	-0.72927572976555	1.27428716380915	-3.38261718271281
C	1.65836891794778	4.64797335554607	2.25484178933515
C	1.28995389057864	4.78214028224085	3.59498768935496
H	0.63312476838462	4.04499290014441	4.04834651501899
C	1.76345304772019	5.86045308765445	4.33277726141115
H	1.47279865115899	5.96533760929459	5.37376166867119
C	2.60965080786068	6.79587211357499	3.74282615431595
H	2.98316378326008	7.63353496630605	4.32434508687543
C	2.97882522750335	6.65669989989640	2.40779854524981
H	3.64197860064515	7.38244294157150	1.94667484588318
C	2.50131096236288	5.58644332455703	1.66003005271341
H	2.79084790902339	5.46698856515207	0.61939463816008
C	2.71233107714848	1.60063114856440	2.34905765714454
C	2.09324668680303	1.08623190934681	3.65471411968276
H	1.26298945775649	0.40592043747546	3.44458517839842
H	1.70831832243720	1.91221809312168	4.26063890919030
H	2.85022486669735	0.54950616645139	4.23612824658770
C	3.87248286646032	2.55847786452509	2.63819725363833
H	4.68987938018369	1.98058529516240	3.08021898513090
H	3.60445563620818	3.35189265068707	3.33917642651942
H	4.24293851121709	3.01684913875989	1.71432044350111
C	-0.86031737868005	4.49302359639522	0.27397778284267
C	-1.85582705295819	3.80064786224101	-0.66487600496605
H	-1.35650820314931	3.46967813339696	-1.58205670909992
H	-2.65012392205260	4.50117335947771	-0.93948100894405
H	-2.31373054721530	2.93375567291161	-0.17387902755007
C	-1.59982330303935	4.96566222680631	1.52992115283816
H	-2.03975754521091	4.10947473046522	2.05020760509041
H	-2.40165390742221	5.65520606076633	1.24529140434719
H	-0.93080932563920	5.49297453470598	2.21595374703919

C	-0.21137793606640	5.67216813730018	-0.46054474105813
H	0.44176265115048	6.24520769112056	0.20285026951926
H	-0.99103721504491	6.34680146119897	-0.82937695225164
H	0.37274550884924	5.31272572737821	-1.31503649619654
C	-1.01797258396861	-2.10664051900033	-4.44494091071976
C	-1.26146712196028	-1.29329984059117	-5.55184404886953
H	-1.07229443822549	-0.22493024834333	-5.48959982898067
C	-1.73551642071141	-1.85661185301132	-6.73123520290425
H	-1.92792363274491	-1.22286928352054	-7.59174692089979
C	-1.95975607667988	-3.22846954689589	-6.80877352435277
H	-2.32967066512168	-3.66622104283024	-7.73126439539041
C	-1.70927405379294	-4.03832126671544	-5.70404662477045
H	-1.88405313203188	-5.10869731333378	-5.76058603664625
C	-1.24199330361656	-3.48196985026597	-4.51958948853266
H	-1.06426765478952	-4.10553273179208	-3.64657040308235
C	-2.77422898933205	-0.97145654524021	-2.00747648872217
C	-3.57720954914273	-2.07943600643107	-2.69837149772957
H	-3.51814986074075	-2.02494387468563	-3.78846569760522
H	-4.62835626334857	-1.96836856039318	-2.41250597065178
H	-3.23696574978213	-3.06677117649756	-2.37346025154281
C	-3.14328142619398	0.38888791269674	-2.61690716261990
H	-2.61539174425525	1.19833029405431	-2.10294888017448
H	-4.21940153232475	0.56015507841523	-2.50771955092956
H	-2.90462093282098	0.42074719174017	-3.68673517764826
C	-3.10618859972260	-0.95735226071630	-0.51431287400394
H	-2.94357529375568	-1.93818233246486	-0.06284465431414
H	-4.16072355893734	-0.69633381318167	-0.38015527334715
H	-2.50124875072457	-0.23088171845658	0.04191718912499
C	2.00857260979252	-1.64421488179212	-3.46879546529919
C	3.11109178164754	-0.99510287850402	-2.62289415419042
H	3.02684194115074	0.09671790023352	-2.64441393671967
H	4.08963385463376	-1.27250208354224	-3.02678764664762
H	3.06022818782847	-1.33771689040322	-1.58290600422970
C	2.19187328657115	-3.16535234960241	-3.45585327335926
H	2.18159384653537	-3.54129264917043	-2.42863403146337
H	3.15584625979046	-3.42267660326768	-3.90775957298670
H	1.40447046049031	-3.66698112880618	-4.02594657215909
C	2.08471659922475	-1.10283593289097	-4.90130784839259
H	1.38545376193769	-1.61426886360488	-5.56778152552010
H	3.09593871533419	-1.26184548732622	-5.28969156447169
H	1.87292951953178	-0.02827309750216	-4.91822257709313
C	-2.08256894489055	0.88599139116388	3.52466849719096
C	-2.78525787389831	-0.39350672261805	3.23935711903058
C	-2.06911532848297	-1.58251542581831	3.06960195442710
H	-0.98324810697859	-1.55558571007352	3.09095391622924
C	-2.73970764760099	-2.78837990634975	2.89253056507712
H	-2.17467551967520	-3.71193550578835	2.80049766329748
C	-4.13163689183547	-2.81953242049741	2.83860586990714
H	-4.65143909207843	-3.76408456978222	2.70134417837730
C	-4.85183713978074	-1.63626718304968	2.97662348969091

H	-5.93784314720118	-1.65056518677197	2.93664284229949
C	-4.18182457905352	-0.43529338787951	3.18786190416627
H	-4.74292590791264	0.48595289753285	3.32593683866635
C	-2.32238346118948	1.55753249979659	4.81028427215208
C	-2.87115780564746	0.86088768587970	5.90018744250280
H	-3.13396102133338	-0.18634172182269	5.78340077196248
C	-3.06574582708633	1.48669399016619	7.12642541326279
H	-3.48457646066702	0.92029106155643	7.95402186869739
C	-2.72453475494727	2.82426848462648	7.30178516245340
H	-2.88228271374929	3.31241776334727	8.25910603921225
C	-2.18798718045244	3.53211286819482	6.22563225238597
H	-1.93616015833394	4.58393660903107	6.34048439845460
C	-1.99412750416029	2.91259165608858	5.00070208837399
H	-1.59843847825831	3.46914697726169	4.15866076263733
C	1.47419595862862	-3.81868563434283	0.10402565715789
C	0.21574599062806	-4.39567255535124	-0.42095043521927
C	-1.02830281897190	-3.96865673914882	0.05891110774278
H	-1.06397417630670	-3.15937948696200	0.77935499324586
C	-2.19909107233244	-4.59678100488457	-0.35169047786996
H	-3.15099310454916	-4.27627032969724	0.06511586406057
C	-2.15845217444825	-5.63402597639847	-1.28033025696921
H	-3.07585342893273	-6.12331700483045	-1.59621867462655
C	-0.92937078284618	-6.04505944033722	-1.79257189289538
H	-0.88261088620769	-6.85688000444525	-2.51426180163885
C	0.24409337651558	-5.43518651533559	-1.36392336606770
H	1.20154438159603	-5.78159905517865	-1.74395373409777
C	2.59609876354388	-4.73383058476964	0.41628678388455
C	3.92453666713408	-4.28986257951858	0.32735444330593
H	4.10861601393831	-3.26857424310255	0.00896091282344
C	4.98019009035859	-5.13296438703148	0.64577450640777
H	6.00110839496370	-4.76893789583179	0.56491480073962
C	4.73967916973969	-6.44315304326356	1.05560492501800
H	5.56709918379226	-7.10338165218200	1.29939597380419
C	3.42714260899271	-6.89686439124208	1.14751479418357
H	3.22419517405743	-7.91236589163936	1.47684226925448
C	2.36788849909854	-6.05441978404298	0.82983755887171
H	1.34793485323794	-6.41732914521442	0.91959084188381
C	3.23345644204412	0.41941568428767	1.52320070099410
H	3.70445364367732	0.77136805879285	0.59735427931778
H	2.42847421691150	-0.27380323250857	1.25870370373917
H	3.98125894996250	-0.13328693544743	2.10010668769414

III

Si	0.54805520061870	1.41127215554478	1.00661996044140
Si	0.28087549360673	-0.60774003842305	-0.28189475911999
N	1.17345044009587	3.14037197261874	1.19418849458751
N	-0.23199809031150	2.16571520711203	2.50502294646579
N	-0.87896392906549	-1.27571570510121	-1.93463491797056
N	1.28837942849224	-1.25700924314823	-1.69052345623990

N	-0.43880146572068	1.08206411802010	-0.36802098463252
N	1.34487695845396	-0.02408886888152	0.99948812443847
N	-0.64107651655901	-1.75183700402457	0.66703952621953
C	0.46029423285488	3.29569484273152	2.32175741926599
C	0.24520916588482	-1.66479440859425	-2.47305643246922
C	-1.22602390584170	1.89056359402531	-1.17293039699262
C	-1.16011139303283	1.82960693657248	-2.57624571738307
H	-0.48743842138387	1.11123659773838	-3.03365504500867
C	-1.93542195238279	2.67272079935160	-3.36223161707930
H	-1.86621706970527	2.60341901181924	-4.44520162052874
C	-2.78430776607794	3.61303470255776	-2.78134255775977
H	-3.38582983374494	4.27103213569335	-3.40074317391495
C	-2.84631246965676	3.69503597925267	-1.39184510891886
H	-3.50671225665489	4.41903517778323	-0.92038485917937
C	-2.08432659681595	2.84668060489996	-0.59834022056024
H	-2.15698503249560	2.89259862757967	0.48666991503090
C	0.44079745835695	4.48620456155087	3.20209087884901
C	1.47793338386147	4.69413860802504	4.11198246741036
H	2.30925738622981	3.99548931045576	4.15058982496405
C	1.43410910404445	5.78806340519789	4.96939753950889
H	2.23710281430135	5.94410870117269	5.68326145144411
C	0.36650929795715	6.67909306343037	4.91140291236961
H	0.33628252251538	7.53432795702342	5.57996659518501
C	-0.66284645666249	6.47448369953744	3.99598868499445
H	-1.49545836622833	7.16968462847293	3.94713539166725
C	-0.63227513762007	5.37629555865833	3.14581132900734
H	-1.43590019629249	5.20427072883366	2.43504991252692
C	1.83664451156622	4.14589989482229	0.33473094472196
C	3.07856765347491	4.71299818137026	1.03317495503702
H	3.74186705368994	3.90248970289930	1.35222551736795
H	2.81132980420104	5.31560108302398	1.90560210611653
H	3.62747372096524	5.35513553272868	0.33665751591885
C	0.86893711521484	5.27086836526894	-0.05095038743952
H	1.35239212637763	5.93273403580223	-0.77713630162457
H	0.58671007221253	5.87534030370867	0.81567932906391
H	-0.03533283708452	4.85793609852254	-0.51165690246162
C	-0.78499164603572	1.54047583366975	3.72870329145365
C	-1.75564052409336	0.46077253830135	3.23962375759892
H	-2.58668049802795	0.90909489928092	2.68317994740792
H	-2.16505095543712	-0.08636576445248	4.09461936857343
H	-1.24958415170942	-0.25631428347546	2.58368004549804
C	0.37300125726684	0.88736125807683	4.49684145011221
H	0.90309566862461	0.18363980421175	3.84608698168600
H	-0.01207938033588	0.34701198291243	5.36805119555770
H	1.07978261275737	1.64803115740691	4.84922359473569
C	-1.53093440822024	2.53819252057993	4.61718490730491
H	-0.86345188385755	3.26852047701048	5.08164753585307
H	-2.02902779903166	1.98124395337089	5.41710096126689
H	-2.29826752733666	3.07507765368506	4.04929970301131
C	0.40665278992437	-2.52519299219199	-3.67695480405368

C	0.31390095640414	-2.00657165730361	-4.96799815638470
H	0.09381533730101	-0.95242937129272	-5.11187729174395
C	0.49564234513538	-2.83823758925418	-6.06797681955574
H	0.42896822826364	-2.42715959939522	-7.07104145764836
C	0.75636968834772	-4.19317309338194	-5.88399387355461
H	0.89576979103846	-4.84158326878548	-6.74415758221210
C	0.83648761435295	-4.71466212354579	-4.59509410900505
H	1.03927862781518	-5.77112953678789	-4.44436784630662
C	0.66954032412938	-3.88405567579720	-3.49395431920657
H	0.75847112382803	-4.27888670435580	-2.48491949957302
C	-2.25799645546232	-1.71924124076957	-2.23405027957362
C	-2.45715350455173	-3.20318133999161	-1.88717687952005
H	-1.78394811067068	-3.84159647359477	-2.46680368177135
H	-3.48685564936398	-3.49906373138463	-2.11771321968931
H	-2.28182642257121	-3.37531481094261	-0.82286183103780
C	-2.64287308836077	-1.48160005254185	-3.70324648931626
H	-2.38646911592005	-0.46115407467997	-4.00751084047300
H	-3.72681025381908	-1.60177637605919	-3.80758434549566
H	-2.16012219072881	-2.19070188677833	-4.37909087299172
C	-3.18793154826232	-0.86866410126476	-1.35710437282655
H	-2.85435339171912	-0.89289673279381	-0.31667405226168
H	-4.20938309295758	-1.25930623090661	-1.41561949833585
H	-3.19370628469340	0.17311997405592	-1.69009443786129
C	2.66757931764969	-0.97485759827919	-2.18992473773397
C	3.58111016187071	-0.65151089453993	-1.00422176053414
H	3.17058994723641	0.13845618640918	-0.37408116953322
H	4.56182162663118	-0.35701460956736	-1.39261784829465
H	3.71327922901476	-1.52992166344846	-0.36648659675158
C	3.29882684952415	-2.16655043564688	-2.93089287550795
H	3.19838793975936	-3.08358880642254	-2.34275695337359
H	4.36631924113885	-1.95885966414498	-3.06256757717267
H	2.87141829211346	-2.33711137912920	-3.92036341890790
C	2.61319094954392	0.24219773070609	-3.12614323621264
H	1.98946825138264	0.03744492663972	-4.00278348863132
H	3.61784251073365	0.49925832688040	-3.48115873582163
H	2.19872278003748	1.10793319046056	-2.60160253654866
C	-0.54119072273085	-2.95175986909484	1.06929785835589
C	0.53473889545578	-3.88741704461942	0.61272421192038
C	1.87190940364350	-3.48685984175246	0.66872926421761
H	2.10504483131829	-2.50072047926383	1.06655435071247
C	2.87448021700896	-4.34255838348735	0.22545878651670
H	3.91434424663448	-4.03266024558423	0.28789707253067
C	2.55402244812327	-5.59198832399216	-0.30099410743160
H	3.34014916615977	-6.25349693142985	-0.65438578227328
C	1.22164645716869	-5.99279874763506	-0.36622731621268
H	0.96411995776298	-6.96578490908381	-0.77626242410996
C	0.21811078145681	-5.15068637156473	0.10224579887777
H	-0.82131394732367	-5.46796722015620	0.06533553857049
C	-1.53106974328639	-3.48818733540919	2.05303627242872
C	-1.23164818845005	-4.58703691135367	2.86766370177030

H	-0.26986841566539	-5.08143957424339	2.76922886595851
C	-2.14505461063359	-5.03643596886856	3.81612849670028
H	-1.89153687134135	-5.88249719816762	4.44876114582189
C	-3.37682109572833	-4.40537231502254	3.95705160316165
H	-4.09314746648845	-4.76193317926499	4.69184784137298
C	-3.68524221439380	-3.31154569001238	3.14928425212909
H	-4.64611819969905	-2.81458168011443	3.25130921467997
C	-2.76977461180417	-2.85488551754760	2.21237886725260
H	-2.99273762302906	-1.99448901619680	1.58986640847095
C	2.26055835439563	3.39215079003041	-0.92856116145203
H	1.38161183644345	3.01424393420785	-1.46213943535366
H	2.91098361809426	2.54738128962325	-0.67781904912798
H	2.80612090181170	4.06443991525531	-1.59739161423491

TS3

Si	-0.30839245614407	0.33253394832241	1.04251615312113
Si	-0.87516172963348	-1.26083917017926	-1.35945455942266
N	0.47260940171355	1.99411005826838	1.37698112267161
N	-1.54554729211801	1.47750660573737	1.88491886785271
N	-1.71156994149200	-0.87544300162371	-3.02024703345268
N	0.34479645426063	-1.44634539129141	-2.83379779235993
N	-0.58983324816810	0.37667356798673	-0.70851084324817
N	0.43879877095909	-0.22072116593119	3.12969298706715
N	1.08751418960725	-1.09852940633784	3.57272055808398
C	-0.56974922116340	2.36592457493599	2.12786263071829
C	-0.65143899879839	-1.29662185491065	-3.71787664305937
C	-0.58183553704042	1.56848980337069	-1.47269807450226
C	0.33101365349178	1.74974690226034	-2.52308236845231
H	1.04206395272983	0.95892173941695	-2.73800423590428
C	0.32638422449250	2.91834003300037	-3.27670902730986
H	1.04750492274508	3.03685539272764	-4.08189858962870
C	-0.58025092134961	3.93877874543401	-3.00072407819250
H	-0.57960478301857	4.85128782233266	-3.58910864264744
C	-1.48618259965676	3.77179719376123	-1.95515871625516
H	-2.20413596522114	4.55621032703062	-1.72830538914293
C	-1.49075869848729	2.60385328598804	-1.20253104290637
H	-2.19737238468160	2.47764620525959	-0.38754317600283
C	-0.63517965031473	3.52053506329802	3.05760024372474
C	-0.36161957358089	3.35683063150248	4.41666592491820
H	-0.06456950487790	2.37923476100134	4.78547198621816
C	-0.44930958603331	4.44591044378245	5.27607343636233
H	-0.23261708358520	4.31609314886667	6.33228393228555
C	-0.80681774380755	5.69898138374735	4.78483529488298
H	-0.87432752577016	6.54778742765802	5.45900696225579
C	-1.07357176740037	5.86368967144969	3.42867134532880
H	-1.35089132119030	6.83980057684416	3.04182434077918
C	-0.98844370378522	4.77747736091773	2.56459547235907
H	-1.20442678740579	4.89399295642428	1.50549686066290
C	1.83421254670618	2.54771161910143	1.28339819034512

C	2.49120383670938	2.72181433533369	2.65975376739689
H	2.42685782504332	1.79070949289975	3.23185418695982
H	2.02208441752692	3.52315428881470	3.23581347625171
H	3.54782059944660	2.97809849341925	2.52728322995794
C	1.79952255593409	3.88556072569371	0.53100312626999
H	2.82117213879434	4.24709783717081	0.37019945543570
H	1.26146087491017	4.64473130136786	1.10629714258768
H	1.31071819660677	3.76796931673104	-0.44169299487454
C	-2.85031409308504	1.27369945383402	2.53752522615966
C	-3.58276603409529	0.24113909338216	1.67209300306939
H	-3.74915012571310	0.62787913220108	0.66198626416139
H	-4.55268977876824	-0.00195882975397	2.11636541728231
H	-2.99924176921061	-0.68408685601258	1.59158121047916
C	-2.69507616667825	0.71879375923926	3.96023500501909
H	-2.09029628589830	-0.19209487971795	3.95514923456714
H	-3.68232225008156	0.48721116712149	4.37535483316721
H	-2.21638290003541	1.44879582762048	4.61858180663099
C	-3.66694918702444	2.57230748678962	2.56839473904591
H	-3.23567368422581	3.30955711234969	3.25002958381756
H	-4.68305839890211	2.34991066133916	2.91060091099541
H	-3.73161243642221	3.01692172390044	1.56911433365920
C	-0.60629579399260	-1.61541806638501	-5.16970880763457
C	-0.22262640656775	-0.62859079715762	-6.07932128394088
H	0.02822144488307	0.36369442186917	-5.71268068365755
C	-0.15646909952467	-0.91978036457688	-7.43720893576983
H	0.14294491697516	-0.14788794324996	-8.13998411371448
C	-0.46913364946843	-2.19723638205005	-7.89357342513681
H	-0.41418962302257	-2.42467155098419	-8.95412010445522
C	-0.85476096959817	-3.18258791013450	-6.98871531193790
H	-1.10434266616372	-4.17903059982891	-7.34132446681679
C	-0.92567294889103	-2.89358591552343	-5.63050328807005
H	-1.23941007805877	-3.65558331805889	-4.92244502654467
C	-3.06160758572749	-0.49922689173778	-3.46753132304959
C	-3.77101268074616	-1.65506465491674	-4.18739239950486
H	-3.30309655936011	-1.88006019705186	-5.14904960679682
H	-4.81556185042857	-1.38497001621899	-4.37723633589610
H	-3.75482570913988	-2.55808745275529	-3.56748753371664
C	-2.99884063843248	0.73744641424582	-4.37541274829202
H	-2.45639137532150	1.54986367725549	-3.88120458780165
H	-4.01424937894736	1.08124998348686	-4.60174893451930
H	-2.50351061168067	0.50756812706954	-5.32298798458239
C	-3.84353955905609	-0.14838304827305	-2.19753972888589
H	-3.87535137043114	-1.00269888008217	-1.51177758862873
H	-4.86994179463386	0.13044742013891	-2.45442442098777
H	-3.37250625618456	0.69563955014832	-1.68500988234720
C	1.64312788321125	-2.12993490724223	-2.96600156806351
C	2.41193099732978	-1.81555478029951	-1.67612419251697
H	2.60524877310802	-0.74112027294574	-1.59338214891681
H	3.37089409416489	-2.34256497183066	-1.67109163004876
H	1.84900496914599	-2.13821220815710	-0.79286679880428

C	1.45234287277192	-3.64897781589106	-3.08703818961484
H	0.82880068777866	-4.01470844851152	-2.26606429030210
H	2.42188163826883	-4.15687046341731	-3.03513571405196
H	0.98207770699266	-3.91164779250450	-4.03938501129774
C	2.44462054819815	-1.60602481869458	-4.16521022717762
H	1.99524406756766	-1.89305933765368	-5.11913459847916
H	3.45635644330707	-2.02365313451304	-4.12907429184751
H	2.52626812141597	-0.51390891215466	-4.13211929236429
C	1.75565070124860	-2.22065808982185	3.66248080395550
C	1.78960650501451	-3.13664070049645	2.50993892328744
C	2.96573312280018	-3.83304006294159	2.18552547764390
H	3.86340660940471	-3.66082164652063	2.77276118170245
C	2.98415741972533	-4.74257018545640	1.13702032042802
H	3.90050874360224	-5.28127432443509	0.91030492294355
C	1.83735689648347	-4.96296061530689	0.37596411818066
H	1.85247528946147	-5.67995592792927	-0.44001788293947
C	0.67840414801511	-4.24797667291323	0.66334342637209
H	-0.21776617387404	-4.38472200823393	0.06470882114655
C	0.65131189429382	-3.34259624413678	1.71785947944330
H	-0.26293998481982	-2.79962625054088	1.93507486305809
C	2.40827163575687	-2.49863620526364	4.94589020211817
C	2.71190195713074	-3.81355789088978	5.33661745261904
H	2.46724757655436	-4.63734480795444	4.67302343830907
C	3.29646948967179	-4.07350208252124	6.56942745512307
H	3.51298992547219	-5.10106849164635	6.84901818280212
C	3.58948289629353	-3.03627786648213	7.45091305080086
H	4.04507533139799	-3.24354820435514	8.41445678779938
C	3.28765946278727	-1.72785883880795	7.07791013679260
H	3.51318752983459	-0.90528411866845	7.75155571547761
C	2.71233460546451	-1.46047601319836	5.84443200066570
H	2.49465157628418	-0.43403989129752	5.56117410737556
C	2.64214757540179	1.53078573718117	0.46989288430596
H	2.19094996441200	1.38883666947723	-0.51690178677893
H	2.68099767129501	0.56223050867976	0.98270544436440
H	3.66650164433309	1.89034457919037	0.33344789398685

TS4

Si	0.10903895198118	1.43093905782848	0.74750382352263
Si	-0.16508570891128	-0.37075658963055	-1.03783520016405
N	0.98174561299876	2.93142227118144	1.16507912394309
N	-0.58353747193937	1.92792281132738	2.29780485426360
N	-1.41448592692416	-1.09606362697526	-2.14905679317812
N	0.73080305984790	-1.35041582128847	-2.23273567514891
N	-0.55351396148987	1.35845102301734	-0.86503707875552
N	0.68863897275855	-0.19855756365287	0.50072329265622
N	-0.18757232828651	-1.28236167851520	1.00686570110454
C	0.27800409986288	3.01841343725288	2.36442474654321
C	-0.44187557469451	-1.76918705148121	-2.83969955055164
C	-1.17385912371140	2.31375877373972	-1.67896779792543

C	-1.28427572585555	2.11688879375925	-3.06235119778936
H	-0.88898565916049	1.20587396087843	-3.50354748311854
C	-1.89878096567918	3.07368505282643	-3.86043939511947
H	-1.97651941153340	2.90328415761573	-4.93082010997981
C	-2.40509267933063	4.24549847644236	-3.30339237659935
H	-2.88243473515642	4.99106662836984	-3.93156414034547
C	-2.28912213132220	4.44992313666313	-1.93114580107738
H	-2.67750589812057	5.35968320700264	-1.48176048120699
C	-1.68548040199358	3.49499646630683	-1.12252541567795
H	-1.60089485845638	3.66047028735435	-0.05075823486996
C	0.48090118640657	3.93730785510415	3.44660264222492
C	0.60867654115452	3.48544039367302	4.78172936414179
H	0.63593633161176	2.41938351946039	4.97795297915351
C	0.78420185020326	4.38522147067242	5.81874497029498
H	0.90461723932903	4.01206299517848	6.83233552722348
C	0.83530593391581	5.75946857007021	5.57232059432404
H	0.97073414046595	6.46009719394142	6.38981664172595
C	0.70500844763864	6.21985789431589	4.25906288382721
H	0.71578314691740	7.28739980599834	4.05597316495329
C	0.52864979554389	5.33178116531749	3.21233845534592
H	0.35709743516793	5.70343711586393	2.20766164485137
C	2.38804841905102	3.35209081084568	0.93027973909634
C	3.27437468507184	3.05543277692347	2.14584334913544
H	3.19680204619599	1.99787464092700	2.42439547984069
H	3.00144348616169	3.66601078066418	3.00987689989018
H	4.31923908426138	3.26764530213261	1.89466500995693
C	2.47528374122724	4.83621971775818	0.54956029901405
H	3.47378279078277	5.05212127652106	0.15495328501584
H	2.31430194051698	5.48443169124961	1.41249226084048
H	1.73814592711697	5.07718466723453	-0.22366289531199
C	-1.87370817948448	1.70910739281209	2.99852928764582
C	-2.74897949980071	0.92186434014967	2.02433181787348
H	-2.92783315502645	1.49215441182442	1.10573937918789
H	-3.71707922600957	0.69237347069169	2.48069038833311
H	-2.25550945883764	-0.02354487363919	1.77385260881731
C	-1.66856504315696	0.84718185973605	4.25280264910402
H	-1.11122564577690	-0.06141060704080	4.00104551250854
H	-2.64175204253207	0.54972765652777	4.65767975566803
H	-1.13584480134185	1.39062689966192	5.03547726788666
C	-2.55804110680905	3.03394038847379	3.34825042742528
H	-2.00767636215624	3.60267443018465	4.10114762411437
H	-3.55615514802016	2.82144882413176	3.74505849136637
H	-2.67074730611415	3.65932489260612	2.45549765469407
C	-0.58197710697280	-2.87737107163928	-3.77837237271617
C	-1.16650328065586	-2.70588511794626	-5.04416175823151
H	-1.47258232420207	-1.71231948601955	-5.35721723245638
C	-1.30561042960217	-3.78261856539255	-5.90978081276911
H	-1.74925934033795	-3.62895186462542	-6.88943352150470
C	-0.86185778497250	-5.04962878499081	-5.53552609826661
H	-0.97253680287220	-5.89021315579330	-6.21367947760125

C	-0.27123342860282	-5.22938844107877	-4.28409955054352
H	0.06428152309758	-6.21628700466162	-3.97787185569681
C	-0.12801256862832	-4.15869512438986	-3.41543598169435
H	0.29904088181019	-4.30566718760027	-2.42768828656148
C	-2.85522455314275	-1.42766734761489	-2.06516019435112
C	-3.08151307454105	-2.92717435349849	-1.82668881285110
H	-2.83577250930170	-3.52484888494062	-2.70788133445945
H	-4.13602162760796	-3.10065644775863	-1.58576863758620
H	-2.47459393485096	-3.27860909014160	-0.98507059022567
C	-3.58581999212585	-0.96761616167719	-3.33470039190748
H	-3.37974292676229	0.08942847585897	-3.53324033365927
H	-4.66515397157451	-1.08927375705397	-3.19441420230234
H	-3.29660904182693	-1.56112101144105	-4.20354107397794
C	-3.40995474455722	-0.63753685099002	-0.88232262412329
H	-2.92194725052349	-0.95083553506657	0.04287133774321
H	-4.48490216908516	-0.81614073677651	-0.78475962442559
H	-3.25001370692537	0.43639715984324	-1.02279979963621
C	2.07294961358204	-1.31423943016701	-2.86770627312902
C	2.96427503987500	-0.45594591245893	-1.97558637888199
H	2.59096776737703	0.57070860384298	-1.95324770466590
H	3.98159904242319	-0.43605608160741	-2.37815145274961
H	2.99762883908419	-0.84516540004497	-0.95286385767404
C	2.69865377247405	-2.71061973402709	-2.98091855221670
H	2.68603886032865	-3.21129935456916	-2.00844187291864
H	3.74202641527953	-2.60788935647837	-3.29784002649676
H	2.18558339216240	-3.33396869361939	-3.71545869228580
C	1.97209765843335	-0.67018136688462	-4.25720411804301
H	1.35361311699105	-1.27110855703911	-4.93090397663828
H	2.97011591379324	-0.58201853829205	-4.69995142277902
H	1.53973814408292	0.33451376845715	-4.18411271370833
C	0.45736934438958	-2.34013729839952	1.50224674033806
C	1.92990370685305	-2.44335980631511	1.47739203575426
C	2.74377475820154	-1.46049229539385	2.05869349381938
H	2.27810183253979	-0.64242706944840	2.59903813585018
C	4.12712054243714	-1.53371502359994	1.95752103972017
H	4.74148790320275	-0.76458898365793	2.41820756795073
C	4.72859247001427	-2.58885300133941	1.27375331397092
H	5.81053860355974	-2.64334802128225	1.19433139190022
C	3.93295583542989	-3.57884496650376	0.70387977214525
H	4.39128094528489	-4.40715809015016	0.17021416254773
C	2.54797615771850	-3.50688000515227	0.80684726460595
H	1.92736690500119	-4.27633907054435	0.35407160802181
C	-0.35684055271307	-3.43542183449467	1.99815318874558
C	0.21491755917021	-4.52606185255075	2.68970675437797
H	1.28681090995297	-4.54786063995878	2.85825872588291
C	-0.57279766831851	-5.55649365731410	3.17908674564016
H	-0.10206344987857	-6.37879104814207	3.71128159658031
C	-1.95743956684098	-5.54000939812052	3.00722030511895
H	-2.57096248376276	-6.34832222270398	3.39335833059830
C	-2.54141128203564	-4.46212463457639	2.34117431160461

H	-3.61971422685451	-4.42692204626738	2.20738818204618
C	-1.76332406080103	-3.42923435643958	1.84553453597634
H	-2.23196552765739	-2.59494699695104	1.33776979021888
C	2.86985566656023	2.51543009030273	-0.25354208455442
H	2.22194464005145	2.67269664142671	-1.12441623257190
H	2.87041194579950	1.45272900119072	0.01043257310562
H	3.88991635301510	2.79655304467446	-0.53228294809736

TS5

Si	0.20269594301582	1.69440138542686	0.81093043834891
Si	-0.10673614150557	-0.67104668700769	-1.00892908077869
N	1.64846106000367	2.44454359121379	1.67846144186882
N	0.06137388751024	3.53125275723872	0.71815482690327
N	-1.56613285125916	-0.58462022499074	-2.23834982234356
N	0.46283099586705	-1.03691920576201	-2.78302349626531
N	0.46341644043960	1.02996957216463	-0.75361003503224
N	-0.89136295817286	0.76007485565881	1.63503050429132
N	-1.11587349880042	1.12750883549097	2.92495115592907
N	1.73890010452259	-1.95949707267250	0.41142049141578
N	1.84262427459395	-3.11089562128019	0.40373010112344
C	1.15750758309324	3.67592495228674	1.47064467502664
C	-0.80617414271341	-1.11081263511211	-3.21129847206402
C	0.99723475234173	1.86774783744718	-1.76814663391914
C	2.24490022058930	2.48443830653999	-1.59731710331629
H	2.80885422845987	2.28407869287990	-0.69058153556625
C	2.77325701424018	3.30961998852162	-2.58332451571751
H	3.74739538851337	3.76814423747290	-2.43207457674867
C	2.07753153549747	3.52832102125331	-3.76958206219726
H	2.49411673145847	4.16594446729442	-4.54324062412438
C	0.84165716577064	2.91276807078254	-3.95102014818053
H	0.28377951843449	3.07659364495660	-4.86949034660863
C	0.30142482481052	2.09792384073240	-2.96229013772986
H	-0.66981794192359	1.63442183368034	-3.10049873319999
C	1.76828659311702	4.94947762025940	1.91737601893989
C	1.46799997222470	5.50847375678263	3.15935060975452
H	0.74676874467035	5.02244636167006	3.80990975650802
C	2.08532400551088	6.69245942014884	3.54873556269844
H	1.84995938540718	7.12722572531929	4.51539881866767
C	2.99863520572196	7.31753156781822	2.70431469587154
H	3.48055049852708	8.24121726776574	3.01142968973879
C	3.29260516142972	6.76022321438168	1.46283459941718
H	4.00248527982418	7.24640197863580	0.80045961599663
C	2.67973841406114	5.57790645031208	1.06657145981930
H	2.90504437658097	5.13126675314862	0.10138346372536
C	2.66298546401798	1.95287481675691	2.63669935689927
C	2.16601351118195	2.13634939568418	4.07682140777612
H	1.15387642575141	1.73110352218100	4.18403362047421
H	2.15765885618348	3.19328965715888	4.36067552784149
H	2.83457745751280	1.61160640162705	4.76758552798659
C	4.00934941476456	2.65556500461379	2.43067479540054

H	4.76158480660825	2.18297300377976	3.07055225192266
H	3.96234033859265	3.71570494760087	2.69311297653313
H	4.34045354379841	2.56562785846037	1.39006024529312
C	-0.90486661444315	4.51610476161600	0.19563574408225
C	-2.03683119070749	3.68705109199183	-0.42039822562785
H	-1.64977721031802	3.05309891701940	-1.22489485664695
H	-2.79731740561344	4.35058611311574	-0.84289797989610
H	-2.50723495205013	3.04714452695915	0.33429624106651
C	-1.47090818803519	5.37989953015474	1.32939955633698
H	-1.85758873310001	4.74635850471264	2.13506608955369
H	-2.29377463629415	5.99155911059289	0.94504377899446
H	-0.71512864639539	6.05508363532455	1.73879664026057
C	-0.25845890498821	5.39037889793541	-0.88605586432997
H	0.52530820812122	6.02671230028744	-0.46516311508432
H	-1.01711330027498	6.04130638888777	-1.33384517785957
H	0.17652475231096	4.76837076573727	-1.67497749540621
C	-1.29544349128845	-1.77539922876307	-4.44558238930651
C	-1.62829739176060	-1.01808219105238	-5.56868566509793
H	-1.49102618289879	0.05984639973020	-5.54776895243183
C	-2.10808103001974	-1.64504892965838	-6.71351550262119
H	-2.36140855342301	-1.05254981276234	-7.58772797221172
C	-2.25990151421023	-3.02863557590636	-6.73900265985628
H	-2.63658169873523	-3.51720670194342	-7.63290325509758
C	-1.92915455214208	-3.78476835664877	-5.61721998520746
H	-2.05170987526735	-4.86399313514278	-5.62983286102868
C	-1.44645856520491	-3.16311749262195	-4.47180029114647
H	-1.21576048159932	-3.74848879452768	-3.58527877531487
C	-3.03435876697422	-0.47152888109021	-2.12275937744356
C	-3.79419963269490	-1.70748184241167	-2.62323777565172
H	-3.76249902183654	-1.81516486049943	-3.71004164352073
H	-4.84364574219227	-1.61871316033085	-2.32249557993777
H	-3.38589105614815	-2.61365977742249	-2.16773367161216
C	-3.50245127160596	0.76674700294563	-2.90304227993677
H	-3.02530080730520	1.67274686820514	-2.51613318690256
H	-4.58695115027527	0.88321102118866	-2.80132743819664
H	-3.27094995173573	0.67126947522005	-3.96924823617319
C	-3.33693160828993	-0.28169254290056	-0.63391029741037
H	-3.08730940342223	-1.18104199550263	-0.06117532418479
H	-4.40207984344000	-0.07175275357183	-0.49469028425956
H	-2.75741806649041	0.54040400384404	-0.20229794382022
C	1.70422046225964	-1.50028685356416	-3.43915204538294
C	2.86800487351532	-0.79916973199749	-2.72759444959435
H	2.85391749919768	0.27569310484387	-2.92595012835002
H	3.81688132745452	-1.20768030629679	-3.08938987570180
H	2.81800278589368	-0.95557723629052	-1.64485845252671
C	1.87282928452772	-3.01908179123339	-3.30237309505385
H	1.88519131401151	-3.31743994862356	-2.25128408759505
H	2.82039514890293	-3.32867537056258	-3.75735033696638
H	1.06601627764306	-3.55964419912465	-3.80395680817284
C	1.73431410608712	-1.09814616198268	-4.92018857535947

H	1.03260274866693	-1.67927559521157	-5.52253679839335
H	2.74097391825414	-1.27551264045207	-5.31305807792859
H	1.50449526876696	-0.03271368568383	-5.03231496914417
C	-2.01198588413156	0.46988226415368	3.60531496935724
C	-2.77919920786042	-0.68109734176258	3.05649936728740
C	-2.13646761289789	-1.85968281130188	2.66560729205053
H	-1.05343843115300	-1.91830835305915	2.72685861633673
C	-2.87606242241582	-2.95153734372912	2.22206625115668
H	-2.36983272716306	-3.87515431882320	1.95642854739677
C	-4.26355979389625	-2.87043801714960	2.12474642004254
H	-4.83704065658880	-3.72586758618996	1.77760409158979
C	-4.90995990850566	-1.69171473696005	2.48530764071502
H	-5.99176512645774	-1.61779814585072	2.41160664804707
C	-4.17223393086598	-0.61068979241283	2.95700409731821
H	-4.67716337226393	0.30147040698538	3.26630934316501
C	-2.27141730064161	0.90462118218472	4.98597088583474
C	-2.94404085276531	0.06683376458931	5.89134957214910
H	-3.27678642559940	-0.91305400369694	5.56252879800943
C	-3.17831545131251	0.46787459824621	7.20170238823468
H	-3.69675380209178	-0.20349958788467	7.88114265222044
C	-2.75052035893237	1.71430461272134	7.64677732023003
H	-2.93866710629148	2.02689087647488	8.67000324343974
C	-2.08097437245282	2.55760694843035	6.75857427194653
H	-1.74895484225352	3.53813433034151	7.09098804988084
C	-1.84723873523177	2.16385537238841	5.45013401367863
H	-1.33645799556678	2.82323314129484	4.75528566442877
C	1.74763883181883	-4.40943247370191	0.25391997056098
C	0.54609045700281	-4.91054929178535	-0.42754359302668
C	-0.68401796347484	-4.25328273687807	-0.27085252660433
H	-0.75162701239822	-3.37506025517611	0.36384683177101
C	-1.82253071960695	-4.72488493285394	-0.91292974634569
H	-2.77052545160766	-4.22145703691299	-0.74463027258327
C	-1.75903710867874	-5.84416404405839	-1.73894232363518
H	-2.65374790992827	-6.21607975368925	-2.23023559762894
C	-0.53450477929858	-6.48529424609188	-1.92308566627778
H	-0.46724298607143	-7.35333917109715	-2.57341394595408
C	0.60675999407476	-6.02604571474958	-1.27873007602327
H	1.55812157955771	-6.52511157670524	-1.43846602974040
C	2.84898142001899	-5.22927852816635	0.76465462457844
C	4.13013362680757	-4.68386470723542	0.95378937954401
H	4.31466110650508	-3.64698374063244	0.68476368899919
C	5.16142978669577	-5.45356463114808	1.47239007022120
H	6.14334872083973	-5.00874545359855	1.60879700819047
C	4.94950232193349	-6.78968245574108	1.80502773887795
H	5.75937964541613	-7.39226006653544	2.20471090002141
C	3.68431940997984	-7.34098911641275	1.62113124654023
H	3.49917489379460	-8.37747610088580	1.88955070419975
C	2.64381360638321	-6.57392355606858	1.11331840789492
H	1.65551584179517	-7.01026503772326	1.00497599652065
C	2.80608361175416	0.45880814203845	2.33526035974759

H	3.10862658388661	0.29367634969535	1.29622233917005
H	1.85543749417005	-0.06186038004115	2.49220942245617
H	3.55808119126301	0.01359102231118	2.99333834472686

TS6

Si	-0.33984341886931	0.78740980231837	1.11651366569426
Si	-0.38907093953246	-0.40965963991010	-1.45315727638310
N	0.93539459713254	-0.06718705258380	2.22116798675563
N	1.20989798084067	1.76064935186018	1.10750270615348
N	-1.57706805944934	-1.28245958868212	-2.55338776107351
N	0.07462887727943	-0.05627348319659	-3.18973908105905
N	-1.16347605545415	0.74115751082860	-0.47200361552907
N	-1.60510266628238	1.24085614345665	2.13246030334948
N	-1.25807573251264	1.55388326676821	3.39769531223611
N	0.73064884312790	-0.94972349922540	-0.38158994478232
N	2.03880535671724	-1.22948930027803	-0.49679033335756
C	1.77691718447207	0.92518287206591	2.02458184101925
C	-0.93357768032682	-0.83316622654291	-3.63418693432403
C	-2.33663192863952	1.44429738075519	-0.87300370333089
C	-2.32244857464279	2.12057569974102	-2.10006892478147
H	-1.40393745112124	2.14515033577700	-2.68168505418180
C	-3.46027324111711	2.77211497751700	-2.56741300280211
H	-3.42470730527967	3.29853632757345	-3.51799237989878
C	-4.62669846359672	2.77223052080412	-1.80924585741002
H	-5.51303545329241	3.28898100596454	-2.16574684648441
C	-4.63785899956691	2.11712099598074	-0.57955653672185
H	-5.53324278876851	2.11964883222715	0.03655830703288
C	-3.50997827902088	1.45284679471042	-0.11131235286488
H	-3.50481273544765	0.97696395239318	0.86270148236393
C	3.14395281396192	1.05130182260652	2.58753231103818
C	3.38846740424629	1.58880815379239	3.85075621386202
H	2.56041086012473	1.94141611798931	4.45828195957032
C	4.69312990896370	1.66342927608275	4.32889194550305
H	4.88067365899726	2.08499680587854	5.31200562657750
C	5.75133659317947	1.19303540080940	3.55667544541194
H	6.76719458049044	1.24793971601437	3.93730011824448
C	5.50399042883411	0.64286756896488	2.30130686937153
H	6.32138768494587	0.25505756476936	1.69980256750675
C	4.20516265957266	0.57450337818793	1.81362210385700
H	3.98866513146258	0.12381828564886	0.84917301610461
C	0.98397022315237	-1.25087504900331	3.09630905266583
C	1.01975514606652	-0.82030091476699	4.57044440682701
H	0.24130573811672	-0.07632204706515	4.76648764077385
H	1.99322736815058	-0.39832773658177	4.83475003064366
H	0.84941984980560	-1.69408343809968	5.20877040910525
C	2.18099918588434	-2.15393107372467	2.77788100331086
H	2.14277342623559	-3.03977301063349	3.42125442815032
H	3.12933477054240	-1.64240699517576	2.96392844210229
H	2.16662720747027	-2.47608956495755	1.73353888762480
C	1.43895158062857	3.21730191701854	0.95880046786158

C	0.33520739501585	3.73231127943924	0.02866737413828
H	0.35724321167865	3.21577138971749	-0.93489359453846
H	0.47204638758848	4.80345852325032	-0.14821129159636
H	-0.65511338846321	3.58115115202014	0.47436957220288
C	1.30427303063675	3.93500029671901	2.31018843635889
H	0.36890786923529	3.64511966258519	2.80060336555455
H	1.30369253161397	5.01987231103783	2.15770328966395
H	2.14002527487089	3.69368589613409	2.97221078022768
C	2.81256871267477	3.49415474261662	0.33749136385238
H	3.62302902978621	3.16256729115150	0.99249659057464
H	2.93160205912274	4.57013155452712	0.16892999599124
H	2.91161387474284	2.97818403754361	-0.62318023997258
C	-1.25772007959055	-1.13475578740297	-5.04612427709439
C	-2.08643744234264	-0.27315413736913	-5.76562798674101
H	-2.48611434713799	0.61521755731437	-5.28353602074493
C	-2.38865359380385	-0.55908765121118	-7.09168966200348
H	-3.03552795290180	0.10928441324005	-7.65171913256380
C	-1.86292612924639	-1.69633947247827	-7.69909009858267
H	-2.09951966207390	-1.91573376995983	-8.73595550267168
C	-1.03787049144471	-2.55439030471653	-6.97727310515188
H	-0.63245320664162	-3.44513772640277	-7.44764988132066
C	-0.73510844613138	-2.27973842932795	-5.64857805205709
H	-0.11023049443535	-2.95552488124690	-5.06969115506722
C	-2.86821847441703	-1.99586640941483	-2.40687447762244
C	-2.86099893829366	-3.31204358819781	-3.19194044757627
H	-2.87579925658767	-3.14213306408541	-4.27166948468496
H	-3.75724795968647	-3.88541675984839	-2.93313309469761
H	-1.98136752124685	-3.91139841365345	-2.93912655686557
C	-4.03135994143164	-1.10917577423330	-2.86860934264988
H	-4.04017101672300	-0.15480077139524	-2.33513897426623
H	-4.97699271565917	-1.62461154654985	-2.66972955090680
H	-3.97610246607434	-0.91667394410863	-3.94418230662089
C	-3.00518772148137	-2.29234528294404	-0.90906496590787
H	-2.16287350708363	-2.89745845611105	-0.55596231760055
H	-3.92972271643222	-2.84857288675916	-0.73021298530306
H	-3.04730400862317	-1.36681335972023	-0.32507465231351
C	1.24217332302995	0.51753160251604	-3.89614823233703
C	1.91715801642045	1.44947883093905	-2.88604343625938
H	1.25698980902279	2.28383290711254	-2.63021855546111
H	2.83500907312450	1.86090972566880	-3.31735778048655
H	2.17654175474654	0.91734400577303	-1.96356114968389
C	2.21876802699983	-0.58972795059388	-4.30685354150756
H	2.56895975683029	-1.13579031680356	-3.42646180275054
H	3.08872897091606	-0.15185129857950	-4.80774936314132
H	1.74768379205166	-1.29480535524616	-4.99822546590407
C	0.81315715736201	1.33020765028296	-5.12359896545967
H	0.42965433533988	0.69600715790057	-5.92618129154546
H	1.68176947214730	1.87301841428061	-5.50980186588830
H	0.04490722326716	2.06368639855627	-4.85652651563119
C	-2.21578618776553	1.88771264533322	4.22603829171268

C	-3.64671360261219	1.88324100520960	3.81872069918834
C	-4.27329916990526	0.69114749564929	3.44073195557585
H	-3.70225709954840	-0.23262975472135	3.46576670274322
C	-5.60356743145118	0.68383703401753	3.03751814447568
H	-6.07847382888233	-0.25295771538560	2.75661561560205
C	-6.33019580361828	1.87286579347178	2.99672460876573
H	-7.37091447110308	1.86751717652215	2.68351650255150
C	-5.71594375667758	3.06534468805690	3.36781931381892
H	-6.27451759018620	3.99723334853223	3.33970872725469
C	-4.38533406273539	3.06796336100209	3.77735786791505
H	-3.90575523999833	3.99916453819880	4.06913652654571
C	-1.84314575517085	2.28577160960644	5.58523572395311
C	-2.80594511899087	2.37333476952557	6.60665634691325
H	-3.84316683436021	2.14546656468515	6.37885958517980
C	-2.44903722309393	2.73390032709230	7.90097017758105
H	-3.21522542536758	2.78853442868461	8.67006288256269
C	-1.12441881600950	3.01822213940518	8.21814518391938
H	-0.84802507488823	3.30303415143265	9.22930106658353
C	-0.15740274249385	2.93904986969851	7.21418207446841
H	0.87999385155218	3.17044643940833	7.44414390946951
C	-0.50766475605993	2.58487361354268	5.92139910304764
H	0.24037684153836	2.53771313284271	5.13626747457914
C	2.47121159592678	-2.31258175734461	-1.07541001827689
C	1.62168762920333	-3.41079356913234	-1.60575216878939
C	0.60562398442629	-3.97290429009534	-0.82234243743904
H	0.40508827786150	-3.55851283064116	0.15997917758004
C	-0.11283800820320	-5.07162739823717	-1.27690362206014
H	-0.88106202473260	-5.51022022611743	-0.64556100987354
C	0.15018196394912	-5.61576203990427	-2.53278636110711
H	-0.41368732312592	-6.47472989623348	-2.88564590673486
C	1.14809813619900	-5.05791999677610	-3.32620794118231
H	1.37273340329310	-5.48543316390336	-4.30000726384903
C	1.88314575174102	-3.97085024220067	-2.86223901088777
H	2.68992259317208	-3.56473526900035	-3.46690969413578
C	3.93993623222644	-2.45520397548295	-1.16711174977056
C	4.75860330365336	-1.32843140279087	-1.33467868616377
H	4.29154519145140	-0.35476398801130	-1.44956441641590
C	6.14149302291175	-1.45388114330810	-1.37157052949766
H	6.75891002657586	-0.56990595140559	-1.50845851305881
C	6.73661481158014	-2.70805514679464	-1.25587025523056
H	7.81771909958048	-2.80676394018573	-1.29026071031878
C	5.93368191582334	-3.83630276851398	-1.10908269220452
H	6.38844498170900	-4.81863052499430	-1.01707582284162
C	4.55009150644835	-3.71370092027915	-1.07155460278014
H	3.93120929529838	-4.59795431274998	-0.94670435310621
C	-0.32549547764157	-2.00717306950568	2.83534874830811
H	-0.42030157920147	-2.24954985807332	1.77091874741854
H	-1.18410851324937	-1.39410240083217	3.12822842768693
H	-0.33990839680106	-2.93688919934057	3.41253558920442

3-like

Si	0.10378276628804	4.39645014699814	-5.12675931734532
N	-1.70602751782083	3.40550844644740	-0.93651847986486
C	-2.48904771744082	3.28532935839300	-3.91168524397170
B	-3.01637708270064	4.30723196834652	-5.17989698577380
H	-2.92539550932072	5.46637210892693	-5.04165792075006
Si	-1.69523034664828	4.30980466078133	-2.55233118318057
N	-2.82142191467756	5.19361714736938	-1.43524934317651
C	-1.57311678358672	3.37618698494537	-5.30830748834294
B	-1.69224360876724	1.87911542394580	-4.47905079028275
H	-0.72505894483908	1.49410141228695	-3.92675921626985
N	0.86388708793580	3.75777334648975	-6.91469158221884
C	0.95719245006234	5.02414714577101	-7.23628583609419
B	-1.83099201556394	1.95384376528800	-6.23236150051375
H	-0.94923055396674	1.52354161050294	-6.87596408013341
N	0.24703397290932	5.77552120846389	-6.33584320109159
C	1.79518122875393	5.58346265153161	-8.32740981695301
B	-4.15957947572799	3.32824932556523	-4.24221653636655
H	-4.85682305630351	3.88092161501335	-3.45530447590654
C	3.01045990969980	6.18535087202375	-8.00032051967098
H	3.32582626661245	6.22021413103192	-6.96156705866965
B	-2.62076951469084	3.46167630477651	-6.66898310699323
H	-2.25551433530437	4.10084831975245	-7.59847080083245
C	3.83868527011463	6.66342338592173	-9.00762150947419
H	4.79454164758035	7.10742020321994	-8.74439618270594
B	-3.34120938606806	1.82433438178062	-3.80687965579332
H	-3.51227253172226	1.35245940872969	-2.73468221217891
C	3.45390687942037	6.55271534548084	-10.34156032509789
H	4.10248579675693	6.92664983012818	-11.12865824978453
B	-2.95604912704926	0.95166014589612	-5.29913183992990
H	-2.89205624347140	-0.23410371080454	-5.32301475097670
C	2.23952772734937	5.95309903581702	-10.66697175608169
H	1.93602584718348	5.86512445099556	-11.70610812935542
B	-3.54440103357644	1.93600628957800	-6.66770365196598
H	-3.90336440889368	1.43963129447750	-7.68580609690614
C	1.41291296945803	5.46215965371548	-9.66293437101319
H	0.45852455414601	5.00682846921114	-9.91162333567095
B	-4.27872581131611	3.41492818819627	-6.01130589078087
H	-5.16791171443482	4.00703914399515	-6.53146113756355
C	1.71785086902928	2.64518907842432	-7.44045792814174
B	-4.48778587840561	1.85443505464936	-5.16320812386554
H	-5.54055856512059	1.30912433281234	-5.08142912568144
C	1.78980004024219	1.53992478493750	-6.37459028255158
H	2.48126483768577	1.82576376234000	-5.58084784794637
H	2.16516415197614	0.62487640213198	-6.84479494307918
H	0.82243451659210	1.31843978775960	-5.92371171361813
C	3.17735371796574	3.03522930487995	-7.74175094810971
H	3.28645628396443	3.59820445933837	-8.67004893930569

H	3.75567829199320	2.11056636548781	-7.84652579068785
H	3.60527063807864	3.60988104010489	-6.91567140276443
C	1.08220195186219	2.09777358112959	-8.72962463949895
H	0.04120918506339	1.80209702590135	-8.58172374253536
H	1.64707107079616	1.22213626306379	-9.06760353976410
H	1.12068521327304	2.84641598209134	-9.52538720647149
C	-0.07062763084923	7.23229440319468	-6.48968681622901
C	-1.44415909921503	7.49785395399850	-5.86960218726270
H	-2.22588611033970	7.00080816289195	-6.45284768213224
H	-1.63982637349183	8.57550776581221	-5.88059728675032
H	-1.47748340095802	7.15471652120491	-4.84088950413485
C	-0.19829967815713	7.66694135727524	-7.96182265593256
H	0.75990398350244	7.77449906960347	-8.46880029001239
H	-0.69046786605078	8.64492944426435	-7.97382150006279
H	-0.82538486249299	6.96948250559950	-8.52755223442107
C	1.01098017207051	8.08023665479268	-5.80805792853903
H	1.14775121734180	7.77257602421068	-4.77019048712982
H	0.72425684591948	9.13807413717334	-5.82211331848327
H	1.96289618226669	7.98011914996031	-6.33684505952018
C	-2.59271682054367	4.28667155731255	-0.46180490754557
C	-3.24539160257194	4.25855128293618	0.86682451725353
C	-2.71391831277758	4.98583949119731	1.93196430440641
H	-1.78880370630143	5.54011980128575	1.79906345636078
C	-3.35482582628752	4.96301484207086	3.16508237116245
H	-2.93883013557501	5.52458931656531	3.99590917136802
C	-4.51866254653630	4.21872449892288	3.33636100215099
H	-5.01664813127130	4.20454155353892	4.30114831064356
C	-5.04221554426064	3.48893073713011	2.27281763367761
H	-5.94868165401608	2.90600740799206	2.40316985909770
C	-4.40829684707608	3.50585085319921	1.03639829960285
H	-4.81907835800570	2.94999529123204	0.19799411485595
C	-1.09217161694458	2.22947117089021	-0.24398758067099
C	-2.11774669577807	1.11079286992464	-0.03185031501206
H	-2.90482733465811	1.40848677825285	0.66608478852025
H	-1.60183201113677	0.24582282336667	0.39801811308874
H	-2.56958832998268	0.80022587077223	-0.97631844128715
C	-0.50953558301202	2.65527690953323	1.10820459347770
H	0.09969752641781	3.55408341919280	1.00336220468775
H	0.13288143589966	1.85243554373459	1.48228344249185
H	-1.28880724786131	2.83170090047433	1.85251219241431
C	0.02872015492695	1.72443131333731	-1.14933139301283
H	-0.37475072797515	1.22932612331238	-2.03432131212858
H	0.63083470085595	0.99390748060537	-0.60336265298432
H	0.69334283625924	2.53009601054820	-1.47186319781684
C	-3.63948010442549	6.44073149973750	-1.38551007253792
C	-2.94261111015067	7.48238250520732	-0.50237142191486
H	-2.89796843793706	7.16170147068965	0.54150655911960
H	-3.50295524266856	8.42228062257804	-0.53964998168745
H	-1.92868516329829	7.67625441428451	-0.86454216930495
C	-5.06047049552335	6.16571168019346	-0.87886293138609

H	-5.51739005930464	5.34035802114835	-1.43440628114706
H	-5.66668576808109	7.06183069638213	-1.04409545461727
H	-5.08816871067799	5.93673062408582	0.18807132097253
C	-3.72155016014825	6.98006888512790	-2.81146883571004
H	-2.72257345930363	7.14663753729068	-3.22148043851603
H	-4.24849402816112	7.93852379173987	-2.80117487159631
H	-4.26889292387083	6.29682885069169	-3.46523250925586
C	1.41220416815427	5.84758306807080	1.43002649408851
C	2.29218644066711	4.79689778395723	1.17074657537916
C	2.44014638010502	4.32043267309577	-0.12795102995539
H	3.11857888259161	3.49842587666810	-0.33994927482025
C	1.74047590492181	4.90896953999095	-1.17854165005820
C	0.89330836703696	5.99629144308469	-0.93476517852586
C	0.41139935240437	6.83207263915218	-2.06558740085824
C	0.71638773678458	8.28729775465817	-1.98590075428820
C	-0.13070324258472	9.22685035422523	-2.58761045942180
H	-1.03274203993099	8.88145930096353	-3.08221317744789
C	0.18083762353293	10.57961816099158	-2.56244719984443
H	-0.49032510551203	11.29548304167438	-3.02875172479607
C	1.34682382079970	11.01879744173209	-1.94055870499535
H	1.59128487551591	12.07696296102366	-1.92219202646634
C	2.19818745336682	10.09328214477158	-1.34339237001304
H	3.11704155457085	10.42545108840942	-0.86902120858358
C	1.88421612028642	8.73985655789300	-1.35772790337866
H	2.56000402855487	8.02302856354512	-0.90106765666638
N	-0.15139067968868	6.47983880004367	-3.17429535223665
N	-0.61779970258158	5.21649736268564	-3.46396222205976
H	1.86793580903536	4.54794705243868	-2.19727737082372
H	2.85759938137064	4.34741949062269	1.98254629225631
H	1.28791440382037	6.21882860551799	2.44399116912628
C	0.72351677303127	6.44842456680353	0.38393798420977
H	0.09474332826567	7.31488312182268	0.57517145832226
C	5.43960889482622	2.20291214557397	-1.21361390650123
C	4.60192337529063	1.10637819665148	-1.00959328706353
C	3.47216918108875	0.96912102562635	-1.81229033535730
H	2.81363767423835	0.11442902113632	-1.67613481185832
C	3.17218422168720	1.90313466541426	-2.79771102933707
C	4.01718244209696	3.00826250384393	-3.03246916541778
C	3.75756983081160	4.01199793608165	-4.07173825153116
C	4.87774386477092	4.81787377726222	-4.60643955712302
C	4.72151614920312	6.19903632952739	-4.80275103997627
H	3.78853610216168	6.66114817109522	-4.49073070000299
C	5.73259913366383	6.96252240298527	-5.37592306853429
H	5.58872391611985	8.03234369892433	-5.50926695162201
C	6.93168846414493	6.36663949518014	-5.76133876126580
H	7.72471143166382	6.96179714869013	-6.20547186324423
C	7.10717799647404	4.99862164933540	-5.55927988437099
H	8.03520610989496	4.52029946265058	-5.86155793337629
C	6.09648046248349	4.23566206947526	-4.98668707504102
H	6.23622853041700	3.16631706345095	-4.84863648808143

N	2.59463311484588	4.33494050933557	-4.62180126486838
N	1.44545948934334	3.79900117395080	-4.22657652737378
H	2.27839499254340	1.78851142830459	-3.39712403317219
H	4.82947731370968	0.37183079906570	-0.24224971254372
H	6.32267666549511	2.33683888531763	-0.59379482689571
C	5.15325718366198	3.13253714024569	-2.20263089325780
H	5.81208095068550	3.98482454050395	-2.33896092221808

1-like

Si	0.08492762371240	4.60096790192558	-5.07584807365608
N	-1.17897357356227	2.98007165996456	-1.25023884383769
C	-2.31300864647532	3.51063207528896	-3.97802401428900
B	-3.03899529609583	4.76874279080579	-4.88255532162422
H	-2.88158541479577	5.88115735722519	-4.54289115313751
Si	-0.98506583088967	4.16891128475915	-2.70358838549878
N	-2.13955751253307	4.92232901154421	-1.32620507484360
C	-1.69770949805565	3.85317609758391	-5.46500574163757
B	-1.77262618407173	2.21195649730373	-4.95727087315755
H	-0.79075012335968	1.65393865052940	-4.66075595353880
N	1.06927041389464	3.79844946171661	-6.55560583570149
C	1.04419917117416	4.95875664864634	-7.20348738966263
B	-2.17128438879381	2.66860177002121	-6.60806021158576
H	-1.41538916340244	2.39519665553380	-7.48067437704961
N	0.37269138431554	5.85291251651809	-6.45885241927311
C	1.70066138517174	5.24194118932825	-8.50852008328347
B	-4.02772905450956	3.62721474725009	-3.97702981777308
H	-4.57142860591037	3.97338271808344	-2.98878302177479
C	2.95995883145882	5.83983435999436	-8.51534171959871
H	3.44551165924789	6.08587310026240	-7.57403823868617
B	-2.95128009266807	4.24687428757339	-6.56300178716014
H	-2.73896163266169	5.04633334784281	-7.41377240646642
C	3.59145145714082	6.11413592905646	-9.72535819529950
H	4.57424479052906	6.57611628941137	-9.72501542987943
B	-3.24688302394545	2.05939544366515	-4.01833103419748
H	-3.25414447231348	1.36425464376048	-3.05685378530233
C	2.96572400451415	5.79915082703776	-10.92654550358747
H	3.45727811503575	6.01746673892679	-11.87007540406953
B	-3.18827825288663	1.51321799842328	-5.71193024165811
H	-3.19058345857730	0.35581098609429	-5.98599869035117
C	1.70642905906829	5.20186207525333	-10.91821773858567
H	1.21373770603995	4.95480310355789	-11.85380662029711
B	-3.94013201577349	2.77523504012680	-6.71711376466595
H	-4.50875900803914	2.52245403410112	-7.73086922746103
C	1.07642338155522	4.91975111845426	-9.71467030230692
H	0.09424026929201	4.45609735209297	-9.70410397596981

B	-4.46786842268228	4.08840948641630	-5.63846024720218
H	-5.40168431499732	4.79156470842075	-5.85736252264823
C	1.89355655192836	2.60143313654277	-6.90310502194515
B	-4.60795110864542	2.39536700534921	-5.10995773815923
H	-5.66316029845228	1.87116698522841	-4.94630163603499
C	1.39781635077403	1.41822448639458	-6.07087349197175
H	1.28243748746907	1.69438241291179	-5.02207031121269
H	2.11970993846256	0.59908746781297	-6.15495734030292
H	0.43181245899066	1.06107941126066	-6.43622859908779
C	3.37452602998707	2.85302023380357	-6.58354282601715
H	3.75366869812880	3.73674688723412	-7.10373599161825
H	3.96643287364386	1.99007698917940	-6.90820817782800
H	3.53142997214638	2.98903834376350	-5.51250182892794
C	1.76615987151300	2.19006569762225	-8.37976910673243
H	0.71844455899335	2.18588478691368	-8.69473185727519
H	2.15130218266959	1.16971497345913	-8.48108740372656
H	2.34159126801195	2.82893642396736	-9.05025826344348
C	-0.04806548605468	7.23036325587351	-6.88005386994444
C	-1.28851003597340	7.64282100794676	-6.08279808545745
H	-2.14858139612055	7.03111503759325	-6.36388670389105
H	-1.52235541242230	8.68496803004038	-6.32488320829776
H	-1.12275033963913	7.56451359184421	-5.01086843102566
C	-0.44584185047955	7.31152474780668	-8.36429037969725
H	0.40918020639742	7.30693516412639	-9.04061636596487
H	-0.97814868550165	8.25613479955743	-8.51508584111030
H	-1.12753681966478	6.49953779642462	-8.63689695920727
C	1.09349385720379	8.21781222149676	-6.61278584771947
H	1.34589957912370	8.23809465109725	-5.55152769258659
H	0.78304363839989	9.22683049336291	-6.90712153014941
H	1.98195139922171	7.95577248213931	-7.19525832468161
C	-2.02609664761376	3.80679917289404	-0.61249799413943
C	-2.73204272108313	3.49876140535922	0.65867091627166
C	-2.09168883178441	3.69673388483932	1.88172234706114
H	-1.09487567567301	4.12802174898488	1.90180527712778
C	-2.74674539681020	3.37690915897907	3.06493094696449
H	-2.24799807285592	3.53752173936744	4.01615959222711
C	-4.03800711150855	2.85735807517655	3.03151420021050
H	-4.54702591043320	2.60506222416976	3.95709283250869
C	-4.67676161031314	2.66309158319439	1.81006791863567
H	-5.68239944013923	2.25476418269406	1.77864122238901
C	-4.02756256547363	2.98350724851179	0.62372703935264
H	-4.51027298787351	2.80330008977203	-0.33303766317631
C	-0.71788044959823	1.62175950525731	-0.81874271768342
C	-1.81125809794894	0.79715657526548	-0.11912433399070

H	-2.03545848351530	1.14168065743767	0.89098298529097
H	-1.44886101288113	-0.23330624408225	-0.04463878799099
H	-2.73424056962872	0.78391154372316	-0.70638580929986
C	0.47996949611534	1.77947862170447	0.12422320284475
H	1.28981899959810	2.32805605801493	-0.36527820437270
H	0.86383091667953	0.79310929338480	0.40641586631441
H	0.18714561744817	2.30272578578325	1.03948637939843
C	-0.28341959488328	0.81823424726129	-2.04706063324941
H	-1.14220215728149	0.57355854128608	-2.67600046008311
H	0.16679075636868	-0.11845208968811	-1.70297160245074
H	0.44447148434896	1.36550370473615	-2.64381895427759
C	-2.93786030332959	6.14001051401736	-0.96234636894137
C	-2.67729689690460	6.55702144916042	0.49583482337975
H	-3.24194025181522	5.94987780796881	1.20601137887857
H	-2.99587113687382	7.59719322497070	0.62243855185364
H	-1.61426513431652	6.49763765503151	0.74141680757236
C	-4.44762403687230	5.93162319979938	-1.15156601559837
H	-4.70823572848166	5.84703554124571	-2.20789650512482
H	-4.96713788690563	6.80522920419255	-0.74266439046983
H	-4.81338654207526	5.05050096714720	-0.62118421828247
C	-2.52776637014203	7.30073665861021	-1.87156152627199
H	-1.51292090813433	7.63890908171487	-1.66586746009656
H	-3.20722310357355	8.13769145609366	-1.68221666038718
H	-2.60270310804442	7.03158004815041	-2.92572016898896
C	0.83124352334649	7.45943209334340	1.38677607371270
C	1.12837806992282	6.14627929001638	1.74458535028274
C	1.24184072455808	5.18047234986411	0.74979051597361
H	1.51040631306691	4.16224857814589	1.00984545584994
C	1.06039922977702	5.51420531349843	-0.58929843759535
C	0.78372337306107	6.83230767668008	-0.96896392111986
C	0.75733700431222	7.29482789254218	-2.37625432204295
C	1.45275712693107	8.58823706607863	-2.62438696372199
C	0.86758366462363	9.57744956226011	-3.42189880430319
H	-0.12423395230312	9.40181670874532	-3.82769318399993
C	1.53935328386860	10.76606457935654	-3.69063668668685
H	1.06540796495637	11.52424578776485	-4.30833219073667
C	2.80744111058560	10.98923291652143	-3.16309918069487
H	3.33194465741454	11.91748072768526	-3.37103152649326
C	3.39542119048957	10.01655710313779	-2.35563874631444
H	4.38782493106594	10.17854455820311	-1.94383659189631
C	2.72201730690243	8.83377971858365	-2.08227671558670
H	3.18871820569606	8.07598176221076	-1.45830101427306
N	0.26414285961804	6.75944520730152	-3.46043129442084
N	-0.47986554083653	5.66082971895296	-3.61697496445410

H	1.21426469000178	4.74753592237655	-1.34272736292608
H	1.28350355808179	5.88123614808888	2.78680638241140
H	0.73470727551895	8.22625814179742	2.15039890786755
C	0.66744650735759	7.79490363867423	0.05158328794972
H	0.45863657561257	8.82541960858195	-0.22167396154692
C	4.25288292117973	2.73181690880488	-0.26491178925549
C	4.13337141700584	1.36392877794805	-0.49858933100951
C	3.55393180579827	0.91546538980894	-1.68099940531707
H	3.46462391423119	-0.14945306141304	-1.87568808817563
C	3.08324690730335	1.82530542933840	-2.62133754167817
C	3.19756828709355	3.19920050638676	-2.39511600489959
C	2.77918483403712	4.19425185895010	-3.41544836507084
C	3.79106549659744	5.13343587860179	-3.93566582972766
C	3.41458915437603	6.35202155841157	-4.51612221001064
H	2.36750701231008	6.63134687265064	-4.52625786251354
C	4.37378168216441	7.21308513928585	-5.02982043347592
H	4.06145285419414	8.16799091126382	-5.44450239920219
C	5.72421534854266	6.87318688040241	-4.98264175676420
H	6.47401377453230	7.55267551127846	-5.37790515607113
C	6.10854617814621	5.66317138446218	-4.41012552535761
H	7.15857354723209	5.38794728071706	-4.37063351378513
C	5.15379546368807	4.80219485933883	-3.88435628974788
H	5.46191093926378	3.85830331451404	-3.44448221031102
N	1.57639995154406	4.26771928824212	-3.89310131495728
N	0.48895341695964	3.42575778313060	-3.61385584563293
H	2.64500108974843	1.46779376726463	-3.54542428609661
H	4.49485870426200	0.65115317576029	0.23703498329059
H	4.70724945464475	3.09191556019766	0.65373307018979
C	3.80021426251525	3.64265545031292	-1.21122295360024
H	3.90586317994717	4.70967048454571	-1.03590579672451

C. References

[1] Y. Xiong, S. Dong, S. Yao, C. Dai, J. Zhu, S. Kemper, M. Driess, "An Isolable 2,5-Disila-3,4-Diphosphapyrrole and a Conjugated Si=P−Si=P−Si=N Chain Through Degradation of White Phosphorus with a N,N -Bis(Silylenyl)Aniline" *Angewandte Chemie International Edition* **2022**, *61*, e202209250.

[2] Y.-P. Zhou, S. Raoufmoghaddam, T. Szilvási, M. Driess, "A Bis(silylene)-Substituted ortho - Carborane as a Superior Ligand in the Nickel-Catalyzed Amination of Arenes" *Angewandte Chemie International Edition* **2016**, *55*, 12868–12872.

[3] Y. Wang, A. Kostenko, S. Yao, M. Driess, "Divalent Silicon-Assisted Activation of Dihydrogen in a Bis(N-heterocyclic silylene)xanthene Nickel(0) Complex for Efficient Catalytic Hydrogenation of Olefins" *Journal of the American Chemical Society* **2017**, *139*, 13499–13506.

[4] E. Le Saux, E. Georgiou, I. A. Dmitriev, W. C. Hartley, P. Melchiorre, "Photochemical Organocatalytic Functionalization of Pyridines via Pyridinyl Radicals" *J. Am. Chem. Soc.* **2023**, *145*, 47–52.

[5] K. Okano, S. Ogino, M. Kawamoto, T. Yamashita, "Mass migration on a polymer surface caused by photoinduced molecular rotation" *Chem. Commun.* **2011**, *47*, 11891.

[6] G. M. Sheldrick, "Crystal structure refinement with SHELXL" *Acta Crystallographica Section C Structural Chemistry* **2015**, *71*, 3–8.

[7] L. J. Bourhis, O. V. Dolomanov, R. J. Gildea, J. A. K. Howard, H. Puschmann, "The anatomy of a comprehensive constrained, restrained refinement program for the modern computing environment – Olex2 dissected" *Acta Crystallographica Section A Foundations and Advances* **2015**, *71*, 59–75.

[8] O. V. Dolomanov, L. J. Bourhis, R. J. Gildea, J. A. K. Howard, H. Puschmann, "OLEX2 : a complete structure solution, refinement and analysis program" *Journal of Applied Crystallography* **2009**, *42*, 339–341.

[9] F. Neese, "Software Update: The ORCA Program System—Version 6.0" *WIREs Comput Mol Sci* **2025**, *15*, e70019.

[10] S. Grimme, A. Hansen, S. Ehlert, J.-M. Mewes, "r2SCAN-3c: A 'Swiss army knife' composite electronic-structure method" *The Journal of Chemical Physics* **2021**, *154*, 064103.

[11] E. Caldeweyher, S. Ehlert, A. Hansen, H. Neugebauer, S. Spicher, C. Bannwarth, S. Grimme, "A generally applicable atomic-charge dependent London dispersion correction" *The Journal of Chemical Physics* **2019**, *150*, 154122.

[12] J. G. Brandenburg, S. Grimme, "Accurate Modeling of Organic Molecular Crystals by Dispersion-Corrected Density Functional Tight Binding (DFTB)" *J. Phys. Chem. Lett.* **2014**, *5*, 1785–1789.

[13] F. Weigend, R. Ahlrichs, "Balanced basis sets of split valence, triple zeta valence and quadruple zeta valence quality for H to Rn: Design and assessment of accuracy" *Phys. Chem. Chem. Phys.* **2005**, *7*, 3297.

[14] A. V. Marenich, C. J. Cramer, D. G. Truhlar, "Universal Solvation Model Based on Solute Electron Density and on a Continuum Model of the Solvent Defined by the Bulk Dielectric Constant and Atomic Surface Tensions" *J. Phys. Chem. B* **2009**, *113*, 6378–6396.

[15] G. Mills, H. Jónsson, G. K. Schenter, "Reversible work transition state theory: application to dissociative adsorption of hydrogen" *Surface Science* **1995**, *324*, 305–337.

[16] G. Henkelman, B. P. Uberuaga, H. Jónsson, "A climbing image nudged elastic band method for finding saddle points and minimum energy paths" *The Journal of Chemical Physics* **2000**, *113*, 9901–9904.

[17] K. Fukui, "The path of chemical reactions - the IRC approach" *Acc. Chem. Res* **n.d.**, *14*, 363–368.

[18] G. Knizia, "Intrinsic Atomic Orbitals: An Unbiased Bridge between Quantum Theory and Chemical Concepts" *J. Chem. Theory Comput.* **2013**, *9*, 4834–4843.

[19] G. Knizia, J. E. M. N. Klein, "Electron Flow in Reaction Mechanisms—Revealed from First Principles" *Angew Chem Int Ed* **2015**, *54*, 5518–5522.
[20] A. Michalak, M. Mitoraj, T. Ziegler, "Bond Orbitals from Chemical Valence Theory" *J. Phys. Chem. A* **2008**, *112*, 1933–1939.
[21] M. Mitoraj, A. Michalak, "Applications of natural orbitals for chemical valence in a description of bonding in conjugated molecules" *J Mol Model* **2008**, *14*, 681–687.
[22] E. D. Glendening, J. K. Badenhoop, A. E. Reed, J. E. Carpenter, J. A. Bohmann, C. M. Marales, P. Karafiloglou, C. R. Landis, F. Weinhold, NBO 7.0, University of Wisconsin: Madison, WI, **2018**.
[23] K. Wolinski, J. F. Hinton, P. Pulay, "Efficient implementation of the gauge-independent atomic orbital method for NMR chemical shift calculations" *J. Am. Chem. Soc.* **1990**, *112*, 8251–8260.
